# New Series of Pyrazoles and Imidazo-Pyrazoles Targeting Different Cancer and Inflammation Pathways

**DOI:** 10.3390/molecules26195735

**Published:** 2021-09-22

**Authors:** Maria Grazia Signorello, Federica Rapetti, Elda Meta, Adama Sidibè, Olga Bruno, Chiara Brullo

**Affiliations:** 1 Biochemistry Lab., Department of Pharmacy, University of Genoa, Viale Benedetto XV 3, I-16132 Genova, Italy; signorello@difar.unige.it; 2 Section of Medicinal Chemistry, Department of Pharmacy, University of Genoa, Viale Benedetto XV 3, I-16132 Genova, Italy; federica.rapetti@edu.unige.it (F.R.); obruno@unige.it (O.B.); 3 Laboratory of Angiogenesis and Vascular Metabolism, Center for Cancer Biology, Vlaams Instituut Voor Biotechnologie, 3000 Leuven, Belgium; elda.meta.phd@gmail.com; 4 Department of Cell Physiology and Metabolism, University of Geneva, Rue Michel-Servet 1, CH-1211 Geneva, Switzerland; adama.sidibe@unige.ch

**Keywords:** pyrazoles, imidazo-pyrazoles, medicinal chemistry, ROS inhibition, platelet aggregation inhibition, p38MAPK, anti-angiogenesis compounds

## Abstract

(1) Background: different previously synthesized pyrazoles and imidazo-pyrazoles showed interesting anti-angiogenic action, being able to interfere with ERK1/2, AKT and p38MAPK phosphorylation in different manners and with different potency; (2) Methods: here, a new small compound library, endowed with the same differently decorated chemical scaffolds, has been synthetized to obtain new agents able to inhibit different pathways involved in inflammation, cancer and human platelet aggregation. (3) Results: most of the new synthesized derivatives resulted able to block ROS production, platelet aggregation and p38MAPK phosphorylation both in platelets and Human Umbilical Vein Endothelial cells (HUVEC). This paves the way for the development of new agents with anti-angiogenic activity.

## 1. Introduction

Although many innovative therapies have been introduced to date, a large increase in cancer mortality is estimated in the next few years [1]. In fact, despite this progress, the onset of resistance to therapy represents a major problem.

The mitogen-activated protein kinases (MAPK) are a wide family of serine–threonine protein kinases involved in a variety of cellular processes, such as proliferation, differentiation, cell survival and apoptosis [2]. MAPK are activated by many stimuli, such as osmotic stress, heat shock and inflammatory cytokines [3]. The signaling pathways involved in MAPK mechanisms lead to inflammation, which is a known pathogenesis factor of many chronic diseases, including cancer [4]. The MAPK family includes extracellular signal-regulated kinases (ERK1/2), c-Jun N-terminal kinases (JNK) and p38MAPK, which have particularly emerged as promising drug targets in recent years [2]. Given the contribution of inflammation to tumorigenesis, inhibition of p38MAPK signaling could reduce inflammation-associated cancers, but resistance to MAPK inhibitors is a current problem, particularly due to the high degree of interactions and possible compensatory responses [3,4].

Considering the crosstalk among different signaling pathways, targeted therapy, using small molecules as selective inhibitors of key signaling steps, should induce resistance, probably due to the activation of different mechanisms that can compensate the specific targeted pathway. On the contrary, the poly-pharmacology approach, using multitarget compounds acting on different cellular pathways involved in cancer progression, metastasis formation and tumor related-inflammation, seems to be an interesting strategy to reduce toxicity and resistance onset [5,6,7]. Consequently, combined therapies simultaneously targeting p38MAPK and other signaling pathways could represent a new interesting therapeutic approach [8].

Increasing evidence considers human platelets as common cellular elements of thrombosis, inflammation and cancer [9]. Regarding thrombosis, it is well known that human platelet activation, adhesion and aggregation are the first steps in thrombus formation at an injured vascular site and play a crucial role in primary hemostasis [10]. Although not strictly appointed within the inflammatory pathway, it is also well-established that platelets can be considered inflammatory cells, since they not only release a wide variety of inflammatory cytokines during platelet activation/aggregation [11], but also even increase the level of reactive oxygen species (ROS) at the site of the vascular injury [12]. Platelets, contributing to all steps of tumorigenesis [13], could be a suitable cellular model since it is widely accepted that there is a causal relationship between inflammation and cancer development [14].

Human platelets are known to contain ERK1/2 and p38MAPK, which are activated in response to agonists such as thrombin. Sakurai et al. [15] and Borst et al. [16] demonstrated that platelet activation/aggregation, as well thrombus formations, are modulated by the p38MAPK signaling pathway. Different compounds, including mainly natural products, have been recently reported as p38MAPK and platelet aggregation inhibitors [17,18].

Thus, ROS production and p38MAPK phosphorylation inhibition, both related to human platelet aggregation, could represent a good biological model to assay new synthesized compounds as potential anti-inflammatory and anti-cancer drugs.

**Figure 1:**
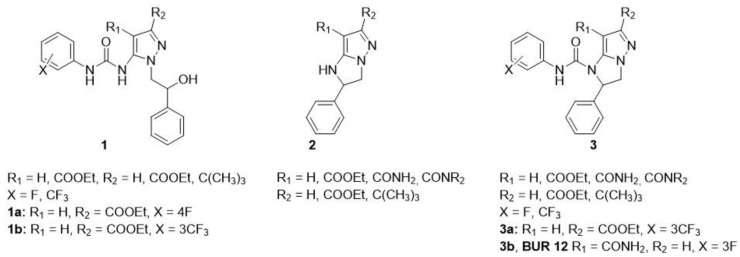
Structure of previous synthesized pyrazoles **1** and imidazo-pyrazoles **2**,*3*.

In the past, we synthesized different pyrazole and imidazo-pyrazole compounds able to inhibit neutrophil chemotaxis by interfering with ERK1/2, AKT and p38MAPK phosphorylation [19,20], and to block the angiogenesis process, acting on p38MAPK and PI3K signaling in VEGF-stimulated Human Umbilical Vein Endothelial cells (HUVEC) and in neuroblastoma cell lines [21,22] (Figure 1, compounds **1–3**).

Our results showed that some structural features, such as the type of substituent and their position on the pyrazole or imidazo-pyrazole scaffold and the flexibility loss in the imidazo-pyrazole derivatives in respect to pyrazole ones, serve as a switch between increasing or decreasing the phosphorylation levels of p38MAPK, ERK1/2 and AKT, and they also determine the activity potency. In detail, the most active compounds able to inhibit p38MAPK and ERK phosphorylation in VEGF-stimulated HUVEC cells were **1a**, **1b** and **3a**, all bearing a carboxyethyl substituent in C3 of the pyrazole nucleus or in C6 of the imidazo-pyrazole one, suggesting that the position of this group could be crucial for biological activity.

Since human platelets could represent a fast and low-cost biological model to screen compounds such as anticancer, anti-inflammatory and antiaggregating agents, we tested previous compounds **1a**, **1b** and **3a** to verify their inhibitory activity on human platelet aggregation, ROS production and p38MAPK phosphorylation. Interestingly, all three compounds showed multiple activities (see Results below), **1b** and **3a** being the most interesting, with IC_50_ values under 100 μM towards the three tested parameters**.** These new results, together with the previous ones already obtained, confirm that these derivatives could represent interesting lead compounds for the synthesis of new multitarget compounds able to interfere with different intracellular signaling involved in inflammation and cancer progression. 

**Figure 2:**
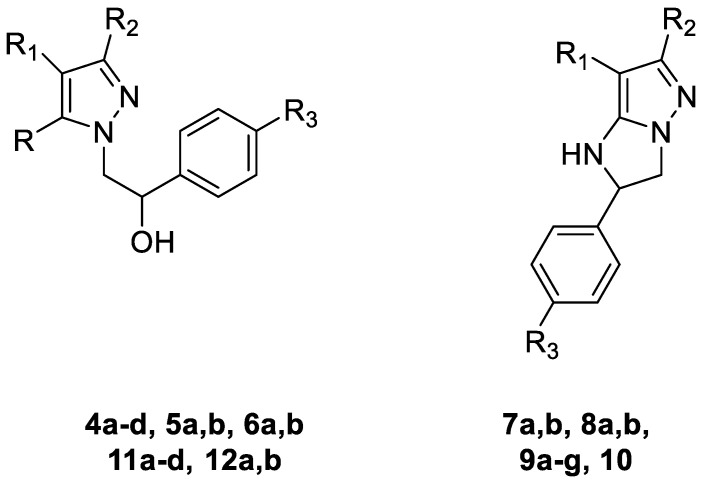
General structures of new synthesized compounds **4–10**.

Based on these results, another small library of compounds (**4–10** and the intermediates **11–12**, Figure 2, Table 1) has been designed and synthesized. 

In detail, in new designed compounds, we introduced different structural modifications:

(1) firstly, we synthesized both pyrazoles (compounds **4** and **6**), pyrrolyl-pyrazoles (compound **5**) and more rigid imidazo-pyrazoles (compounds **7–10**) to verify the role of molecule rigidity and the importance of the nitrogen basic atom able to form H bonds.
(2) in some cases in these chemical scaffolds, we introduced several amide groups (which, in the previous compound **2**, showed a good ability to block neutrophil chemotaxis and angiogenesis [19,21]) at different positions of the pyrazole nucleus (position 4, compounds **4** and **5**) and the imidazo-pyrazole one (position **6** or **7**, compound **9**); intermediates with carboxyethyl and carboxylic groups at different positions of pyrazoles, pyrrolyl-pyrazoles and imidazo-pyrazoles were also tested to verify the role of these substituents and their position for biological activity (compounds **7**, **8**, **11d**, **12b**).
(3) since in all previous series, fluorine derivatives showed the most efficient potency, probably due to an increasing cell membrane crossing, in some cases, we introduced a more lipophilic fluorine atom in the para-position of the phenyl ring (compounds **6a**, **6b**, **7a**, **8a**, **9a**,**b** and **10**).

To clarify the role of these different substituents on varying intracellular pathways, the inhibitory effect on human platelet aggregation, ROS production and p38MAPK phosphorylation has been preliminary evaluated. The obtained results are reported in Table 2.

## 2. Results and Discussion

### 2.1. Chemistry

**Scheme 1:**
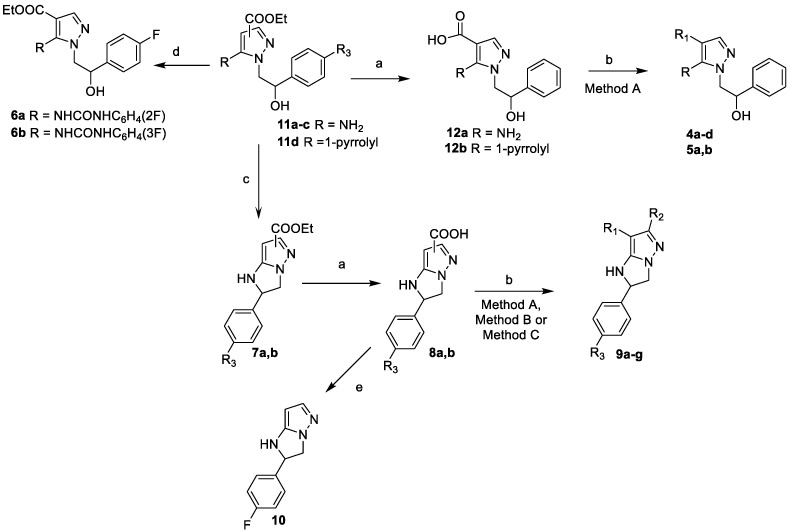
Synthesis of compounds **4–10**. Reagents and conditions: (**a**) 2M or 3.5M NaOH, EtOH, reflux, 5 h; (**b**) Method A: suitable amine, TEA, DPPA, an. DMF, 30–60 °C, 12 h or microwave irradiation, 300W, under pressure, 90 °C, 5 min (**9d**); Method B: suitable amine, EDC, 4-(dimethylamino)pyridine**,** CH_2_Cl_2_, 0 °C, then r.t. 24 h (**9f**, **9g**); Method C: SOCl_2_, CH_2_Cl_2_, 0 °C, then an. DMF, TEA, suitable amines 0 °C, then 40–50 °C, 6 h (**9a**, **9c**, **9e**); (**c**) conc. H_2_SO_4_, 0 °C, 15 min, then NH_3_ r.t.; (**d**) suitable fluorophenyl isocyanate, an. toluene, reflux, 6 h; (**e**) thermal decarboxylation.

Compounds **4–10** were synthesized as reported in Scheme 1, starting from key intermediate pyrazoles **11a–d**, as previously reported (**11a**: [23], **11b**: [24]; **11c**: [21], **11d**: [25]). In detail, to obtain derivatives **4** and **5**, **11a** and **11d** were hydrolyzed to give carboxylic derivative **12a**,**b**, as previously reported (**12a**: [26]; **12b**: [25]). The latter ones react with an excess of a suitable amine in the presence of diphenylphosphorylazide (DPPA) in an. DMF to obtain the amide derivatives **4a–d** and **5a**,**b** (Scheme 1).

Compounds **6a** and **6b**, characterized by a urea moiety at position 5 of the pyrazole nucleus, as previously shown in **1a**,**b**, were synthesized starting from **11b** [24], treated with a little excess of suitable fluorophenyl isocyanate in anhydrous toluene at reflux. 

The imidazo-pyrazole scaffold of derivatives **7–10** was obtained by dehydration with concentrated sulfuric acid of the 4-carboxyethyl-5-amino-pyrazoles **11b** and **11c** to give ethyl 2-phenyl-2,3-dihydro-1*H*-imidazo [1,2-*b*]pyrazole-7-carboxylates **7a,b** [21]. Then, the carboxyethyl function of **7** was hydrolyzed to give the corresponding carboxylic derivatives **8a**,**b**. To obtain the final compound **9**, different synthetic procedures were performed because of low yields obtained, probably attributable to the low reactivity of the carboxylic acid **8**. In fact, the use of DDPA in an. DMF (Method A, as performed for compounds **4**, **5** and the previous **2**) [19] led to acceptable yields only for compounds **9b** and **9d** (for which an alternative microwave under the pressure heating method was used to prevent the evaporation of the corresponding amines). Compounds **9f** and **9g** were synthesized in the presence of 1-ethyl-3-(3-dimethylaminopropyl)carbodiimide (EDC) in DCM (Method B). Finally, **8a** and **8b** were first transformed into the corresponding acyl chlorides with SOCl_2_, then reacted with cyclopropylamine to obtain the desired compounds **9a**, **9c** and **9e** with acceptable yields (Method C).

Finally, compound **10** was obtained by the thermal decarboxylation of **8a**. 

### 2.2. Inhibition Effect on Human Platelet Aggregation, ROS Production and p38MAPK Phosphorylation

**Figure 3:**
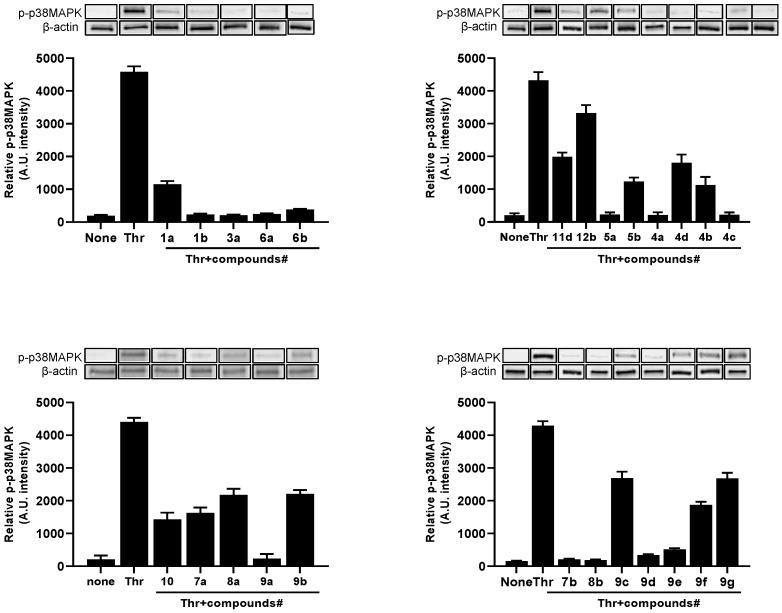
p38MAPK phosphorylation in human platelets. Washed platelets (1.0 × 10^9^ platelets/mL) were preincubated at 37 °C with saline or compounds and then stimulated for 5 min with 0.1 U/mL thrombin (Thr). At the end of incubation, suitable aliquots were immunoblotted with anti-p-p38MAPK, as detailed in Methods. Blots are representative of six independent experiments. In the lower panels, densitometric scanning ± SD of p38MAPK phosphorylation of six experiments is reported. Image of inhibiting effect of reference compound **SB203580** is reported in Supplementary Materials.

Compounds were tested on human platelets to verify their inhibitory activity on platelet aggregation, ROS production and p38MAPK phosphorylation. As reported in Table 2 and Figure 3, all the new synthesized compounds showed interesting inhibitory effect on the three tested parameters. Particularly, pyrazoles **4a** and **6a** and imidazo-pyrazole **8b** are the most significant, with the IC_50_ values towards aggregation, ROS production and p38MAPK phosphorylation being under 100 μM for all of them, and thus with potency comparable to the previous synthesized compounds **1b** and **3a**. Nonetheless, even **4c**, **5a**, **6b**, **7b**, **9a**, **9d** and **9e** are noteworthy compounds, with the IC_50_ being just higher than 100 μM. All compounds were compared to **SB203580**, a specific p38MAPK inhibitor [27], which is obviously more efficient than the newly synthetized compounds, and to **N-acetylcysteine** (NAC) [28], a known thiol-reducing agent and ROS scavenger that inhibits ROS production.

### 2.3. SAR Considerations

Compounds **1b** and **3a**, previously active in VEGF-stimulated HUVEC cells [21], also showed a good pharmacological profile in human platelets. The new urea derivatives **6a** and **6b**, structurally similar to **1a**,**b**, showed a significant inhibitory activity, with IC_50_ values lower than 100 μM in platelet aggregation, ROS production and p38 phosphorylation, evidencing the most interesting biological profile of the pyrazole derivatives. 

In addition, 5-amino-pyrazole **4a** (bearing a cyclopropylamide moiety in position 4 instead of a carboxyethyl one) showed interesting and multitarget biological activity, whereas compounds **5a**,**b** and the corresponding intermediates **11d** and **12b**, characterized by a pyrrole substituent at position 5, resulted in being less active; these data confirmed that the presence of a free NH_2_ group able to form H bonds is fundamental for biological properties. It is, however, worth noting that **5a** seems to possess a significant inhibitory effect, particularly on ROS production (IC_50_ about 70 μM), and less potency in platelet aggregation and p38 phosphorylation inhibition (IC_50_ values 105 and 109 µM, respectively), resulting in being the most selective of the series.

Regarding imidazo-pyrazole compounds, the carboxylic acid **8b** and carboxamide derivatives **9d** and **9e** showed the best inhibitory profiles, confirming that the presence of a substituent on C6 on the imidazo-pyrazole scaffold is fundamental to increase the activity. In fact, C7-substituted analogues (**8a**, **9a**, **9b**) or the unsubstituted derivative **10** showed a small decrease in potency.

### 2.4. Anti-Proliferative Activity Evaluation

To verify if some of these derivatives, in addition to previous biological activity, could also exert a non-specific anti-proliferative action, all new synthesized compounds were submitted to a large screening at NCI (National Cancer Institute, Germantown MD, USA). It is a very broad analysis of the anti-proliferative action, considering the most common cancers in adults, including both highly metastatic and more aggressive cell lines. As expected, all compounds did not show a strong anti-proliferative action, thus demonstrating that these new derivatives could exert some anticancer activity through an anti-inflammatory/antioxidant action, as previously reported for compounds **1**–**3**. Interestingly, urea derivatives **6a,b** exerted some anti-proliferative activity; in detail, **6a** showed a slight inhibition (25–30% at a 10 μM concentration) of melanoma (SK-MEL-5, UACC-62) and renal cancer cell lines (UO-31 cancer cell lines), whereas **6b** evidenced a large spectrum of action, with a 25–30% of inhibition in leukemia (CCFR-CEM, RPMI-8226), non-small cell lung (NCI-H522), CNS (SF-295, SNB-75), ovarian (OVCAR-4) and breast (BT-549, MDA-MB-468) cancer cell lines, and with a 50–60% inhibition in melanoma (SK-MEL-5, UACC-62), renal cancer (CAK-1, UO-31) and prostate cancer (PC-3) cell lines.

### 2.5. p38 Phosphorylation Inhibition on HUVEC Cells VEGF-Stimulated

The VEGF/VEGFR axis induces key events fundamental in the angiogenesis process; in detail, VEGF induces these functions through the activation of several serine/threonine kinases in the phosphatidylinositol 3-kinase (PI3K) signaling pathway, such as protein kinases B (AKT/PKB) and MAPK signaling, including p38MAPK and ERK1/2.

**Figure 4:**
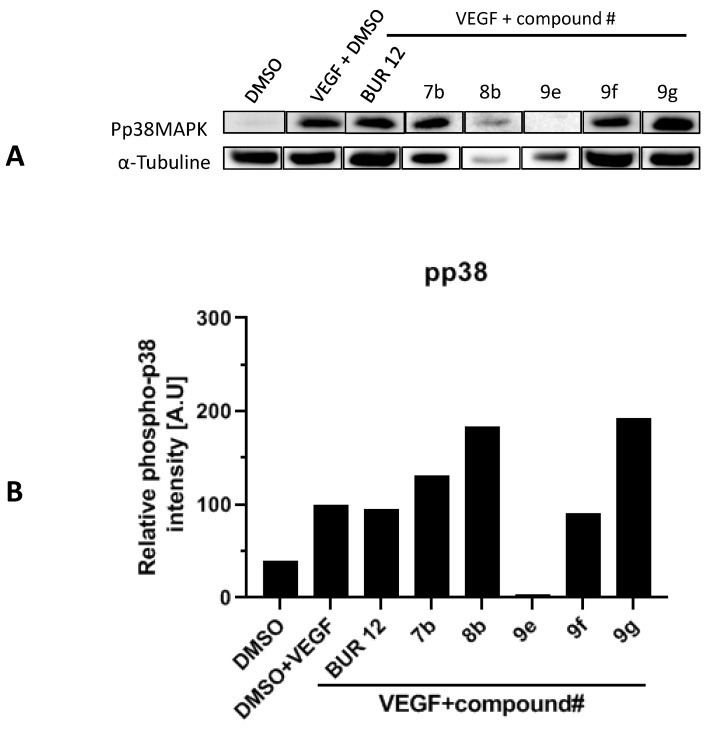
Representative Western blotting analyses of p38MAPK phosphorylation. HUVEC were pre-incubated with the compounds (20 μM) for 10 min and then stimulated with both VEGF and the compounds for 20 min. DMSO was used as the control. The proteins were extracted and analyzed by Western blotting; a representative band of at least two independent experiments is reported. (**A**): the densiometric analyses were performed with ImageJ; (**B**): tubulin was used as the protein-loading control.

**Figure 5:**
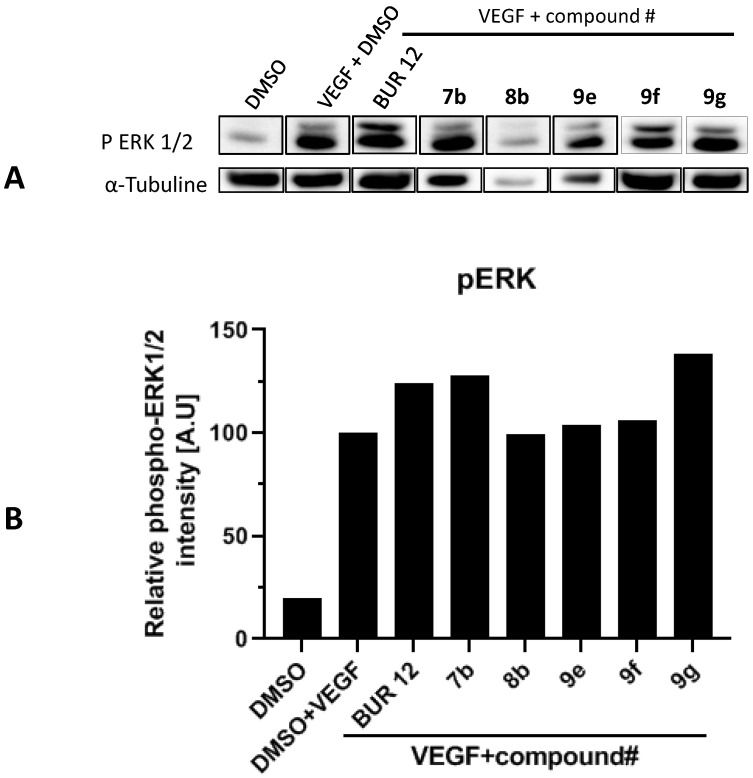
Representative Western blotting analyses of ERK1/2 phosphorylation. HUVEC were pre-incubated with the compounds (20 μM) for 10 min and then stimulated with both VEGF and the compounds for 20 min. DMSO was used as control. The proteins were extracted and analyzed by Western blotting; representative bands of at least two independent experiments are reported. (**A**): the densiometric analyses were performed with ImageJ; (**B**): tubulin was used as the protein-loading control.

**Figure 6:**
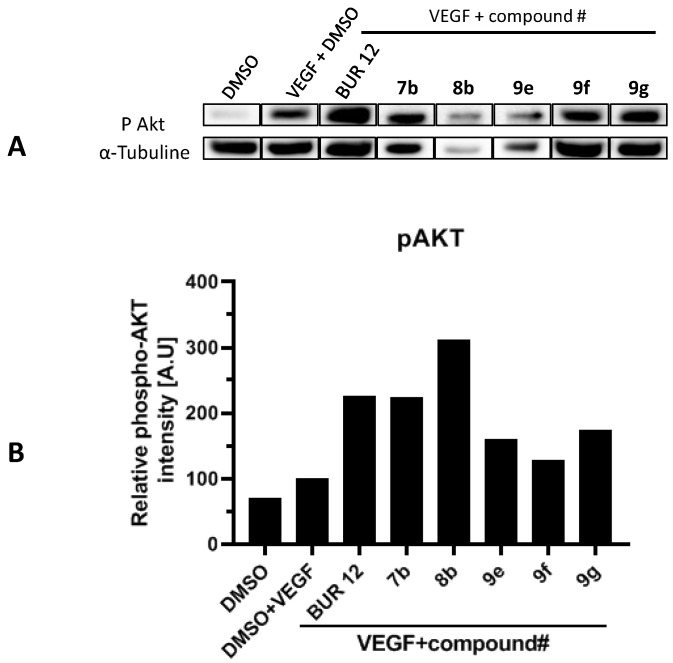
Representative Western blotting analyses of AKT phosphorylation. HUVEC were pre-incubated with the indicated compounds (20 μM) for 10 min and then stimulated with both VEGF and the compounds for 20 min. DMSO was used as control. The proteins were extracted and analyzed by Western blotting; representative bands of at least two independent experiments are reported (**A**). The densiometric analyses were performed with ImageJ (**B**). Tubulin was used as the protein-loading control.

Because previous derivatives **2** and **3** showed an interesting anti-angiogenic profile in VEGF-stimulated HUVEC cells, especially inhibiting p38MAPK phosphorylation, we selected some imidazo-pyrazoles, **7b**, **8b** (among the most active in the human platelet model), and some of derivative **9** (in detail, **9e**, **9f** and **9g**, bearing chemical similarity with the previous **2** and **3** [21]) to verify their possible interaction at the intracellular level with different pathways involved in cancer and the angiogenesis process. In detail we have tested the effect of the selected new synthetized compound (at a 20 μM concentration) on p38MAPK, ERK and AKT phosphorylation on VEGF-stimulated HUVEC cells (Figure 4, Figure 5 and Figure 6). In addition, we tested, as lead compound, previously synthetized **BUR 12** (**3a**, Figure 2) [21], endowed with the same imidazo-pyrazole scaffold of compounds **7–9** and similar fluoro-urea moiety of derivative **6**. **BUR 12** recently emerged as a new interesting compound able to counteract the ability of cancer cell lines (HTLA-230) to form capillary-like structures [22]. Our aim is to confirm if this nucleus could be as interesting as antiangiogenic compound, as previously reported.

From these biological studies, imidazo-pyrazole **9e** emerged as the most interesting, being able to strongly inhibit p38MAPK phosphorylation (Figure 4), but not ERK1/2 and AKT phosphorylation (Figure 5 and Figure 6). Particularly, **9e** showed the same behavior of our lead **BUR 12,** but, interestingly, it evidenced a greater ability to reduce p38MAPK phosphorylation. This particular selectivity, together with its activity in inhibiting p38MAPK phosphorylation in human platelets, confirmed its action on blocking this specific intracellular pathway, and it is certainly noteworthy.

## 3. Materials and Methods

### 3.1. General Information

Chiminord and Aldrich Chemical (Milan, Italy) purchased all chemicals. Solvents were reagent grade. All commercial reagents were used without further purification. Aluminum-backed silica gel plates (Merck DC-Alufolien Kieselgel 60 F254, Darmstad, Germany) were used in thin-layer chromatography (TLC) for routine monitoring of the reaction course. The detection of spots was made by UV light. Merck silica gel, 230–400 mesh, was used for chromatography. Flash chromatography was performed using Isolera one instrument (Biotage, Uppsala, Sweden) using a Silicagel column. Melting points were not “corrected” and were measured with a Buchi M-560 instrument (Buchi instruments, Flawil, Switzerland). IR spectra were recorded with a Perkin-Elmer 398 spectrophotometer (Perkin-Elmer, Milan, Italy) or Spectrum Two FT-IR Spectrometer (PerkinElmer, Inc., Waltham, MA, USA). ^1^H NMR spectra were recorded on a Varian Gemini 200 (200 MHz, Varian Gemini, Palo Alto, CA, USA); chemical shifts are reported as δ (ppm) relative to tetramethylsilane (TMS) as internal standard; signals were characterized as s (singlet), d (doublet), t (triplet), n t (near triplet), q (quartet), m (multiplet), br s (broad signal), J in Hz. ^13^C NMR spectra were recorded on a BRUKER DPX-300 (75 MHz, Ettlingen, Germany) or a JEOL JNM ECZ-400S/L1 (101 MHz, Tokyo, Japan). Microwave apparatus: CEM Discover (CEM Corporation, Matthews, NC, USA), a single-mode microwave oven, with a max emitted power of 300 Watts, temperature-controlled by an optical fiber, with magnetic stirring of the sample and an air-cooling system.

Elemental analysis was determined with an elemental analyzer EA 1110 (Fison-Instruments, Milan, Italy); products are considered pure when the difference between calculated and found values is ±0.4 (see Supplementary Materials).

### 3.2. Synthesis

#### 3.2.1. General Procedure for Synthesis of Compounds **4a–d** and **5a,b**

The suitable amine (20 mmol), triethylamine (2 mL) and DPPA (3.4 g, 12 mmol) were slowly added to a solution of compounds **12a** or **12b** (10 mmol) in anhydrous DMF (10 mL) at 0 °C. Then, the mixture was stirred at 30–60 °C for 12 h. After cooling, iced water (200 mL) was added and the solution was made acidic with 1 M HCl. The crude solids obtained were filtered, washed with water and, if necessary, purified by column chromatography on Silicagel (using a mixture of CH_2_Cl_2_/CH_3_OH 9/1 as the eluent). Finally, the white solids obtained were recrystallized from absolute ethanol.

##### 5-Amino-N-cyclopropyl-1-(2-hydroxy-2-phenylethyl)-1*H*-pyrazole-4-carboxamide (**4a**)

Light yellow solid. Yield 52%; mp 171–172 °C. ^1^H-NMR (DMSO-d_6_): δ 3.10–3.20 (m, 4H, 2CH_2_ cycloprop.), 3.84–4.10 (m, 3H, CH_2_N pyraz. + *CH*NH), 4.81–4.97 (m, 1H, *CH*OH), 5.64–5.70 (m, 1H, OH, disappears with D_2_O), 6.04 (br s, 2H, NH_2_, disappears with D_2_O), 7.18–7.42 (m, 6H, 5Ar + H-3) and 11.70 (br s, 1H, NH, disappears with D_2_O). ^13^C NMR (DMSO-d_6_): δ 165.80, 151.21, 143.11, 139.34, 128.63, 127.02, 126.71, 94.90, 72.00 and 54.58. IR (KBr): cm^−1^ 3449, 3346, 3237 (OH, NH) and 1692 (CONH). Anal. (C_15_H_18_N_4_O_2_) calcd for C, H and N.

##### (5-Amino-1-(2-hydroxy-2-phenylethyl)-1*H*-pyrazol-4-yl)(piperidin-1-yl)methanone (**4b**)

Pale rose solid. Yield 50%; mp 166–167 °C. ^1^H-NMR (DMSO-d_6_): δ 1.30–1.60 (m, 6H, 3CH_2_ pip.), 3.20–3.30 and 3.40–3.60 (2m, 4H, 2CH_2_N pip.), 3.80–4.06 (m, 2H, CH_2_N pyraz.), 4.82–4.96 (m, 1H, *CH*OH), 5.66 (br s, 1H, OH, disappears with D_2_O), 5.90 (br s, 2H, NH_2_, disappears with D_2_O) and 7.18–7.48 (m, 6H, 5Ar + H-3). ^13^C NMR (DMSO-d_6_): δ 164.83, 151.25, 143.17, 137.52, 128.63, 127.92, 126.69, 96.73, 72.14, 54.64, 26.77 and 24.60. IR (KBr): cm^−1^ 3421, 3317, 3223 (OH, NH) and 1679 (CON). Anal. (C_17_H_22_N_4_O_2_) calcd for C, H and N.

##### (5-Amino-1-(2-hydroxy-2-phenylethyl)-1*H*-pyrazol-4-yl)(morpholino)methanone (**4c**)

White solid. Yield 37%; mp 135–137 °C. ^1^H-NMR (DMSO-d_6_): δ 2.98–3.20 (m, 2H, CH_2_N morph.), 3.30–3.41 (m, 2H, CH_2_N morph.), 3.42–3.52 (m, 4H, 2CH_2_O morph.), 3.83–4.17 (m, 2H, CH_2_N pyraz.), 4.81–5.00 (m, 1H, *CH*OH), 5.70 (br s, 1H, OH, disappears with D_2_O), 6.00 (br s, 2H, NH_2_, disappears with D_2_O) and 7.00–7.50 (m, 6H, 5Ar + H-3). ^13^C NMR (DMSO-d_6_): δ 165.21, 151.41, 143.15, 137.68, 128.64, 127.92, 126.70, 96.22, 72.11, 66.77 and 54.61. IR (KBr): cm^−1^ 3432, 3321, 3264 (OH, NH) and 1704 (CON). Anal. (C_16_H_20_N_4_O_3_) calcd for C, H and N.

##### 5-Amino-N-cyclopentyl-1-(2-hydroxy-2-phenylethyl)-1*H*-pyrazole-4-carboxamide (**4d**)

Ivory solid. Yield 32%; mp 169–170 °C. ^1^H-NMR (CDCl_3_): δ 0.70–0.81 (m, 4H, 2CH_2_ cyclopent.), 1.82–2.09 (m, 4H, 2CH_2_ cyclopent.), 4.00–4.22 (m, 3H, CH_2_N pyraz. + *CH*NH), 4.50–4.60 (m, 1H, *CH*OH), 5.00 (br s, 2H, NH_2_, disappears with D_2_O), 7.12–7.38 and 7.40–7.70 (2m, 6H, 5Ar) and 8.08 (s, 1H, H-3). ^13^C NMR (CDCl_3_): δ 164.46, 150.12, 142.40, 138.77, 128.78, 128.56, 127.25, 98.98, 72.92, 55.74, 55.74, 51.70, 32.93 and 23.25. IR (KBr): cm^−1^ 3429, 3328 (OH, NH) and 1689 (CONH). Anal. (C_17_H_22_N_4_O_2_) calcd for C, H and N.

##### N-Cyclopropyl-1-(2-hydroxy-2-phenylethyl)-5-(1H-pyrrol-1-yl)-1*H*-pyrazole-4-carboxamide (**5a**)

White solid. Yield 55%; mp 147–148 °C. ^1^H-NMR (CDCl_3_): δ 1.96–2.27 (m, 4H, 2CH_2_ cycloprop.), 2.62–2.80 (m, 1H, *CH*NH), 3.90–4.20 (m, 2H, CH_2_N pyraz.), 4.83–4.95 (m, 1H, *CH*OH), 5.18 (br s, 1H, disappears with D_2_O), 6.20–6.50 (m, 4H, 2H-2 and 2H-5 pyrr.), 7.15–7.50 (m, 5H Ar) and 8.14 (s, 1H, H-3). ^13^C NMR (DMSO-d_6_): δ 153.00, 138. 53, 130.35, 125.27, 120.57, 120.53, 110.00, 23.33 and 6.81. IR (KBr): cm^−1^ 3413, 3233 (OH + NH) and 1642 (CONH). Anal. (C_19_H_20_N_4_O_2_) calcd for C, H and N.

##### (1-(2-Hydroxy-2-phenylethyl)-5-(1H-pyrrol-1-yl)-1*H*-pyrazol-4-yl)(piperidin-1-yl)methanone (**5b**)

White solid. Yield 41%; mp 134–135 °C. ^1^H-NMR (CDCl_3_): δ 1.06–1.30 (m, 2H, CH_2_ pip.), 1.40–1.61 (m, 4H, 2CH_2_ pip.), 2.94–3.31 (m, 2H, CH_2_N pip.), 3.40–3.70 (m, 2H, CH_2_N pip.), 4.10–4.16 (m, 2H, CH_2_N pyraz.), 5.04–5.12 (m, 1H, *CH*OH), 6.31 (d, *J* = 2.0, 2H, H-2 pyrr.), 6.61 (d, *J* = 2.0, 2H, H-5 pyrr.), 7.13–7.41 (m, 5H Ar) and 7.76 (s, 1H, H-3). ^13^C NMR (DMSO-d_6_): δ 165.22, 143.02, 138.32, 137.28, 128.78, 128.07, 126.26, 123.61, 110.73, 71.41, 55.23, 40.64 and 24.38. IR (KBr): cm^−1^ 3265 (OH) and 1610 (CON). Anal. (C_21_H_24_N_4_O_2_) calcd for C, H and N.

#### 3.2.2. Synthesis of Ethyl 1-(2-(4-fluorophenyl)-2-hydroxyethyl)-5-(3-(2-fluorophenyl)ureido)-1*H*-pyrazole-4-carboxylate (**6a**) and 1-(2-(4-fluorophenyl)-2-hydroxyethyl)-5-(3-(3-fluorophenyl)ureido)-1*H*-pyrazole-4-carboxylate **6b**

A mixture of pyrazole **11b** [24] (293 mg, 1 mmol) and the suitable fluorophenyl isocyanate (151 mg, 1.1 mmol) in anhydrous toluene (7 mL) was refluxed for 12 h. After cooling to room temperature, the solution was extracted with diethyl ether, washed with 3 M HCl (2 ×20 mL) and water (10 mL), dried (MgSO_4_) and evaporated under reduced pressure. The crude was crystallized by adding a solution of diethyl ether/petroleum ether (b.p. 50–60 °C) (1:1). The white solids obtained were recrystallized from absolute ethanol.

##### Ethyl 1-(2-(4-fluorophenyl)-2-hydroxyethyl)-5-(3-(2-fluorophenyl)ureido)-1*H*-pyrazole-4-carboxylate (**6a**)

White solid. Yield 32%; mp 177–178 °C. ^1^H-NMR (CDCl_3_): δ 1.49 (t, *J* = 7.2, 3H, CH_3_), 4.31–4.57 (m, 4H, CH_2_O + CH_2_N pyraz.), 5.20–5.38 (m, 1H, *CH*OH), 6.19 (br s, 1H, disappears with D_2_O), 7.09–7.32 and 7.40–7.57 (2m, 8H, Ar), 7.66 (s, 1H, H-3) and 8.11 (br s, 1H, disappears with D_2_O). ^13^C NMR (CDCl_3_): δ 164.64, 162.67, 153.73, 151.34, 143.76, 139.83, 129.23, 124.78, 123.89, 121.13, 115.57, 102.47, 72.47, 60.15, 57.96 and 14.31. IR (KBr): cm^−1^ 3448, 3345 (NH + OH), 1712 (COOEt) and 1681 (CONH). Anal. (C_21_H_20_F_2_N_4_O_4_) calcd for C, H and N.

##### Ethyl 1-(2-(4-fluorophenyl)-2-hydroxyethyl)-5-(3-(3-fluorophenyl)ureido)-1*H*-pyrazole-4-carboxylate (**6b**)

White solid. Yield 45%; mp 198–200 °C. ^1^H-NMR (CDCl_3_): δ 1.50 (t, *J* = 7.2, 3H, CH_3_), 4.28–4.57 (m, 4H, CH_2_O + CH_2_N pyraz.), 5.18–5.33 (m, 1H, *CH*OH), 6.17 (br s, 1H, disappears with D_2_O), 6.88–7.55 (2m, 8H, Ar) and 7.77 (s, 1H, H-3). ^13^C NMR (CDCl_3_): δ 164.59, 162.62, 161.44, 153.24, 142.01, 140.93, 140.22, 138,15, 130.44, 128.65, 115.69, 113.18, 111.04, 105.38, 102.02, 72.38, 60.12, 57.98 and 14.28. IR (KBr): cm^−1^ 3427, 3321 (NH + OH), 1705 (COOEt) and 1678 (CONH). Anal. (C_21_H_20_F_2_N_4_O_4_) calcd for C, H and N.

#### 3.2.3. General Procedure for Synthesis of Compounds **7a,b**

Compound **7b** was previously reported [21]. **7a** was prepared following the same procedure and dissolving **11b** in concentrated sulfuric acid (10 mL) at 0 °C; then, the mixture was stored at room temperature for 15 min. Finally, iced water (600 mL) was added and the solution was made neutral with NH_4_OH solution; the white solid obtained was filtered, washed with water and recrystallized from absolute ethanol. 

##### Ethyl 2-(4-fluorophenyl)-2,3-dihydro-1*H*-imidazo[1,2-*b*]pyrazole-7-carboxylate (**7a**)

White solid. Yield 66%; mp 153–154 °C. ^1^H-NMR (CDCl_3_): δ 1.45 (t, *J* = 7.0, 3H, CH_3_), 4.08 (n t, 1H, H-3), 4.35 (q, *J* = 7.0, 2H, CH_2_O), 4.68 (n t, 1H, H-3), 5.59 (n t, 1H, H-2), 7.16–7.36 and 7.50–7.67 (2m, 4H, Ar) and 7.83 (s, 1H, H-6). ^13^C NMR (CDCl_3_): δ 163.16, 161.47, 145.81, 139.93, 137.62, 127.90, 115.70, 89.99, 67.05, 60.12, 57.41 and 14.28. IR (KBr): cm^−1^ 3328 (NH) and 1665 (COOEt). Anal. (C_14_H_14_FN_3_O_2_) calcd for C, H and N.

#### 3.2.4. General Procedure for Synthesis of Compounds **8a,b**

A solution of compound **7a** or **7b** (10 mmol) in 2 M NaOH solution (30 mL) was stirred at 120 °C for 4 h. After cooling to room temperature, iced water was added, and then acetic acid was added until pH 5. The grey solid obtained was filtered, washed with water and recrystallized from CH_2_Cl_2_/absolute ethanol (1:1).

##### 2-(4-Fluorophenyl)-2,3-dihydro-1*H*-imidazo[1,2-*b*]pyrazole-7-carboxylic acid (**8a**)

Yield 53%; mp 187–190 °C. ^1^H-NMR (DMSO-d_6_): δ 3.95 (n t, 1H, H-3), 4.74 (n t, 1H, H-3), 5.60 (n t, 1H, H-2), 7.26–7.48 and 7.50–7.76 (2m, 5H, 4Ar + H-6) and 11.98 (br s, 1H, COOH disappears with D_2_O). ^13^C NMR (CDCl_3_): δ 164.06, 163.16, 161.19, 151.53, 139.27, 137.59, 127.90, 115.70, 90.45, 67.05 and 57.36. IR (KBr): cm^−1^ 3237–3100 (NH + OH) and 1649 (COOH). Anal. (C_12_H_10_FN_3_O_2_) calcd for C, H and N

##### 2-Phenyl-2,3-dihydro-1*H*-imidazo[1,2-*b*]pyrazole-6-carboxylic acid (**8b**)

Yield 90%; mp 212–213 °C. ^1^H-NMR (DMSO-d_6_): δ 3.87 (n t, 1H, H-3), 4.65 (n t, 1H, H-3), 5.37 (n t, 1H, H-2), 5.71 (1H, H-7), 6.61 (br s, 1H, NH, disappears with D_2_O), 7.18–7.84 (m, 5H, Ar) and 11.90 (br s, 1H, COOH disappears with D_2_O). ^13^C NMR (CDCl_3_): δ 162.53, 157.95, 140.64, 139.89, 128.34, 128.20, 126.01, 83.01, 67.72 and 56.32. IR (KBr): cm^−1^ 3390–2550 (NH + OH) and 1693 (COOH). Anal. (C_12_H_11_N_3_O_2_) calcd for C, H and N

#### 3.2.5. General Procedure for Synthesis of Compounds **9a–g**

##### Method A (Compounds **9b** and **9d**)

The suitable amine (20 mmol), triethylamine (2 mL) and DPPA (3.3 g, 12 mmol) were slowly added to a solution of compounds **8a** or **8b** (10 mmol) in anhydrous DMF (10 mL) at 0 °C. Then, the mixture was stirred at 30–60 °C for 12 h for compound **9b**. To obtain compound **9d,** the mixture was irradiated with microwaves under pressure, increasing the emitted power until 300W for 5 min, and at the same time cooling by compressed air to avoid the temperature increasing over 100 °C; then, the mixture was cooled to room temperature with compressed air for 1 min. Finally, iced water (200 mL) was added and the solution was made acidic with 1 M HCl. The crude solids obtained were filtered, washed with water and, if necessary, purified by Silicagel column chromatography (using a mixture of CH_2_Cl_2_/CH_3_OH 9/1 as the eluent). Finally, the white solids obtained were recrystallized from absolute ethanol.

##### Method B (Compounds **9f** and **9g**)

1-ethyl-3-(3-dimethylaminopropyl)carbodiimide (EDC) (2.09 g, 13 mmol), 4-(dimethylamino)pyridine (122 mg, 1 mmol) and suitable amine (10 mmol) were added to a solution of compound **8b** (2.3 g, 10 mmol) in CH_2_Cl_2_ (10 mL) at 0 °C, and the mixture was stirred at room temperature for 24 h. Then, the mixture was washed with saturated solution NaHCO_3_ (2 × 10 mL) and water (2 × 20 mL), dried (MgSO_4_) and concentrated under reduced pressure. The crude was purified by Silicagel column chromatography (using a mixture of CH_2_Cl_2_/CH_3_OH 9/1 as the eluent) and crystallized from ethyl ether.

##### Method C (Compounds **9a**, **9c** and **9e**)

To a solution of compounds **8a** or **8b** (1 mmol) in CH_2_Cl_2_ (5 mL) at 0 °C, thionyl chloride (0.5 mL, 6 mmol) was slowly added; then, the mixture was heated at reflux for 30 min. After evaporation under vacuum, the crude was dissolved in anhydrous DMF (5 mL); then, triethylamine (2 mL) and an excess of suitable amine (10 mmol) were slowly added at 0 °C. The mixture was heated at 30–50 for 6 h. After cooling to room temperature, iced water (50 mL) was added and the solution was extracted with ethyl acetate (10 mL); the organic phases was washed with water (2 × 10 mL), dried (MgSO_4_) and concentrated under reduced pressure. The crude obtained was purified by flash Silicagel column chromatography (using a mixture of CH_2_Cl_2_/CH_3_OH 9/1 as the eluent) to obtain a solid, subsequently re-crystallized from ethyl ether.

##### N-Cyclopropyl-2-(4-fluorophenyl)-2,3-dihydro-1*H*-imidazo[1,2-*b*]pyrazole-7-carboxamide (**9a**)

White solid. Yield 44%; mp 208–210 °C. ^1^H-NMR (CDCl_3_): δ 2.60–2.70 (m, 4H, 2CH_2_ cycloprop.), 2.88–2.92 (m, 1H, *CH*NH), 3.90 (n t, 1H, H-3), 4.88 (n t, 1H, H-3), 5.56 (n t, 1H, H-2), 7.18 (br s, 1H, NH disappears with D_2_O), 7.30–7.42 and 7.60–7.75 (2m, 4H, Ar) and 7.79 (s, 1H, H-6). ^13^C NMR (DMSO-d_6_): δ 165.22, 160.00, 157.79, 142.46, 138.45, 129.37, 115.93, 115.70, 96.63, 64.74, 53.33, 24.40 and 22.40. IR (KBr): cm^−1^ 3305 (NH) and 1626 (CONH). Anal. (C_15_H_15_FN_4_O) calcd for C, H and N.

##### (2-(4-Fluorophenyl)-2,3-dihydro-1*H*-imidazo[1,2-*b*]pyrazol-7-yl)(piperidin-1-yl)methanone (**9b**)

Ivory solid. Yield 44%; mp 156–158 °C. ^1^H-NMR (CDCl_3_): δ 1.55–1.86 (m, 6H, 3CH_2_ pip.), 3.60–3.77 (m, 4H, 2CH_2_N pip.), 3.95 (n t, 1H, H-3), 4.72 (n t, 1H, H-3), 5.53 (n t, 1H, H-2), 7.06 (br s, 1H, NH disappears with D_2_O), 7.28–7.43 and 7.50–7.70 (2m, 5H, 4Ar + H-6). ^13^C NMR (CDCl_3_): δ 166.42, 163.13, 161.19, 151.40, 137.62, 136.60, 127.90, 115.70, 95.21, 67.05, 56.98, 45.80, 25.74 and 24.51. IR (KBr): cm^−1^ 3385 (NH) and 1610–1591 (CON). Anal. (C_17_H_19_FN_4_O) calcd for C, H and N.

##### N-Isopropyl-2-phenyl-2,3-dihydro-1*H*-imidazo[1,2-*b*]pyrazole-6-carboxamide (**9c**)

White solid. Yield 42%; mp 118–121 °C. ^1^H-NMR (DMSO-d_6_): δ 1.05–1.20 (m, 6H, 2CH_3_ isoprop.), 2.80–3.00 (m, 1H, *CH*NH), 3.25 (n t, 1H, H-3), 3.60 (n t, 1H, H-3), 5.20 (n t, 1H, H-2), 5.90 (s, 1H, H-7) and 6.92–7.41 (m, 6H, 5Ar + NH, 1H disappears with D_2_O). ^13^C NMR (CDCl_3_): δ 162.67, 154.73, 145.09, 140.17, 128.58, 127.72, 126.02, 83.19, 67.87, 56.63, 42.22 and 22.79. IR (KBr): cm^−1^ 3409 (NH) and 1649 (CONH). Anal. (C_15_H_18_N_4_O) calcd for C, H and N.

##### N-Isobutyl-2-phenyl-2,3-dihydro-1*H*-imidazo[1,2-*b*]pyrazole-6-carboxamide (**9d**)

White solid. Yield 52%; mp 116–120 °C. ^1^H-NMR (DMSO-d_6_): δ 0.74–1.00 (m, 6H, 2CH_3_ isobut.), 1.70–1.91 (m, 1H, *CH*isobut.), 2.90–3.11 (m, 2H, CH_2_ isobut.), 3.83 (n t, 1H, H-3), 4.62 (n t, 1H, H-3), 5.40 (n t, 1H, H-2), 5.64 (s, 1H, H-7), 6.60 (br s, 1H, disappears with D_2_O), 6.12–7.58 (m, 5H Ar) and 7.93 (br s, 1H, NH disappears with D_2_O). ^13^C NMR (DMSO-d_6_): δ 163.22, 154.11, 151.22, 141.69, 129.11, 128.38, 127.17, 83.00, 64.74, 53.76, 46.27, 28.68 and 20.64. IR (KBr): cm^−1^ 3448 (NH) and 1670 (CONH). Anal. (C_16_H_20_N_4_O) calcd for C, H and N.

##### N-Cyclopropyl-2-phenyl-2,3-dihydro-1*H*-imidazo[1,2-*b*]pyrazole-6-carboxamide (**9e**)

Ivory solid. Yield 47%; mp 135–136 °C. ^1^H-NMR (CDCl_3_): δ 0.77–1.01 (m, 4H, 2CH_2_ cycloprop.), 1.72–1.90 (m, 1H, *CH*NH), 3.80 (n t, 1H, H-3), 4.60 (n t, 1H, H-3), 5.35 (n t, 1H, H-2), 5.60 (s, 1H, H-7), 6.60 (s, 1H, NH disappears with D_2_O), 6.20–7.60 (m, 5H, Ar) and 7.90–7.93 (br s, 1H, NH disappears with D_2_O). ^13^C NMR (CDCl_3_): δ 166.73, 154.73, 144.99, 140.17, 128.58, 127.72, 126.02, 83.19, 67.87, 56.63, 23.40 and 7.43. IR (KBr): cm^−1^ 3309, 3233 (NH) and 1646 (CONH). Anal. (C_15_H_16_N_4_O) calcd for C, H and N.

##### (2-Phenyl-2,3-dihydro-1*H*-imidazo[1,2-*b*]pyrazol-6-yl)(piperidin-1-yl)methanone (**9f**)

White solid. Yield 30%; mp 202–203 °C. ^1^H-NMR (DMSO-d_6_): δ 1.38–1.78 (m, 6H, 3CH_2_ pip.), 3.45–3.61 (m, 2H, CH_2_N pip.), 3.77–3.94 (m, 3H, CH_2_N pip. + H-3), 4.62 (n t, 1H, H-3), 5.28 (n t, 1H, H-2), 5.56 (s, 1H, H-7), 6.58 (s, 1H, NH disappears with D_2_O) and 7.28–7.57 (m, 5H, Ar). ^13^C NMR (DMSO-d_6_): δ 163.44, 153.66, 151.09, 143.33, 128.66, 127.56, 125.00, 84.62, 64.55, 53.01, 48.23, 42.23, 27.20, 25.33 and 24.12. IR (KBr): cm^−1^ 3226 (NH) and 1601 (CON). Anal. (C_17_H_20_N_4_O) calcd for C, H and N.

##### Morpholino(2-phenyl-2,3-dihydro-1*H*-imidazo[1,2-*b*]pyrazol-6-yl)methanone (**9g**)

Pale pink solid. Yield 40%; mp 162–163 °C. ^1^H-NMR (CDCl_3_): δ 3.50–3.70 (m, 4H, 2CH_2_N morph.), 3.85 (n t, 1H, H-3), 3.90–4.18 (m, 4H, 2CH_2_O morph.), 4.63 (n t, 1H, H-3), 5.37 (n t, 1H, H-2), 5.63 (s, 1H, H-7), 6.60 (br s, 1H, NH disappears with D_2_O) and 7.25–7.58 (m, 5H, Ar). ^13^C NMR (DMSO-d_6_): δ 164.04, 153.36, 150.66, 142.23, 129.12, 128.40, 127.71, 85.45, 66.28, 64.79 and 53.89. IR (KBr): cm^−1^ 3239 (NH) and 1606 (CON). Anal. (C_16_H_18_N_4_O_2_) calcd for C, H and N.

#### 3.2.6. Synthesis of 2-(4-fluorophenyl)-2,3-dihydro-1*H*-imidazo[1,2-*b*]pyrazole (**10**)

Compound **8a** (2.47 gr, 10 mmol) was heated at 190 °C until the complete development of CO_2_. The crude was solved in DCM (30 mL), washed twice with saturated NaHCO_3_ solution (2 × 20 mL) and dried (MgSO_4_). After solvent evaporation, the pale solid obtained was recrystallized from absolute ethanol.

Pale pink solid. Yield 90%; mp 131–133 °C. ^1^H-NMR (CDCl_3_): δ 3.85 (n t, 1H, H-3), 4.60 (n t, 1H, H-3), 5.35 (n t, 1H, H-2), 5.74 (br s, 1H, NH disappears with D_2_O), 6.60 (d, *J* = 3.0, 1H, H-6) and 6.90–7.60 (m, 6H, 5Ar). ^13^C NMR (CDCl_3_): δ 163.16, 161.19, 149.34, 137.48, 136.70, 127.97, 115.70, 85.74, 67.56 and 56.44. IR (KBr): cm^−1^ 3167 (NH). Anal. (C_11_H_10_FN_3_) calcd for C, H and N.

### 3.3. Biological Studies

#### 3.3.1. Material

Colorburst™ electrophoresis marker, 2′,7′-dichlorofluorescein diacetate (DCFH-DA), **N-acetylcysteine** (NAC), SB203580 and thrombin were purchased from Sigma-Aldrich/Merck Millipore. Anti p-p38MAPK, horseradish peroxidase-conjugated secondary antibodies and anti-β-actin were purchased from Santa Cruz Biotechnology, USA. The ECL^®^ system was from GE Healthcare, North Richland Hills, TX, USA. Nitrocellulose membranes (pore size 0.45 µm) were from Bio-Rad Laboratories, Hercules, CA USA.

#### 3.3.2. Blood Collection and Preparative Procedures

Freshly drawn venous blood from healthy volunteers of the “Centro Trasfusionale, Ospedale San Martino” in Genoa was collected into 130 mM aqueous trisodium citrate anticoagulant solution (9:1). The donors claimed to have not taken drugs known to interfere with platelet function during the two weeks prior to blood collection and gave their informed consent. Washed platelets were prepared by centrifuging whole blood at 100× *g* for 20 min. The obtained platelet-rich plasma was then centrifuged at 1100× *g* for 15 min. The pellet was washed once with pH 5.2 ACD solution (75 mM trisodium citrate, 42 mM citric acid and 136 mM glucose), centrifuged at 1100× *g* for 15 min and then re-suspended in pH 7.4 Hepes buffer (145 mM NaCl, 5 mM KCl, 1 mM MgSO_4_, 10 mM glucose, 10 mM HEPES).

#### 3.3.3. p38MAPK Phosphorylation in Human Platelet

Washed platelets (1.0 × 10^9^/mL), preincubated with saline, compounds or SB203580 (SB), were stimulated with 0.1 U/mL thrombin for 5 min. Incubation was stopped by adding 2×Laemmli-SDS reducing sample buffer. The samples, heated for 5 min at 100 °C, were separated by 5–10% SDS-PAGE and transferred to nitrocellulose membranes. Running was performed in the presence of Colorburst™ Electrophoresis weight markers. Blots were blocked in 5% BSA, dissolved in TBST (Tris buffer saline, pH 7.6, containing 10 mM Tris, 150 mM NaCl, and 0.1% Tween 20) at 37 °C for 30 min, and then incubated overnight at 4 °C with anti-p-p38MAPK (1:1000 dilutions) antibody. Membranes were extensively washed and incubated for 60 min at room temperature with horseradish peroxidase-conjugated secondary antibody. After further washings, blots were developed using the ECL^®^ system. Nitrocellulose membranes were then stripped by incubation with 62.5 mM Tris/HCl (pH 6.7), 2% SDS and 100 µM β-mercaptoethanol for 30 min at 50 °C and reprobed with anti-β-actin. Band density was directly quantified by the Bio-Rad Chemi-Doc software package. The reported IC_50_ value is the molar concentration of the compound able to obtain 50% inhibition of the maximal aggregation induced by the agonist and is calculated by the percentage of inhibition that is the inhibition of the maximal aggregation measured in the presence of the agent, compared with that measured in a control sample containing saline carried out under the same conditions.

#### 3.3.4. ROS Assay

ROS production was quantified as previously reported [29] by DCFH-DA, an ROS-sensitive probe that yields, upon oxidation, the fluorescent adduct DCF that is trapped inside the cells. Briefly, washed platelets (1.0 × 10^8^/mL) pre-incubated with saline, compounds or **N-acetylcysteine** (NAC) for 15 min at 37 °C were stimulated by 0.1 U/mL thrombin. Incubation was stopped by cooling samples in an ice bath and then samples were immediately analyzed in a Merck Millipore Bioscience Guava easyCyte flow cytometer (Merk Millipore, Burlington, MA, USA). The IC_50_ values were calculated as detailed above.

#### 3.3.5. Platelet Aggregation

Platelet aggregation was performed in a Bio-Data Aggregometer (Bio-Data Corporation, Horsham, PA, USA) according to Born’s method [30] and quantified by the light transmission reached within 6 min at 37 °C. Briefly, washed platelets (3.0 × 10^8^/mL) were pre-incubated for 3 min at 37 °C with saline, compounds or SB203580 before the addition of 0.1 U/mL thrombin. The IC_50_ values were calculated as detailed above.

#### 3.3.6. Anti-Proliferative Activity

Testing was performed by the Developmental Therapeutics Program, Division of Cancer Treatment and Diagnosis, National Cancer Institute [31].

#### 3.3.7. Western Blotting on HUVEC Cells—VEGF Stimulated

HUVEC were seeded in a 6-well plate pre-coated with 0.2% gelatin and Collagen G at 0.1 mg/mL in PBS. The cells were cultured in M199 (GIBCO) supplemented with 10% fetal calf serum, 1% Endothelial Cell Growth Supplement (EmdMillipore), 0.1 mg/mL of heparin sodium, 0.1 mM of hydrocortisone (Sigma Aldrich, Milan, Italy), 1% antibiotic glutamine mixture and 0.1% of vitamin C for 48 h to become confluent. Confluent HUVEC were starved for at least 4 h with the medium alone before stimulation. After starvation, the cells were pre-incubated with the indicated compound at 20 μM for 10 min at 37 °C and then stimulated with both VEGF at 50 ng/mL and the compound at 20 mM for 20 min. As the compounds were dissolved in dimethylsulfoxyde (DMSO), incubation with DMSO was used as a control to the compounds. Treated cells were washed and lysed for protein extraction. Protein concentration was determined with the MicroBCA™ Protein Assay Kit (Thermo Scientific, Waltham, MA USA). Equal amounts of proteins (30 mg) were subjected to a gel electrophoresis and then transferred to nitrocellulose blotting membranes. Membranes were blocked with a blocking buffer made of PBS containing 5% not-fat dry milk and 0.05% of Tween 20. Membranes were incubated overnight at 4 °C with primary antibodies diluted in the blocking buffer. The following primary antibodies were used in this study: rabbit anti-phospho-p38MAPK (pp38MAPK) used at 1:1000 dilution, rabbit anti-phospho-ERK1/2 (pERK1/2) (cell signaling) at 1:1000 dilution, rabbit anti-phospho-AKT (pAKT) at 1:1000 dilution and mouse–tubuline antibody (Millipore) at 1:4000 dilution. Membranes were washed three times for 5 min with PBS-Tween 20 0.05% and incubated at room temperature for 1 h with the adequate secondary antibody. Horseradish peroxidase (HRP)-coupled goat anti-rabbit antibody was used at a dilution of 1:10000 and the HRP-goat anti-mouse antibody at 1:3000 dilution. Both secondary antibodies were from Jackson ImmunoResearch. Membranes were washed and the signals detected using the enhanced chemioluminescence system (Advansta, WesternBright™ Sirius). Protein band intensities were quantified with ImageJ and the tubuline intensity was used to ensure equal loaded protein amounts. The results were expressed relative to the condition of treatment with VEGF and DMSO serving as control in the same blot and set at 100%. Data are representative of at least two independent experiments.

## 4. Conclusions

All tested compounds showed the ability to interfere with ROS production and p38MAPK phosphorylation with different potency in human platelet, evidencing a potential dual anti-inflammatory and anticancer mechanism of action. In fact, is well known that cancer is a multifactorial disease, in which inflammation (strictly related to p38MAPK phosphorylation inhibition) [14] and ROS production play an important role, particularly during metastasis formation [32], and that compounds with non-classical anti-proliferative action could represent an alternative therapy.

As reported in our previous studies, SAR considerations demonstrated a pivotal role of the position of carboxyethyl or carboxamide both in the pyrazole and imidazo-pyrazole scaffold; in fact, while in pyrazole derivatives the best activity was observed in C4-substituted compounds (as in **6a,b** and **4a**), in imidazo-pyrazole ones C6-substituted compounds (as **8b**, **9d** and **9e**) are generally more active than C7 analogs (**8a**, **9a**, **9b**). In addition, when no substituents were present at these two positions (compound **10**), the inhibitory effect on all the three tested parameters decreases.

Urea derivatives **6a,b**, most similar to the previous **1**, showed a very interesting profile, being able to strongly inhibit ROS production, platelet aggregation and p38MAPK phosphorylation; in addition, they also evidenced certain anti-proliferative activity in different cancer cell lines. These results confirmed that urea moiety, inserted in our substituted pyrazole scaffold, could represent an interesting starting point in the development of new derivatives endowed with multitarget action.

Compounds **9c**–**g**, more strictly related to the previous **2** and **3** and endowed with an amide function at position 6 of the imidazo-pyrazole scaffold, showed less activity (IC_50_ values between 80 and 300 μM), but as expected, they are able to interfere with p38MAPK pathways in platelet and also in VEGF-stimulated HUVEC cells. Particularly compound **9e** emerged as the most interesting of the series, resulting in being more active than **BUR 12**, previously reported as a promising antiangiogenic derivative [22].

These multiple activities and variable potency degrees may be partially explained by the crosstalk between different signaling pathways involved in cancer progression, metastasis formation and angiogenesis process.

In conclusion, all these results confirmed that both these two molecular scaffolds (pyrazole and imidazo-pyrazole), if differently decorated in all positions, could provide new potential multitarget anticancer/anti-inflammatory agents able to act at the intracellular level in different ways, by blocking ROS production and inflammation, and by interfering with phosphorylation of kinases normally involved and/or hyperactivated in cancer.

New investigation will be necessary to investigate the in vivo anti-angiogenic action of these interesting classes of molecules.

## Figures and Tables

**Table 1:** Decorations on the new synthesized compounds **4–10** and intermediates **11** and **12**.

Compd.	R	R_1_	R_2_	R_3_
**4a**	NH_2_	CO-cyclopropylamino	H	H
**4b**	NH_2_	CO-piperidino	H	H
**4c**	NH_2_	CO-morpholino	H	H
**4d**	NH_2_	CO-cyclopentylamino	H	H
**5a**	1-pyrrolyl	CO-cyclopropylamino	H	H
**5b**	1-pyrrolyl	CO-piperidino	H	H
**6a**	NHCONHC_6_H_4_(2)F	COOEt	H	F
**6b**	NHCONHC_6_H_4_(3)F	COOEt	H	F
**7a**	//	COOEt	H	F
**7b**	//	H	COOEt	H
**8a**	//	COOH	H	F
**8b**	//	H	COOH	H
**9a**	//	CO-cyclopropylamino	H	F
**9b**	//	CO-piperidino	H	F
**9c**	//	H	CO-isopropylamino	H
**9d**	//	H	CO-isobutylamino	H
**9e**	//	H	CO-cyclopropylamino	H
**9f**	//	H	CO-piperidino	H
**9g**	//	H	CO-morpholino	H
**10**	//	H	H	F
**11a**	NH_2_	COOEt	H	H
**11b**	NH_2_	COOEt	H	F
**11c**	NH_2_	H	COOEt	H
**11d**	1-pyrrolyl	COOEt	H	H
**12a**	NH_2_	COOH	H	H
**12b**	1-pyrrolyl	COOH	H	H

**Table 2:** Inhibitory effect of previous compounds **1a,b**, **3a**, new synthesized compounds **4–10** and intermediates **11d** and **12b** and reference compounds **SB203580** and **NAC** on platelet aggregation, ROS production and p38MAPK phosphorylation on human platelets, expressed as IC_50_ (μM) values. Reported data are the mean +/- SD obtained in at least six different experiments, each performed in duplicate.

Compound	Aggregation Inhibition IC_50_ (μM)	ROS Production Inhibition IC_50_ (μM)	p-p38MAPK Inhibition IC_50_ (μM)
**1a**	157.45 ± 10.38	156.24 ± 25.71	133.66 ± 13.52
**1b**	85.11 ± 6.15	73.00 ± 23.82	95.09 ± 8.92
**3a**	95.36 ± 7.65	71.17 ± 12.07	89.35 ± 5.21
**4a**	83.89 ± 3.36	74.86 ± 6.18	95.73 ± 4.31
**4b**	124.12 ± 6.56	150.87 ± 0.38	135.34 ± 10.47
**4c**	99.07 ± 1.60	71.53 ± 8.65	105.20 ± 8.45
**4d**	146.77 ± 17.27	158.96 ± 6.09	171.10 ± 17.21
**5a**	104.72 ± 4.22	69.91 ± 12.06	108.76 ± 6.76
**5b**	136.57 ± 13.85	173.22 ± 8.37	139.22 ± 7.94
**6a**	97.45 ± 5.58	86.44 ± 15.07	95.83 ± 6.45
**6b**	98.25 ± 7.25	92.22 ± 8.64	90.09 ± 1053
**7a**	156.55 ± 21.19	174.08 ± 0.32	157.80 ± 9.58
**7b**	107.74 ± 2.18	65.65 ± 18.10	97.52 ± 10.52
**8a**	195.79 ± 14.69	210.82 ± 18.86	194.85 ± 5.87
**8b**	76.37 ± 2.47	77.83 ± 6.53	90.07 ± 5.14
**9a**	109.35 ± 8.18	82.56 ± 1.05	100.97 ± 8.62
**9b**	205.56 ± 14.70	242.52 ± 14.16	200.65 ± 5.36
**9c**	225.91 ± 15.95	314.87 ± 19.13	261.46 ± 15.68
**9d**	86.39 ± 3.68	80.90 ± 0.53	109.06 ± 9.24
**9e**	106.06 ± 6.49	86.40 ± 6.49	115.75 ± 4.83
**9f**	148.12 ± 4.94	144.41 ± 8.64	178.29 ± 8.88
**9g**	210.03 ± 8.91	198.90 ± 8.83	265.79 ± 11.62
**10**	141.94 ± 8.93	122.17 ± 9.46	146.79 ± 4.87
**11d**	213.46 ± 4.59	248.19 ± 11.08	184.00 ± 9.25
**12b**	305.34 ± 12.66	372.7 ± 19.8	428.76 ± 16.57
**SB203580**	19.51 ± 1.83	ND	13.31 ± 1.64
**NAC**	ND	964.23 ± 48.51	ND

## Data Availability

Not applicable.

## Supplementary Materials

The following are available online. ELEMENTAL ANALYSIS of most active compounds **4a–d**, **5a****,b**, **6a,b**, **7a**, **8a,b** and **9a–g**, **10**. ^1^H NMR, IR and ^13^C NMR spectra of compounds **4**, **5**, **6**, **7a**, **8**, **9**, **10**. Figure S1: Immunoblotting densitometric image of p38MAPK phosphorylation in human platelets preincubated at 37 °C with saline or SB203580 (SB) used as reference compound at two concentrations (10 and 20 μM).

## Sample Availability

Samples of the compounds **1a,b**, **3a**, **4–12** are available from the authors.

## Abbreviations

AKT	protein kinase B
DPPA	diphenylphosphorylazide
EDC	1-ethyl-3-(3-dimethylaminopropyl)carbodiimide
ERK	extracellular signal regulated kinase
fMLP	Formyl-methyl-leucyl-phenylalanine
HUVEC	Human umbilical vein endothelial
IL-8	Interleukine-8
NAC	n-acetylcysteine
PI3K	phosphatidylinositol 3-kinase
p38 MAPK	p38 Mitogen-Activated Protein Kinase
ROS	Reactive oxygen species
SAR	Structure–activity relationship
Thr	Thrombin
VEGF	Vascular endothelial growth factor
VEGFR	Vascular endothelial growth factor receptor
