# Supplementary material for: New Series of Pyrazoles and Imidazo-Pyrazoles Targeting Different Cancer and Inflammation Pathways
Source: Molecules. 2021 Sep 22;26(19):5735. doi: 10.3390/molecules26195735 (PMC8510017; doi:10.3390/molecules26195735)
Supplement: Supplementary file 1 [file molecules-26-05735-s001.zip › molecules-1393365-supplementary.pdf]

New series of Pyrazoles and Imidazo-pyrazoles targeting different cancer and inflammation pathways

Maria Grazia Signorello[1], Federica Rapetti[2], Elda Meta[3], Adama Sidibè[4], Olga Bruno[2] and Chiara Brullo[2,*]

1 Department of Pharmacy, Biochemistry Lab., University of Genoa, Viale Benedetto XV 3, I-16132 Genova, Italy

2 Department of Pharmacy, Section of Medicinal Chemistry, University of Genoa, Viale Benedetto XV 3, I-16132 Genova, Italy;

3 Laboratory of Angiogenesis and Vascular Metabolism, Center for Cancer Biology, Vlaams Instituut voor Biotechnologie, Leuven, Belgium;

4 Department of Cell physiology and Metabolism, University of Geneva, Rue Michel-Servet 1, CH - 1211, Geneva.

INDEX:

ELEMENTAL ANALYSIS of most active compounds 4a-d, 5a-b, 6a,b, 7a, **8a,b** and **9a-g, 10**.

^{1}H NMR, IR and ^{13}C NMR spectra of compounds 4, 5, 6, 7a, 8, 9, 10.

Figure 1: Immunoblotting densitometric image of p38MAPK phosphorylation in human platelets preincubated at 37°C with saline or SB203580 (SB) used as reference compound at two concentrations (10 and 20 μM).

ELEMENTAL ANALYSIS of most active compounds 4a-d, 5a-b, 6a,b, 7a, 8a,b and **9a-g, 10**.

Compd	Elemental analysis (%, calculated/ found)		
	**N**	**C**	**H**
**4a**	19.57<br>19.24	62.92<br>62.77	6.34<br>6.28
**4b**	17.82<br>18.08	64.95<br>64.65	7.05<br>6.96
**4c**	17.71<br>17.76	60.75<br>60.88	6.37<br>6.31
**4d**	17.82<br>17.53	64.95<br>64.61	7.05<br>6.96
**5a**	16.66<br>16.41	67.84<br>67.81	5.99<br>6.10
**5b**	15.37<br>15.60	69.21<br>69.07	6.64<br>6.42
**6a**	13.02<br>13.03	58.60<br>58.48	4.68<br>5.04
**6b**	13.02<br>13.24	58.60<br>58.33	4.68<br>4.83
**7a**	15.26<br>15.04	61.08<br>61.09	5.13<br>5.35
**8a**	17.00<br>17.22	58.30<br>58.21	4.08<br>4.20
**8b**	18.33	62.87	4.84

	18.63	62.91	4.53
**9a**	19.57<br>19.95	62.93<br>62.94	5.28<br>5.28
**9b**	17.82<br>17.56	64.95<br>64.52	6.09<br>6.22
**9c**	20.73<br>20.53	66.64<br>66.30	6.71<br>6.45
**9d**	19.70<br>19.50	67.58<br>67.36	7.09<br>7.05
**9e**	20.88<br>20.69	67.15<br>67.28	6.01<br>6.20
**9f**	18.90<br>18.83	68.90<br>68.69	6.80<br>7.19
**9g**	18.78<br>18.47	64.41<br>64.69	6.08<br>6.27
**10**	20.68<br>21.05	65.01<br>65.00	4.96<br>5.07

^{1}H NMR, IR and ^{13}C NMR spectra of compounds 4, 5, 6, 7a, 8, 9, 10.

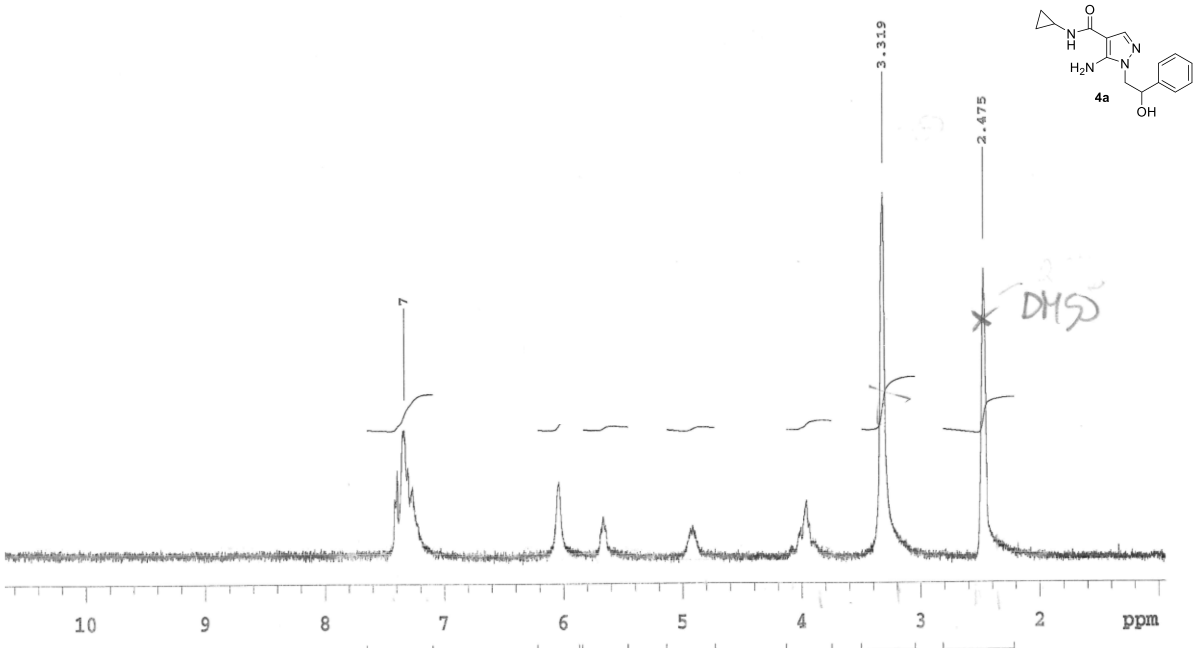

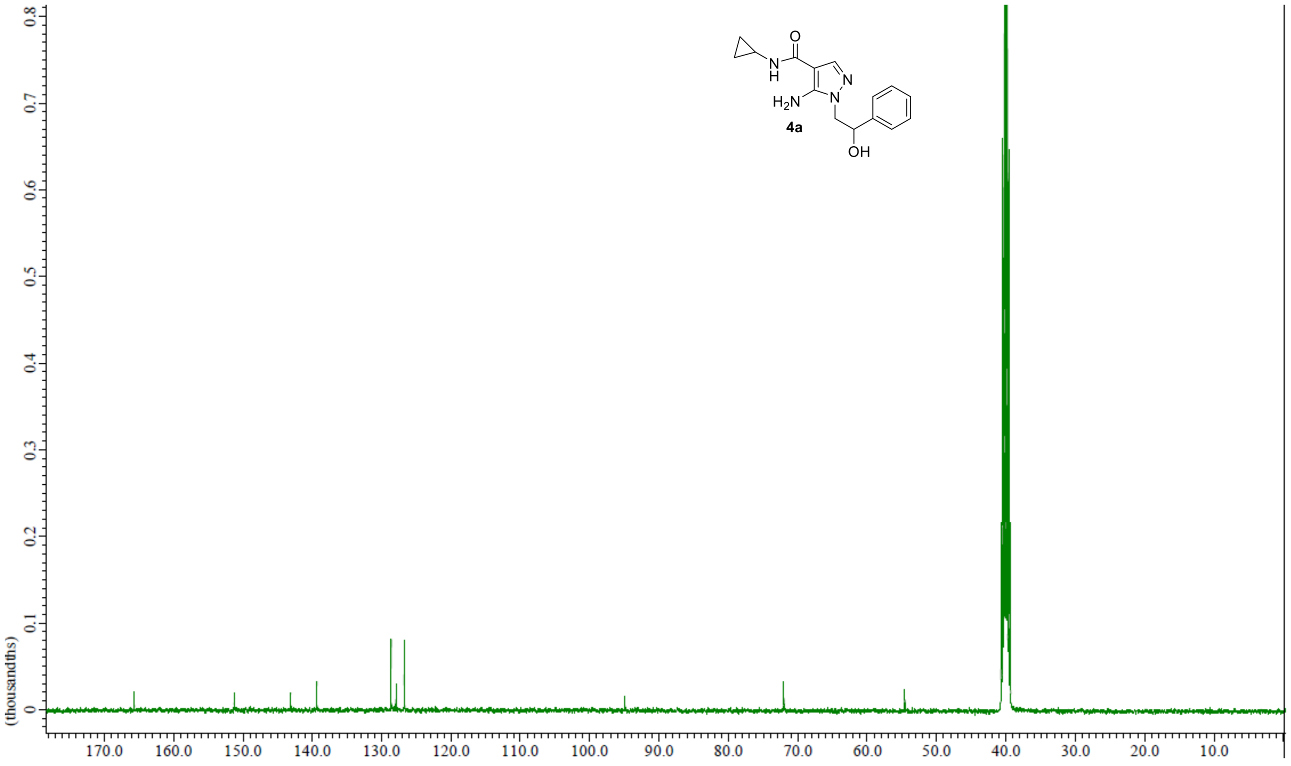

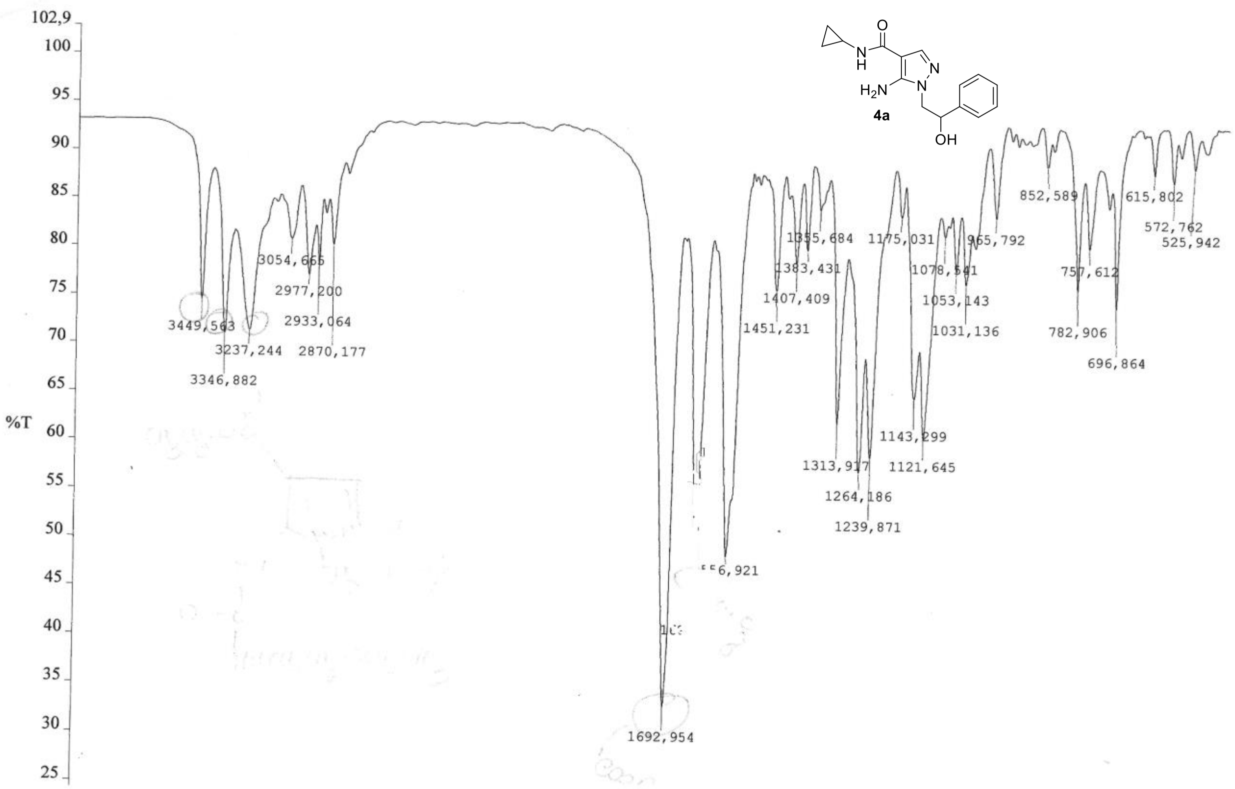

102,9
100
95
90
85
80
75
70
65
%T
60
55
50
45
40
35
30
25
4a
3449,563
3346,882
3237,244
3054,665
2977,200
2933,064
2870,177
1692,954
1451,231
1407,409
1383,431
1355,684
1313,917
1264,186
1239,871
1175,031
1143,299
1121,645
1078,541
1053,143
1031,136
965,792
852,589
782,906
757,612
696,864
615,802
572,762
525,942

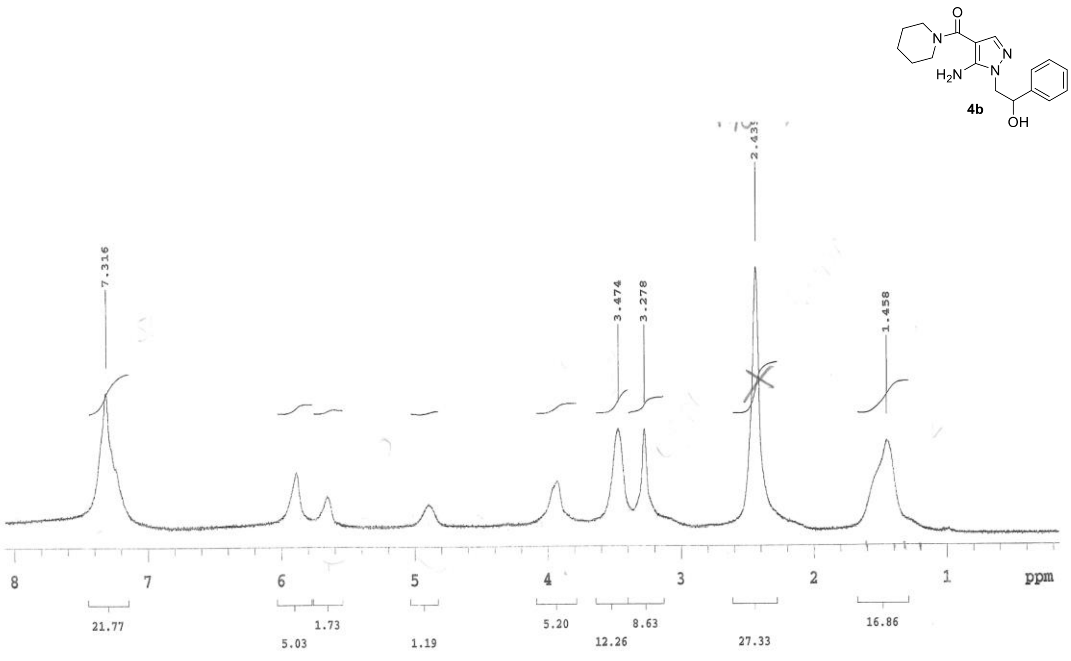

4b
7.316
3.474
3.278
2.435
1.458
8
7
6
5
4
3
2
1
ppm
21.77
5.03
1.73
1.19
5.20
12.26
8.63
27.33
16.86

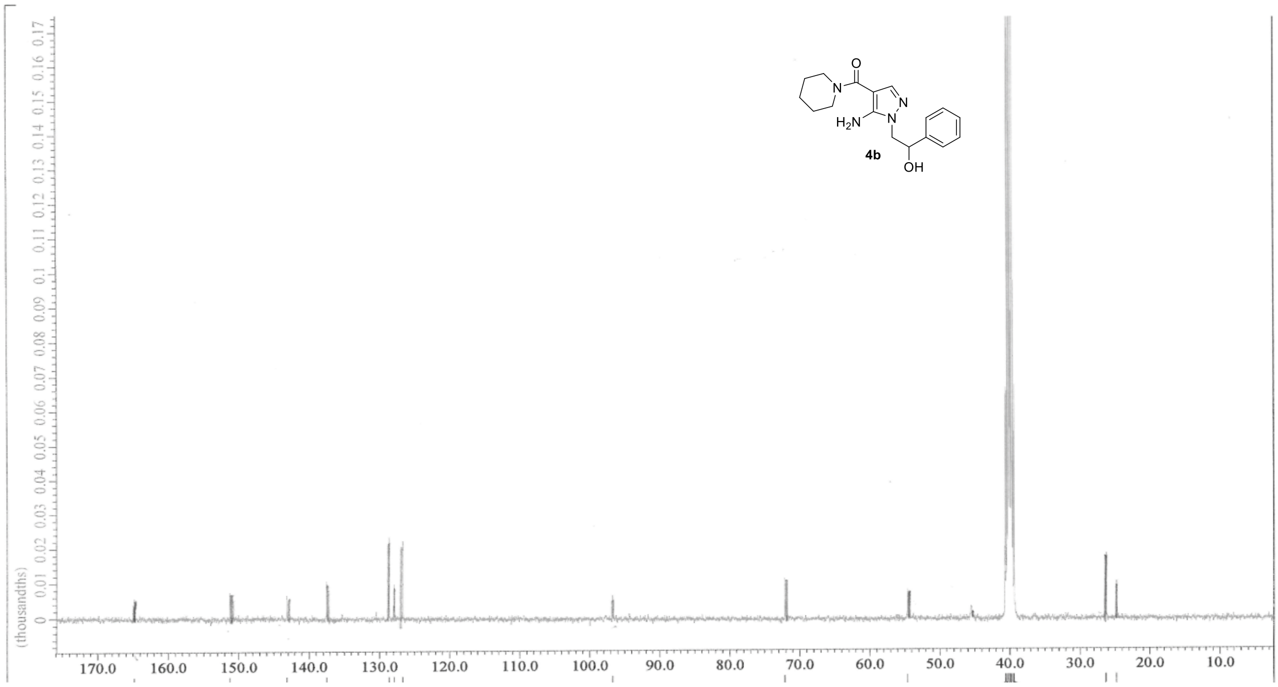

4b
H2N
OH
(thousandths)
170.0 160.0 150.0 140.0 130.0 120.0 110.0 100.0 90.0 80.0 70.0 60.0 50.0 40.0 30.0 20.0 10.0

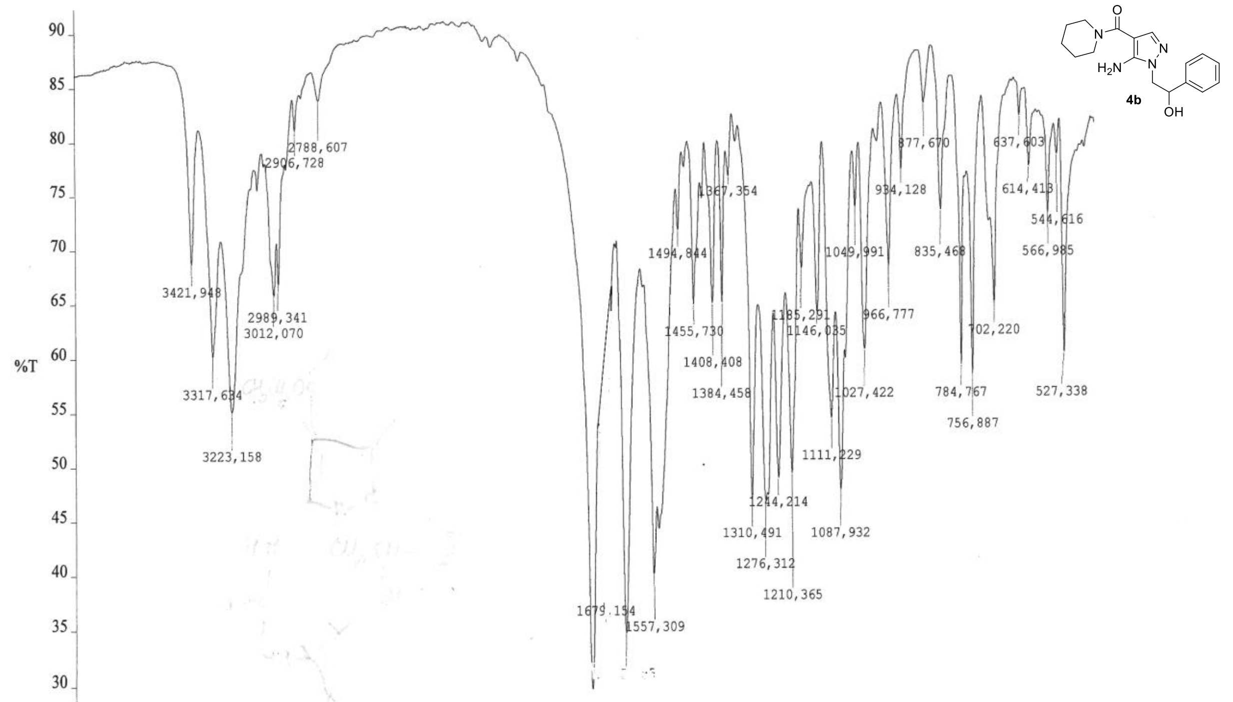

4b
%T
3421,948
3317,634
3223,158
3012,070
2989,341
2906,728
2788,607
1679,154
1557,309
1494,844
1455,730
1408,408
1384,458
1367,354
1310,491
1276,312
1244,214
1210,365
1185,291
1146,035
1111,229
1087,932
1049,991
1027,422
966,777
934,128
877,670
835,468
784,767
756,887
702,220
637,603
614,413
566,985
544,616
527,338

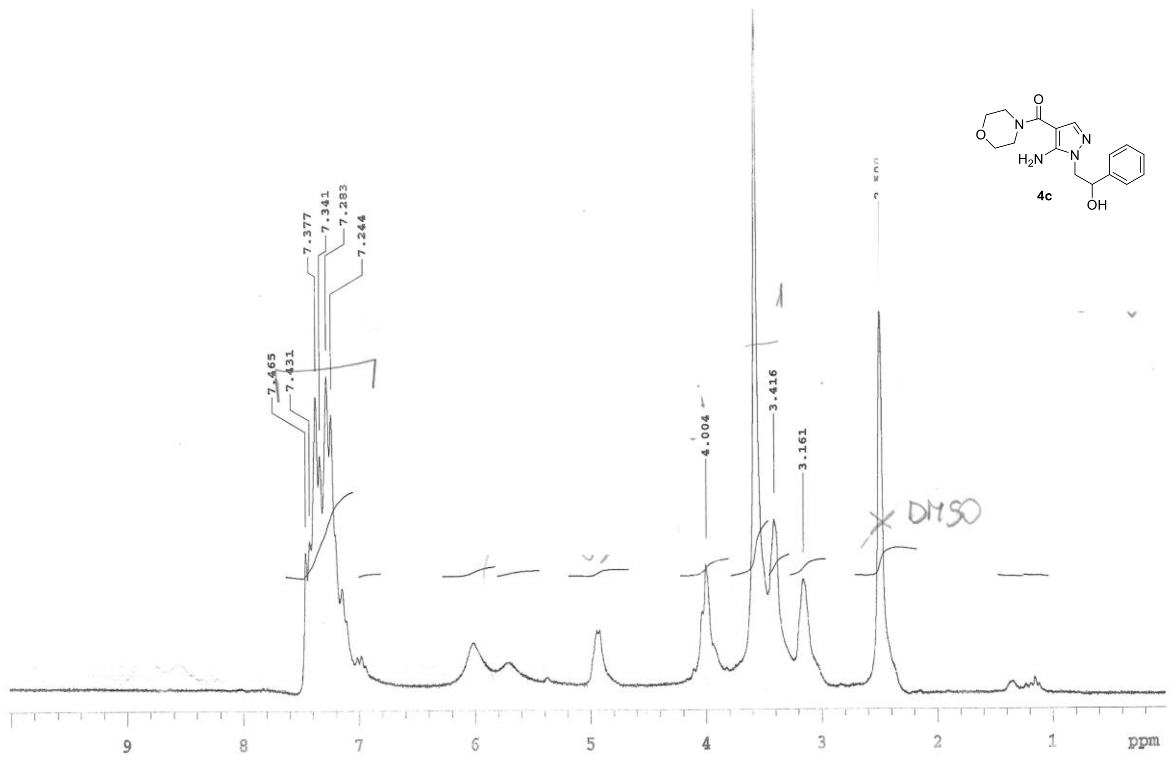
7.465
7.431
7.377
7.341
7.283
7.244
4.004
3.416
3.161
DMSO
4c
9 8 7 6 5 4 3 2 1 ppm

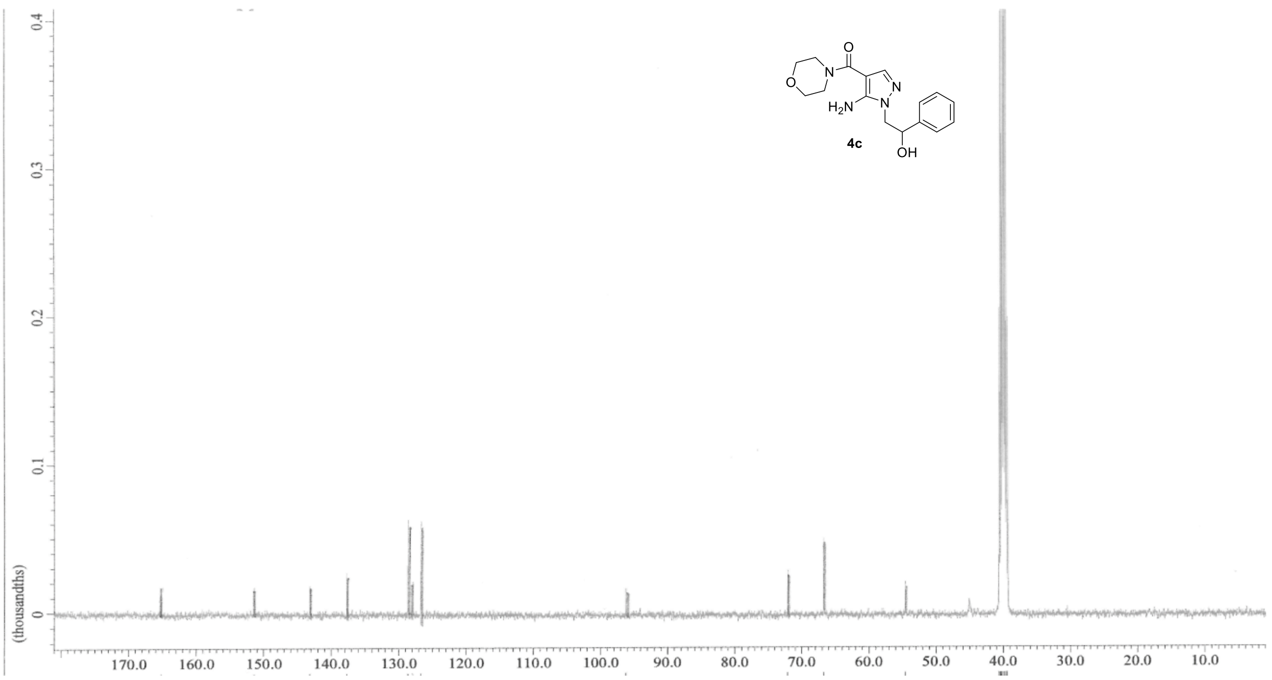
4c
(thousandths)
0 0.1 0.2 0.3 0.4
170.0 160.0 150.0 140.0 130.0 120.0 110.0 100.0 90.0 80.0 70.0 60.0 50.0 40.0 30.0 20.0 10.0

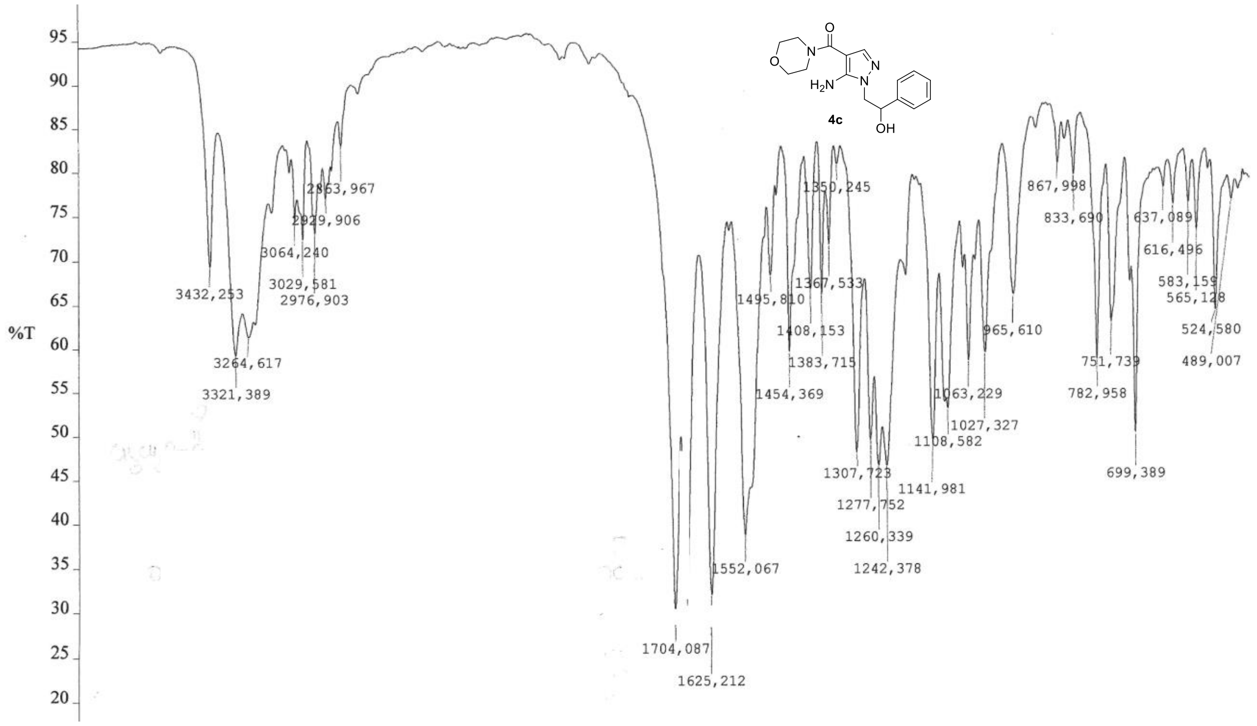
4c
OH
H2N
%T
95
90
85
80
75
70
65
60
55
50
45
40
35
30
25
20
3432,253
3321,389
3264,617
3064,240
3029,581
2976,903
2929,906
2863,967
1704,087
1625,212
1552,067
1495,810
1454,369
1408,153
1383,715
1367,533
1350,245
1307,723
1277,752
1260,339
1242,378
1141,981
1108,582
1063,229
1027,327
965,610
867,998
833,690
782,958
751,739
699,389
637,089
616,496
583,159
565,128
524,580
489,007

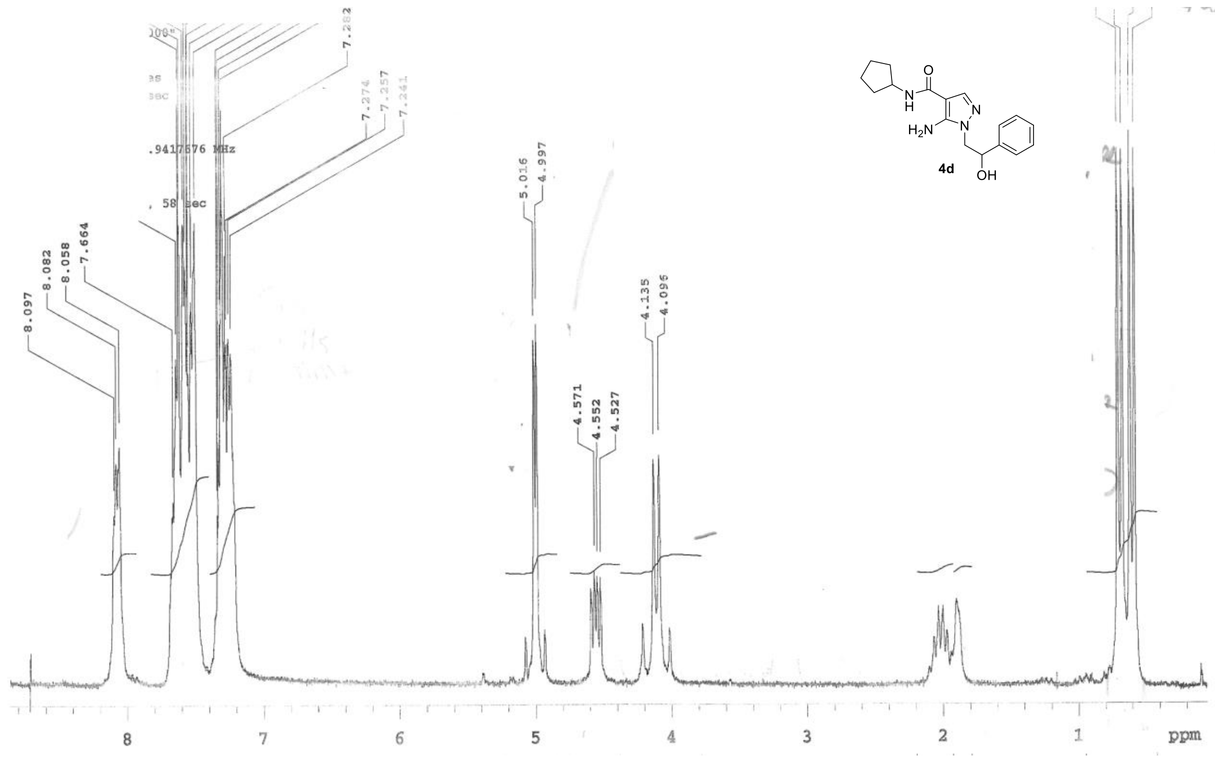
4d
OH
H2N
8.097
8.082
8.058
7.664
7.282
7.274
7.257
7.241
5.016
4.997
4.571
4.552
4.527
4.135
4.096
8
7
6
5
4
3
2
1
ppm

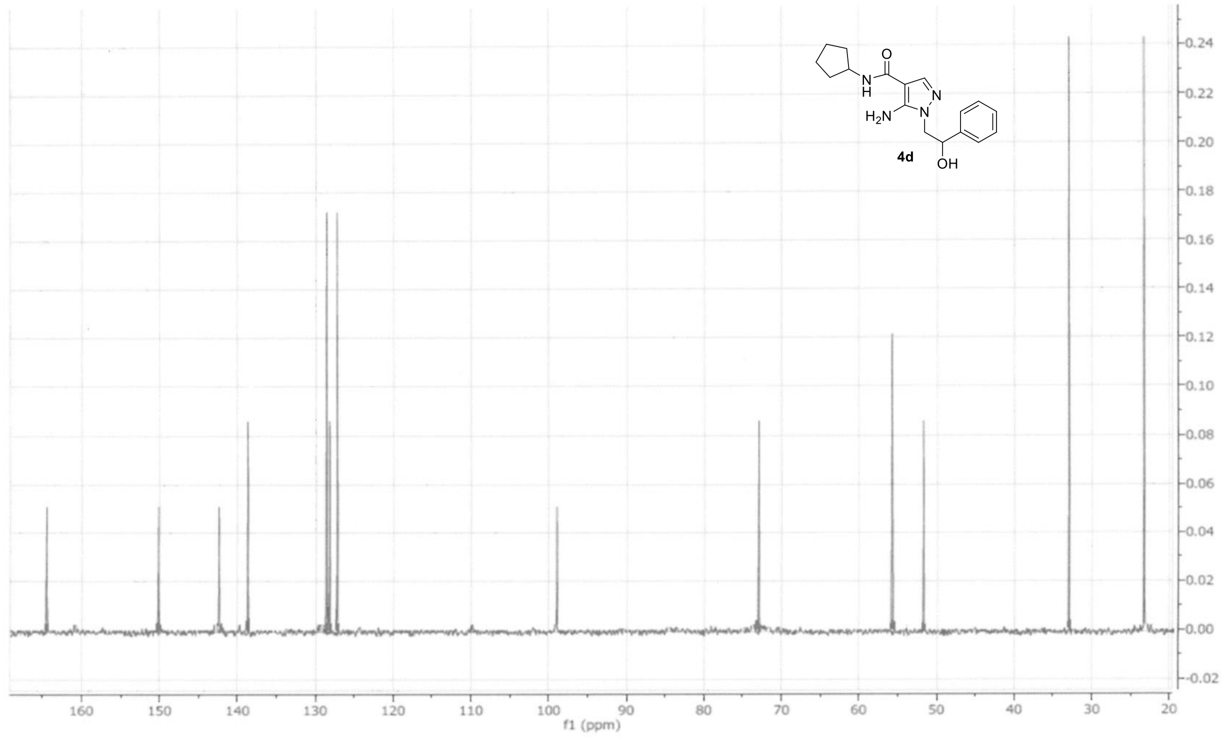

4d
NH
O
N
N
H2N
OH
0.24
0.22
0.20
0.18
0.16
0.14
0.12
0.10
0.08
0.06
0.04
0.02
0.00
-0.02
160
150
140
130
120
110
100
90
80
70
60
50
40
30
20
f1 (ppm)

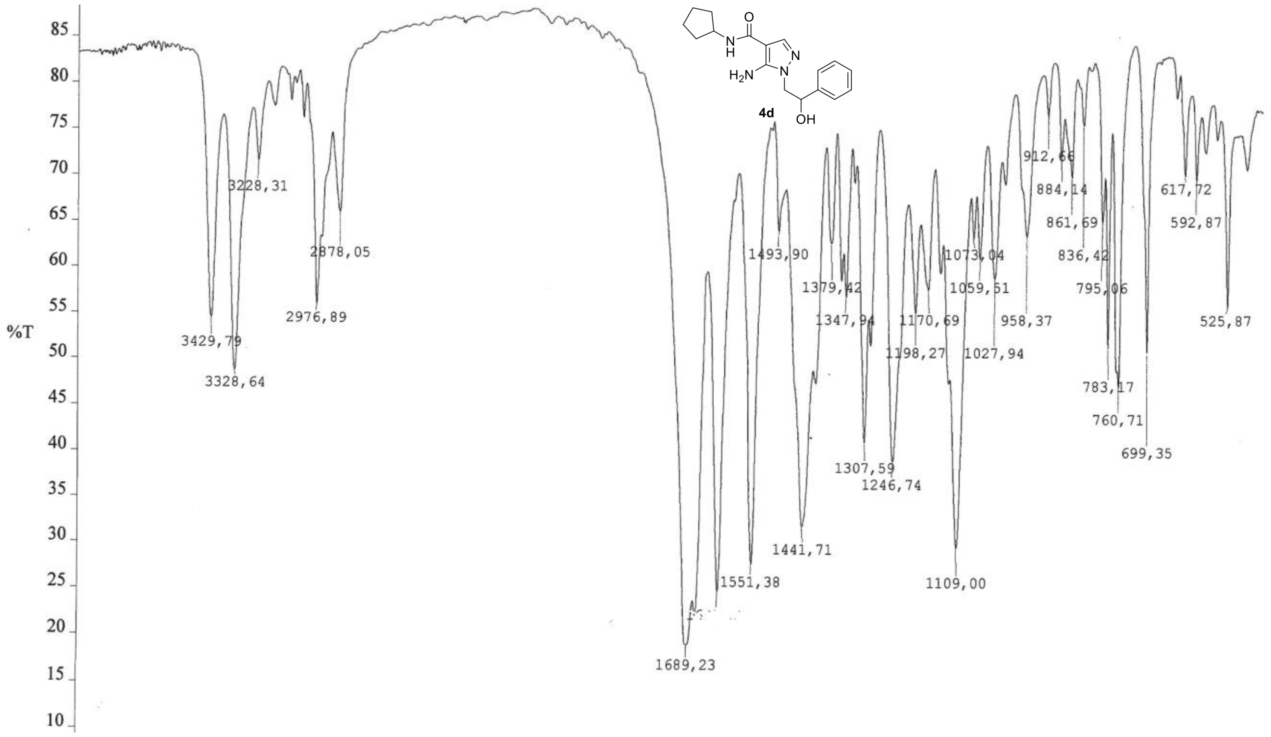

4d
%T
85
80
75
70
65
60
55
50
45
40
35
30
25
20
15
10
3228,31
2878,05
3429,79
3328,64
2976,89
1689,23
1551,38
1441,71
1493,90
1379,42
1347,94
1307,59
1246,74
1198,27
1170,69
1109,00
1073,04
1059,51
1027,94
958,37
912,66
884,14
861,69
836,42
795,06
783,17
760,71
699,35
617,72
592,87
525,87

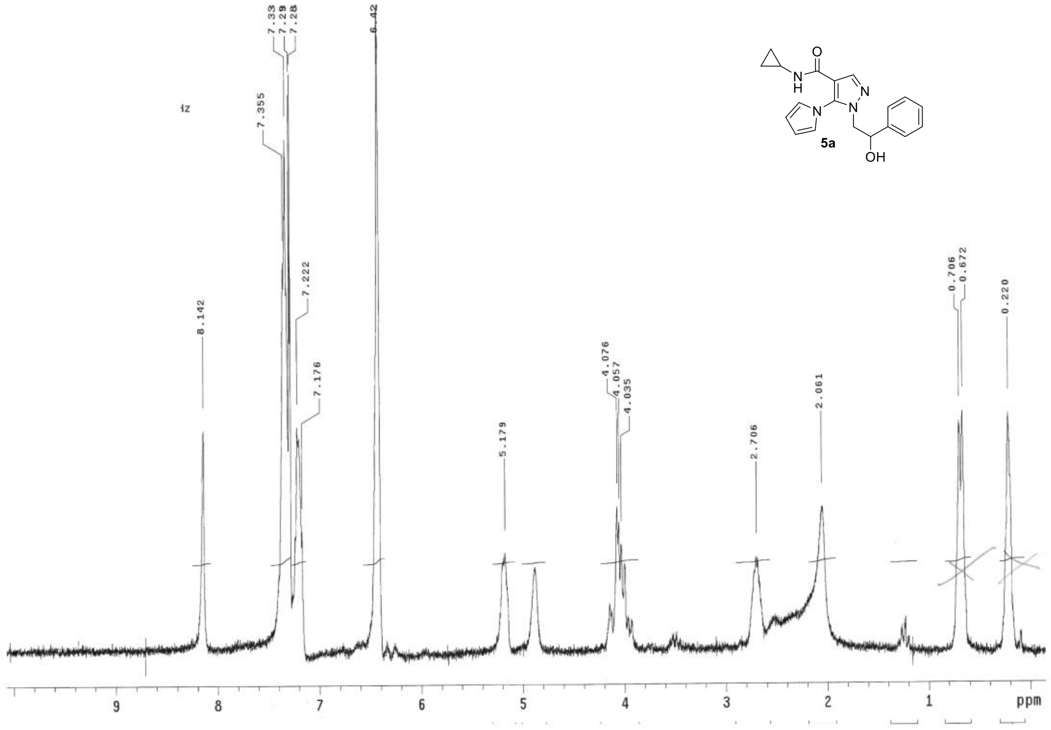

8.142
7.355
7.33
7.29
7.28
7.222
7.176
6.42
5.179
4.076
4.057
4.035
2.706
2.061
0.706
0.672
0.220
9
8
7
6
5
4
3
2
1
ppm
5a

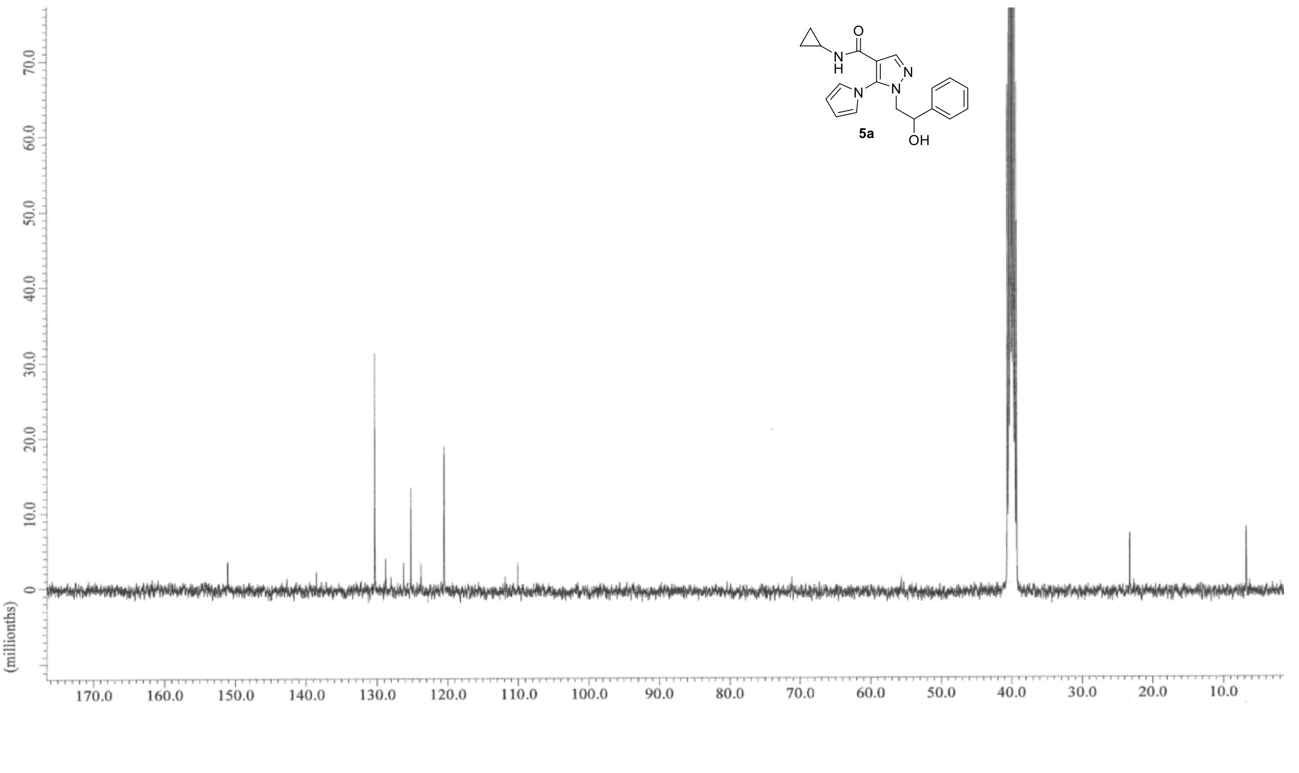

70.0
60.0
50.0
40.0
30.0
20.0
10.0
0
(millionths)
170.0
160.0
150.0
140.0
130.0
120.0
110.0
100.0
90.0
80.0
70.0
60.0
50.0
40.0
30.0
20.0
10.0
5a

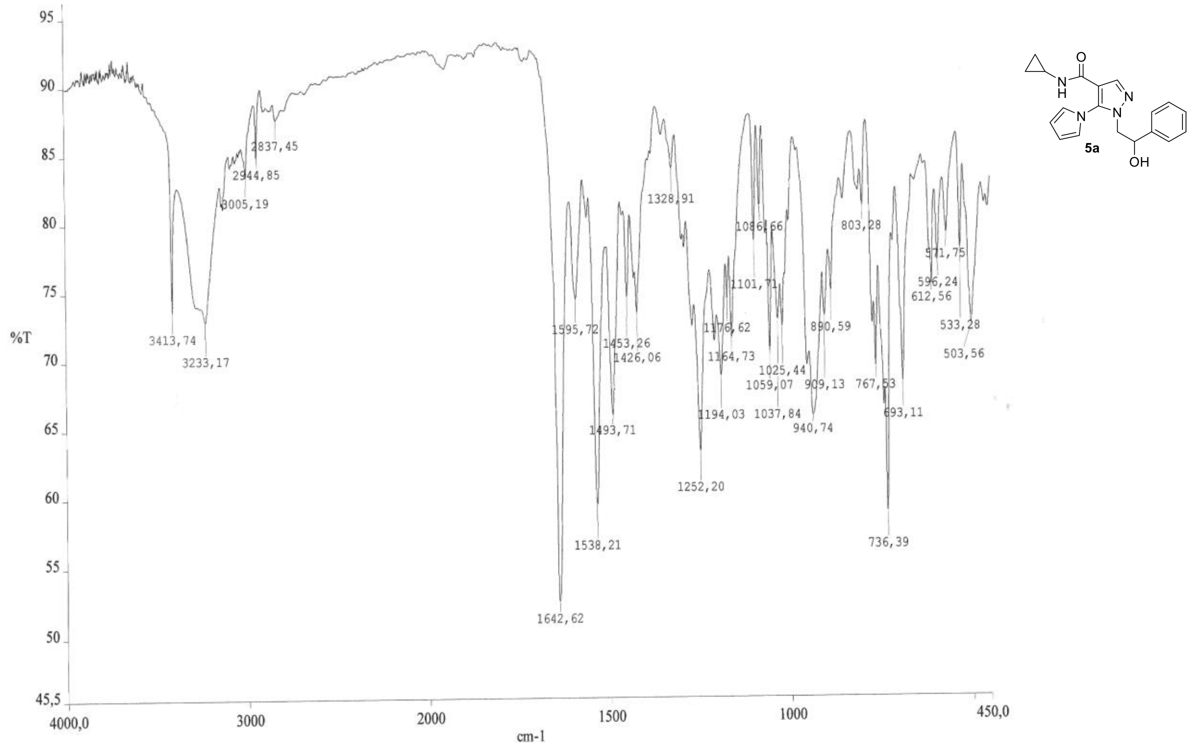
95
90
85
80
75
70
65
60
55
50
45,5
%T
4000,0
3000
2000
1500
1000
450,0
cm-1
3413,74
3233,17
3005,19
2944,85
2837,45
1642,62
1595,72
1538,21
1493,71
1453,26
1426,06
1328,91
1252,20
1194,03
1176,62
1164,73
1101,71
1086,66
1059,07
1037,84
1025,44
940,74
909,13
890,59
803,28
767,53
736,39
693,11
612,56
596,24
571,75
533,28
503,56
O
N
H
N
N
N
OH
5a

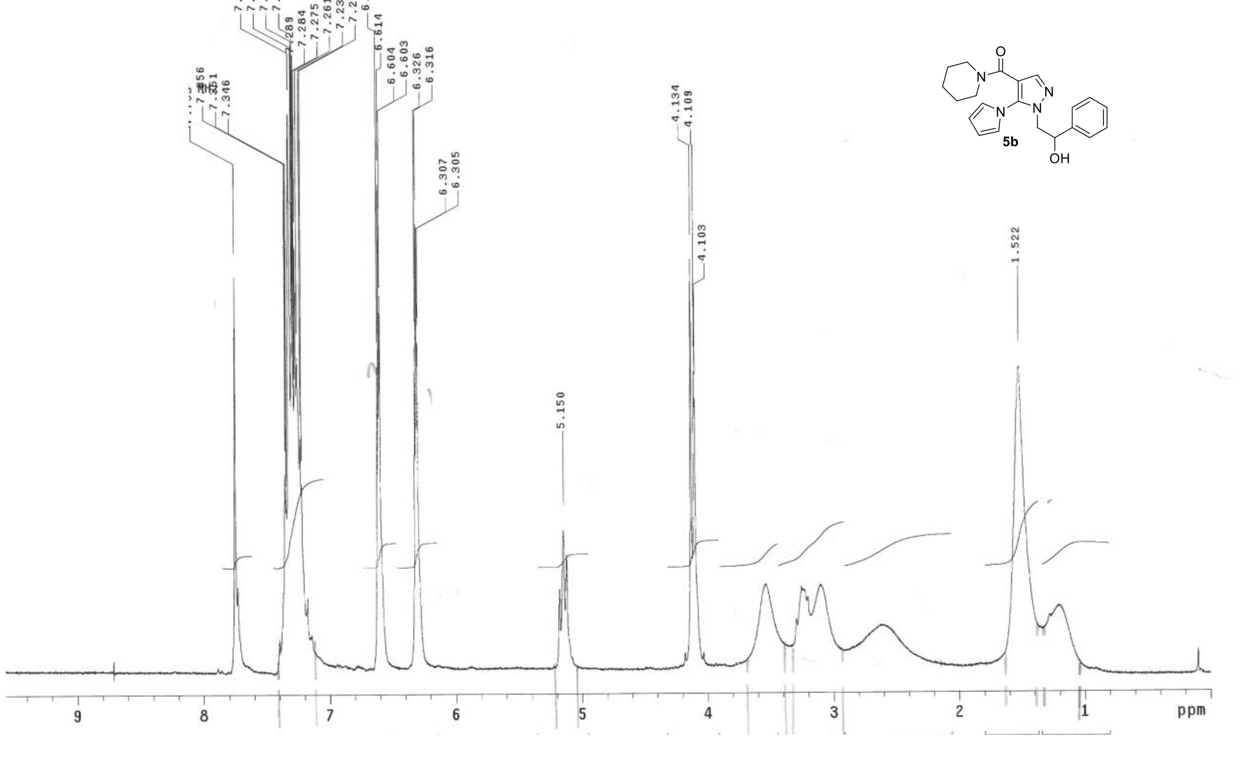
7.356
7.351
7.346
7.32
7.30
7.29
7.29
7.289
7.284
7.275
7.261
7.237
7.227
6.62
6.614
6.604
6.603
6.326
6.316
6.307
6.305
5.150
4.134
4.109
4.103
1.522
9
8
7
6
5
4
3
2
1
ppm
O
N
N
N
N
OH
5b

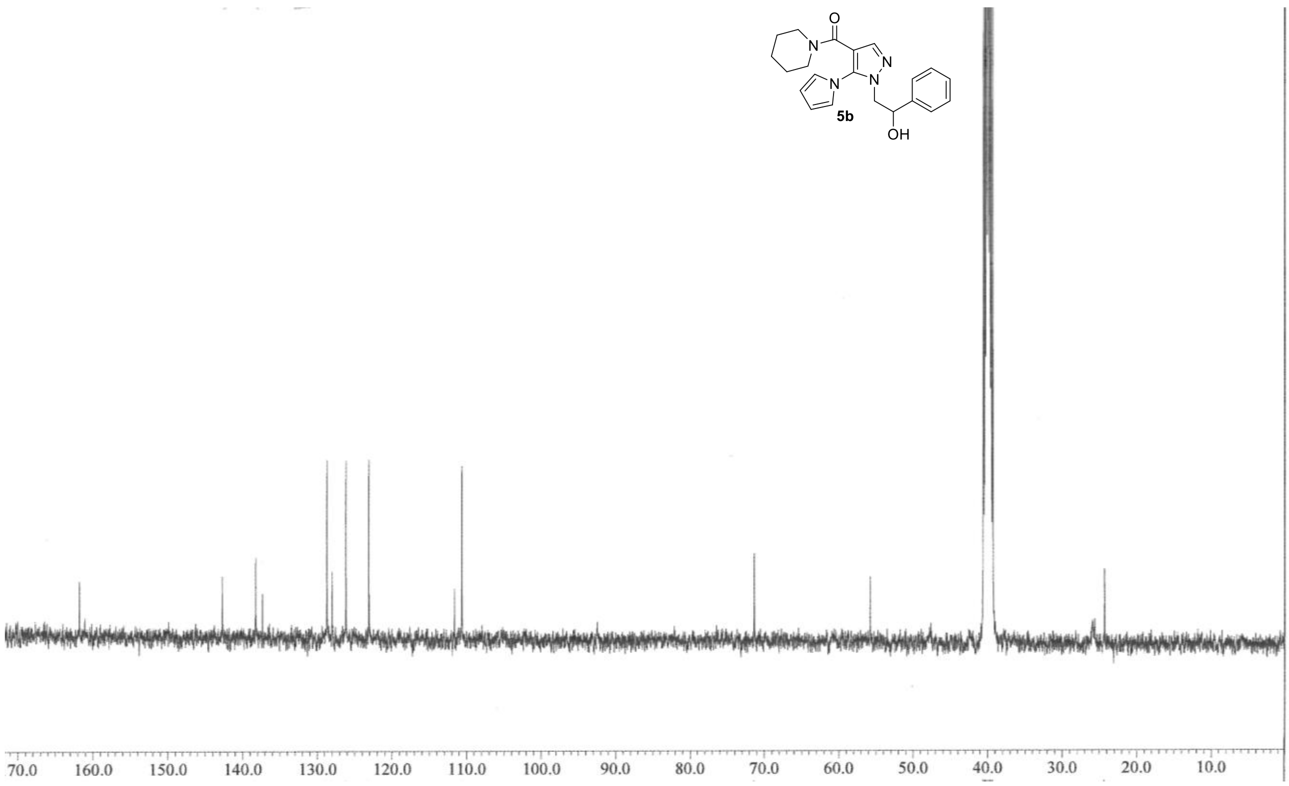
5b
OH
70.0 160.0 150.0 140.0 130.0 120.0 110.0 100.0 90.0 80.0 70.0 60.0 50.0 40.0 30.0 20.0 10.0

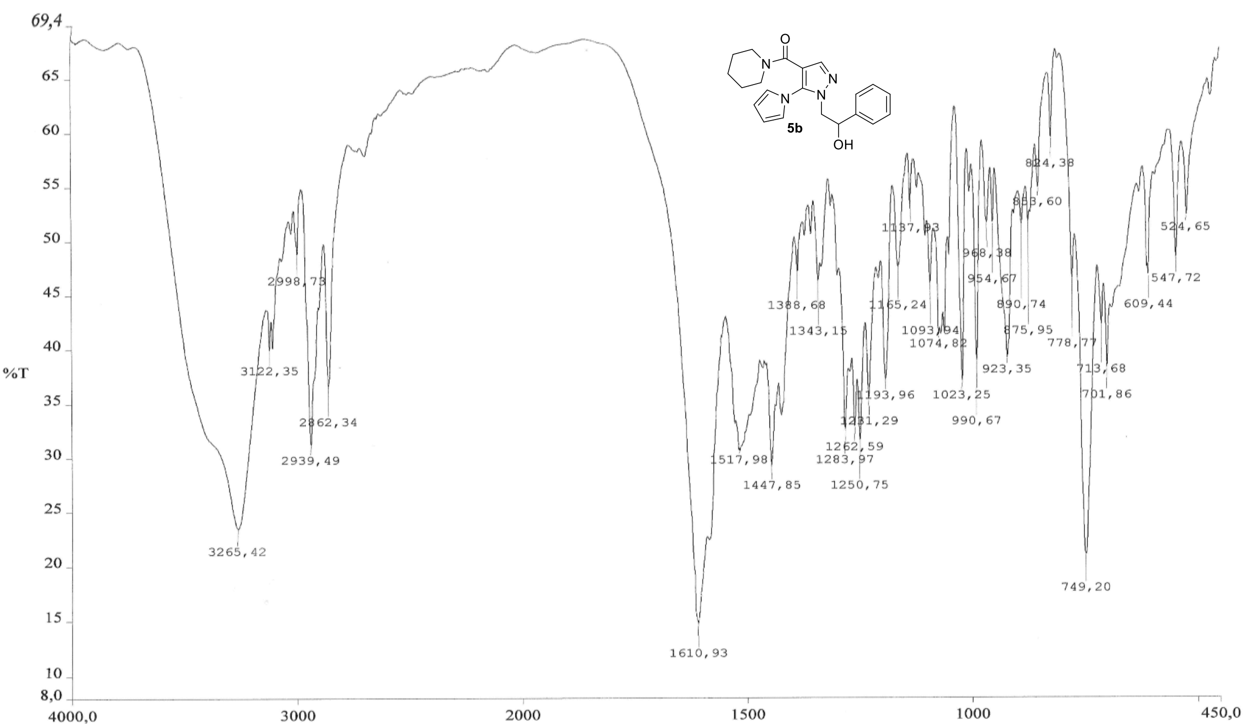
5b
OH
69,4
65
60
55
50
45
40
%T
35
30
25
20
15
10
8,0
4000,0
3000
2000
1500
1000
450,0
3265,42
3122,35
2998,73
2939,49
2862,34
1610,93
1517,98
1447,85
1388,68
1343,15
1283,97
1262,59
1250,75
1231,29
1193,96
1165,24
1137,93
1093,94
1074,82
1023,25
990,67
968,38
954,67
923,35
890,74
875,95
853,60
824,38
778,77
749,20
713,68
701,86
609,44
547,72
524,65

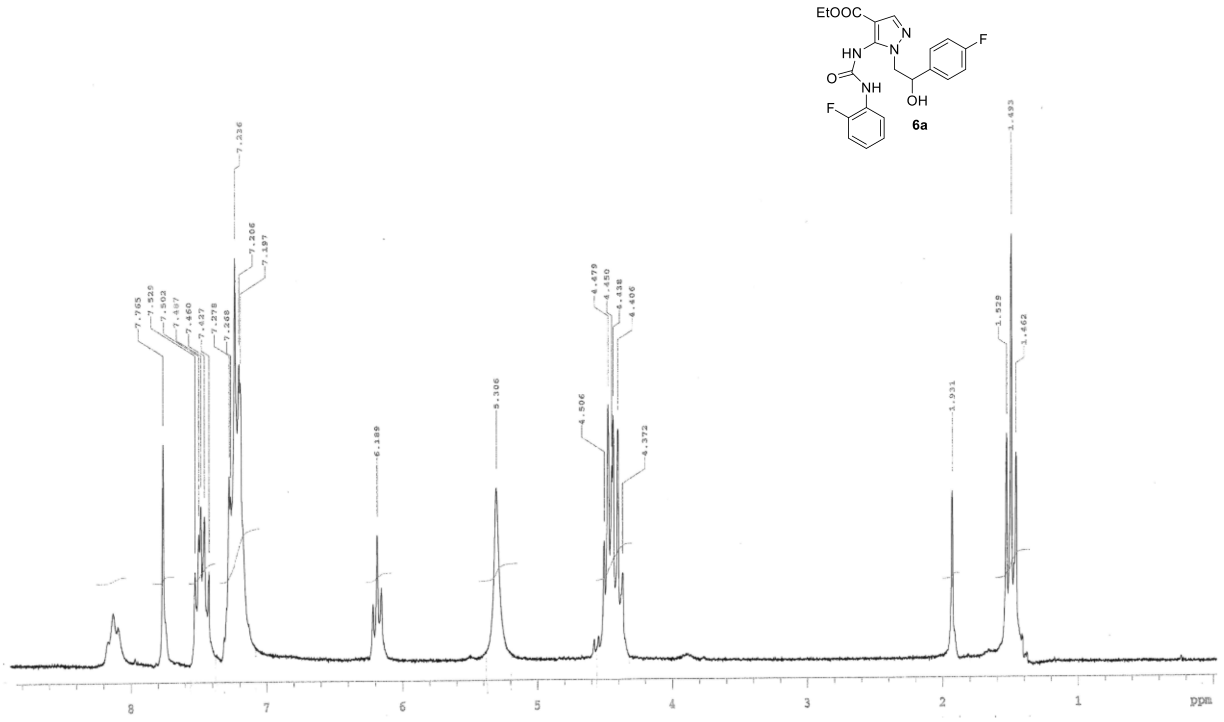
EtOOC
HN
N
N
F
O
NH
OH
F
6a
8
7
6
5
4
3
2
1
ppm

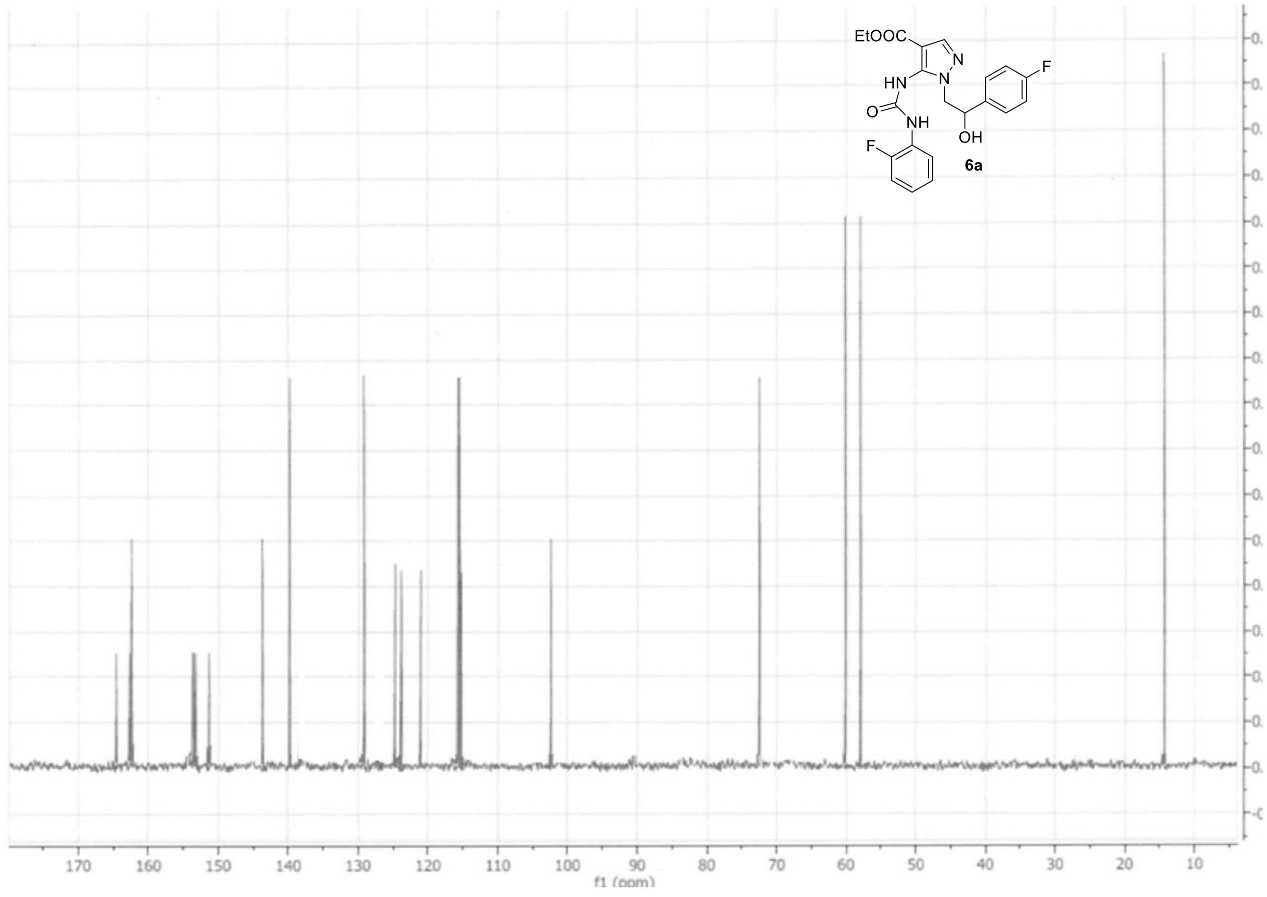
EtOOC
HN
N
N
F
O
NH
OH
F
6a
170
160
150
140
130
120
110
100
90
80
70
60
50
40
30
20
10
f1 (ppm)

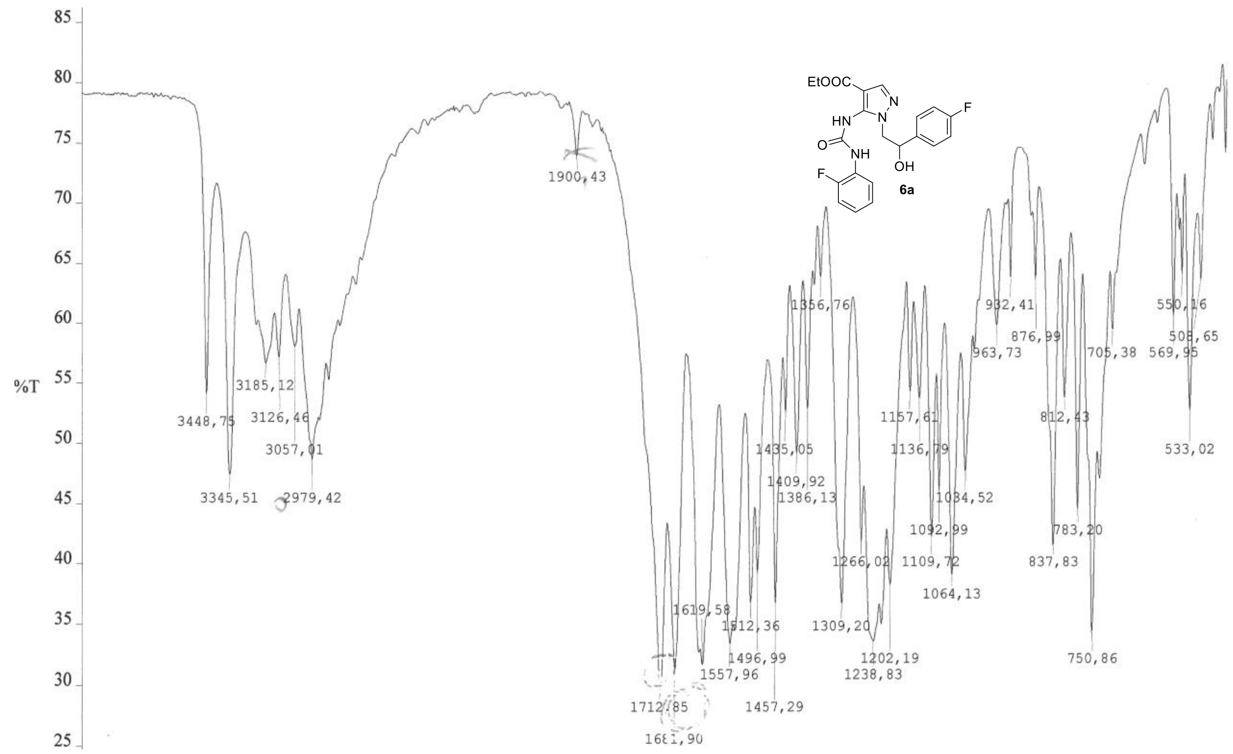

85
80
75
70
65
60
55
50
45
40
35
30
25
%T
EtOOC
N
N
HN
F
O
NH
OH
F
6a
3448,75
3345,51
3185,12
3126,46
3057,01
2979,42
1900,43
1712,85
1681,90
1619,58
1557,96
1512,36
1496,99
1457,29
1435,05
1409,92
1386,13
1356,76
1309,20
1266,02
1238,83
1202,19
1157,61
1136,79
1109,72
1092,99
1064,13
1034,52
963,73
932,41
876,99
837,83
812,43
783,20
750,86
705,38
569,95
550,16
533,02
508,65

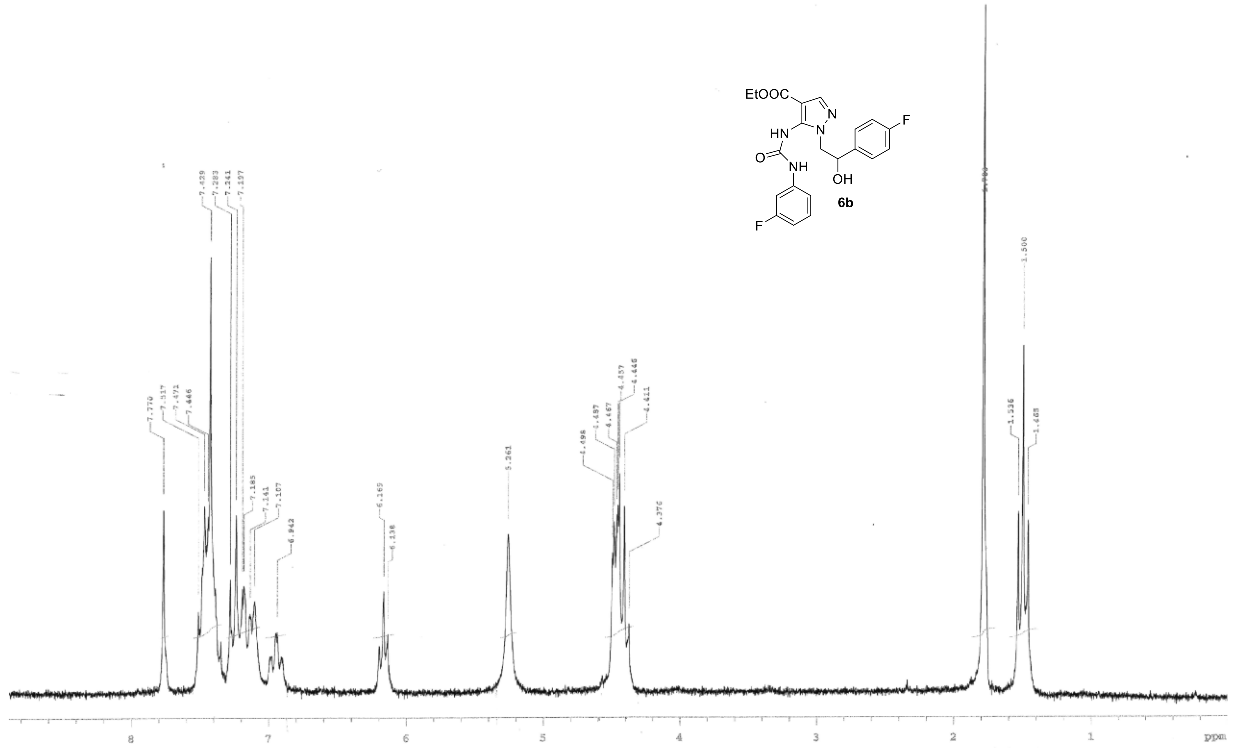

EtOOC
N
N
HN
F
O
NH
OH
F
6b
8
7
6
5
4
3
2
1
ppm

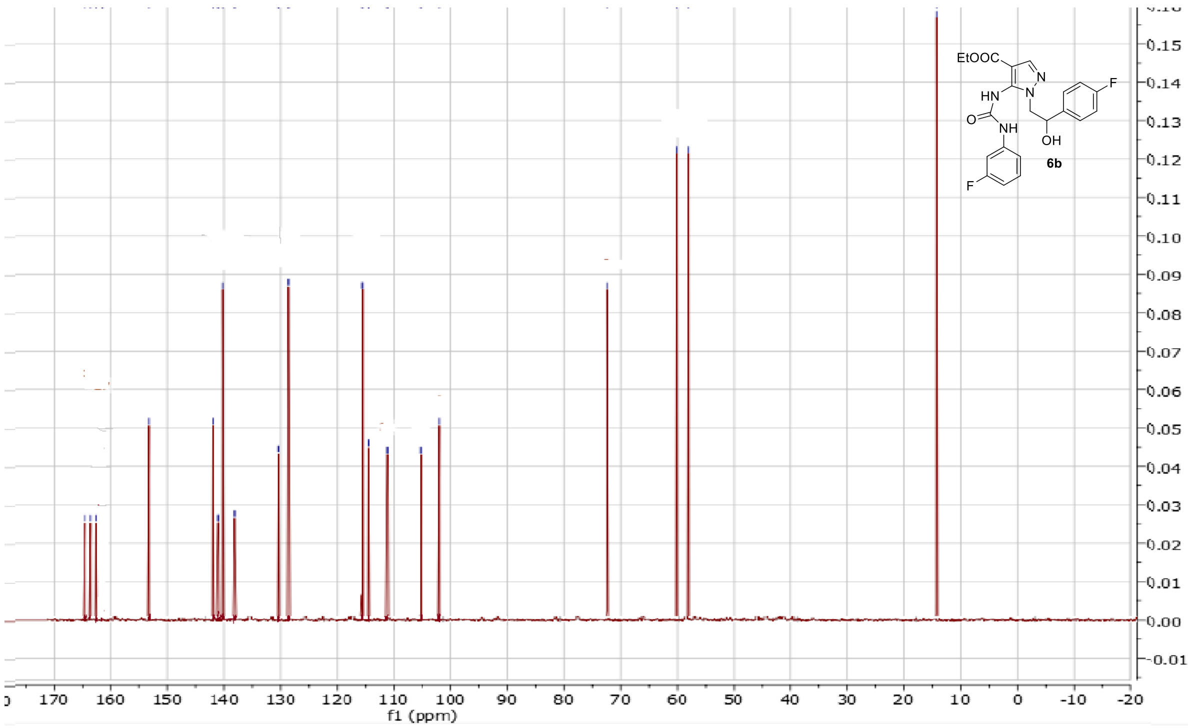
EtOOC
N
N
HN
O
NH
OH
F
F
6b
170 160 150 140 130 120 110 100 90 80 70 60 50 40 30 20 10 0 -10 -20
f1 (ppm)
0.15
0.14
0.13
0.12
0.11
0.10
0.09
0.08
0.07
0.06
0.05
0.04
0.03
0.02
0.01
0.00
-0.01

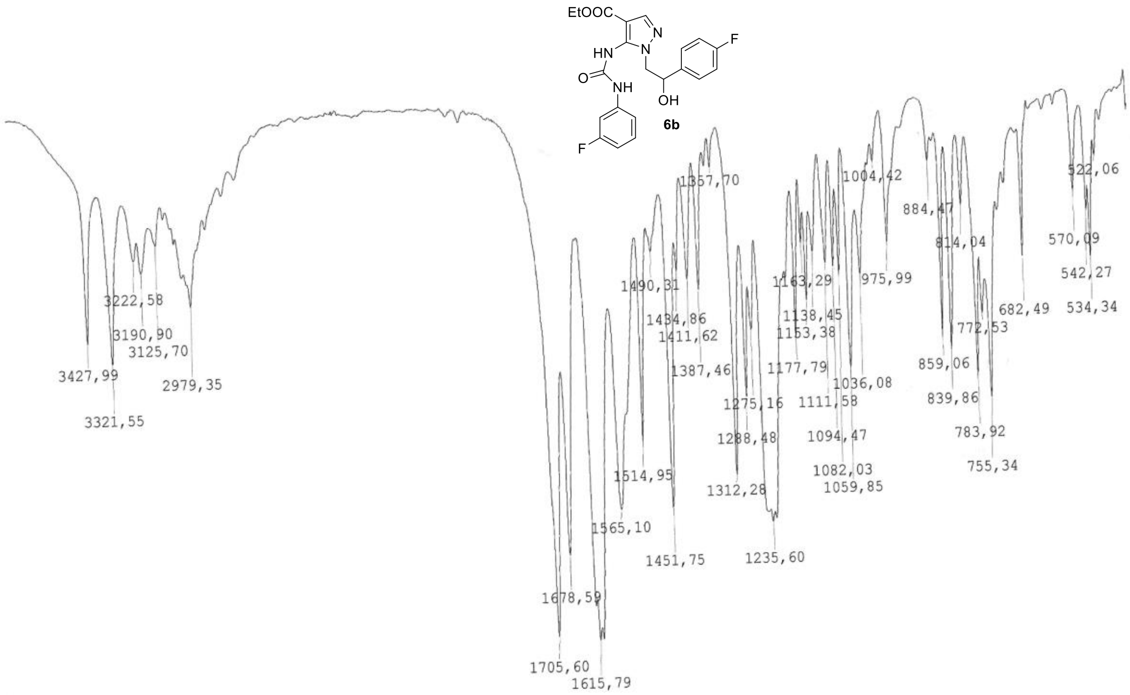
EtOOC
N
N
HN
O
NH
OH
F
F
6b
3427,99
3321,55
3222,58
3190,90
3125,70
2979,35
1705,60
1678,59
1615,79
1565,10
1514,95
1490,31
1451,75
1434,86
1411,62
1387,46
1357,70
1312,28
1288,48
1275,16
1235,60
1177,79
1163,29
1153,38
1138,45
1111,58
1094,47
1082,03
1059,85
1036,08
1004,42
975,99
884,47
859,06
839,86
814,04
783,92
772,53
755,34
682,49
570,09
542,27
534,34
522,06

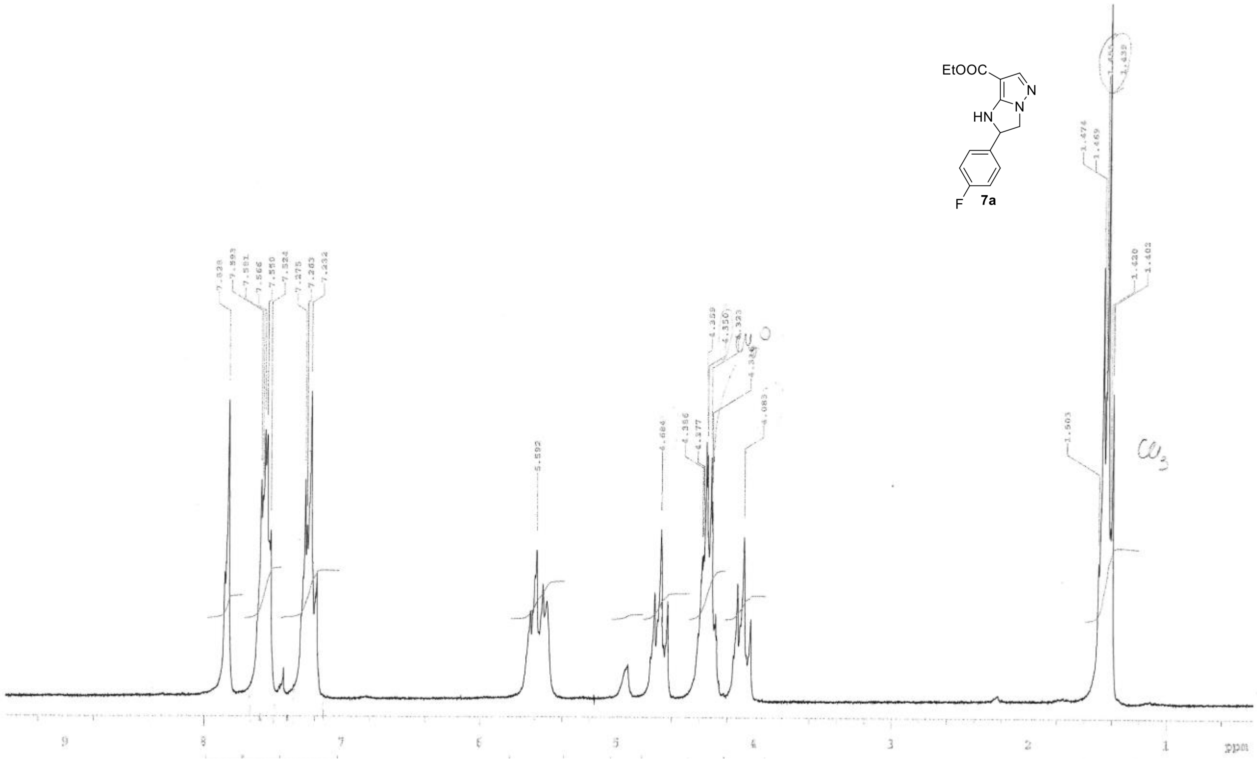
EtOOC
N
N
HN
F
7a
ppm

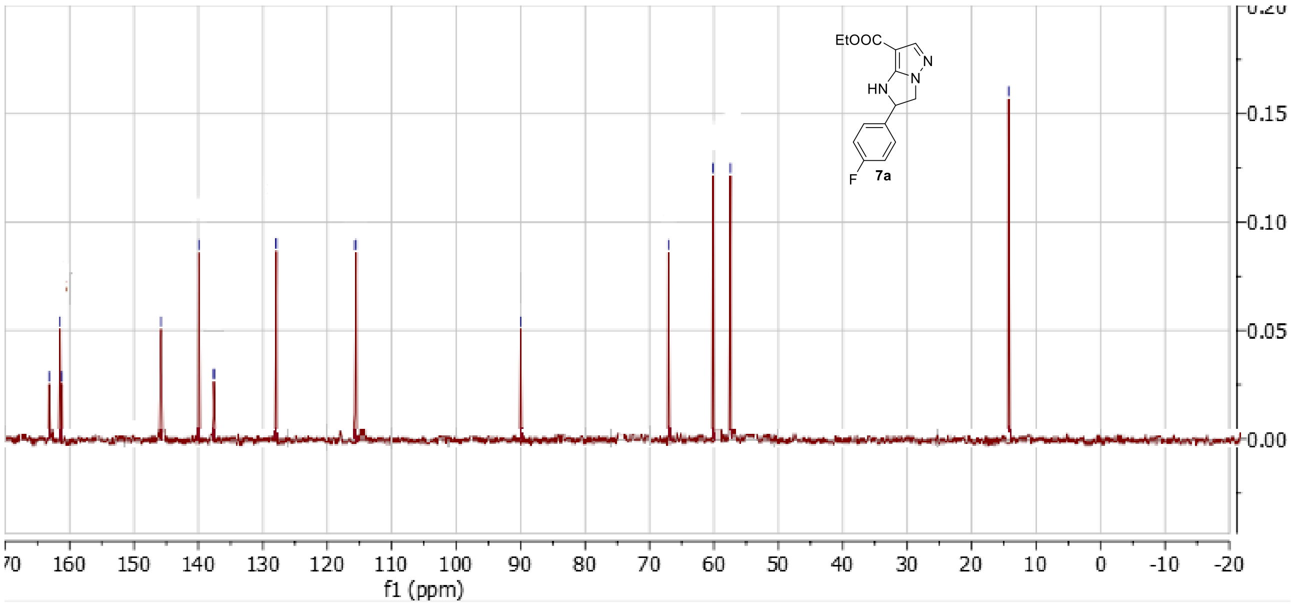
EtOOC
N
N
HN
F
7a
f1 (ppm)

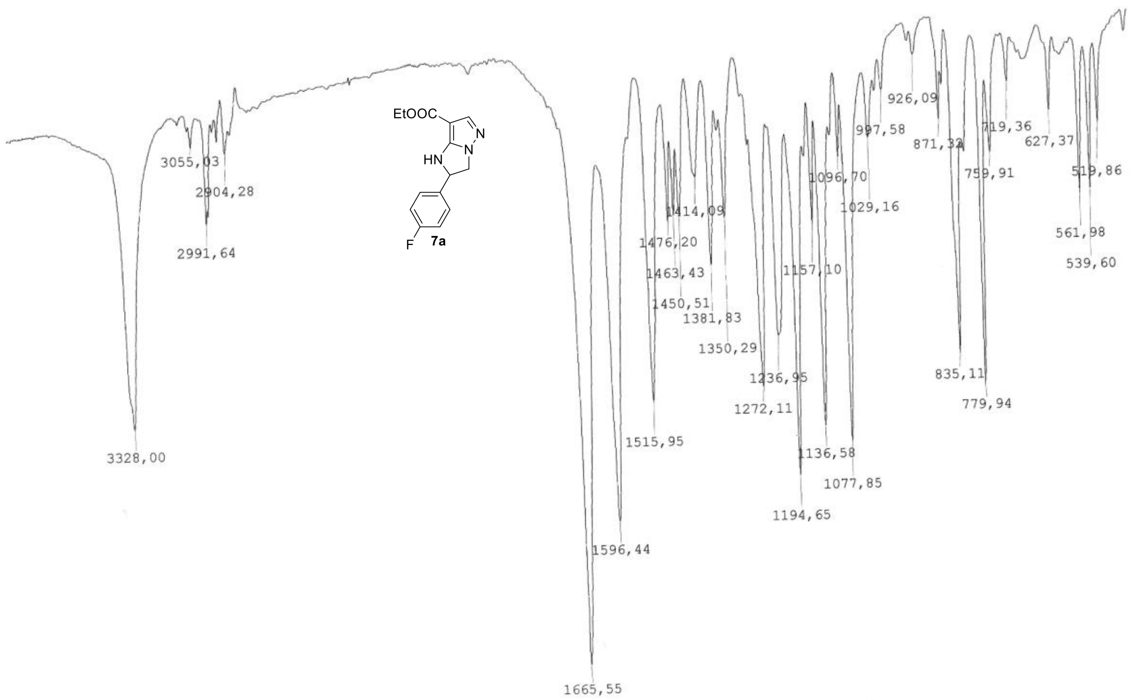
EtOOC
N
N
HN
F
7a
3328,00
3055,03
2904,28
2991,64
1665,55
1596,44
1515,95
1476,20
1463,43
1450,51
1414,09
1381,83
1350,29
1272,11
1236,95
1194,65
1157,10
1136,58
1096,70
1077,85
1029,16
997,58
926,09
871,32
835,11
779,94
759,91
719,36
627,37
561,98
539,60
519,86

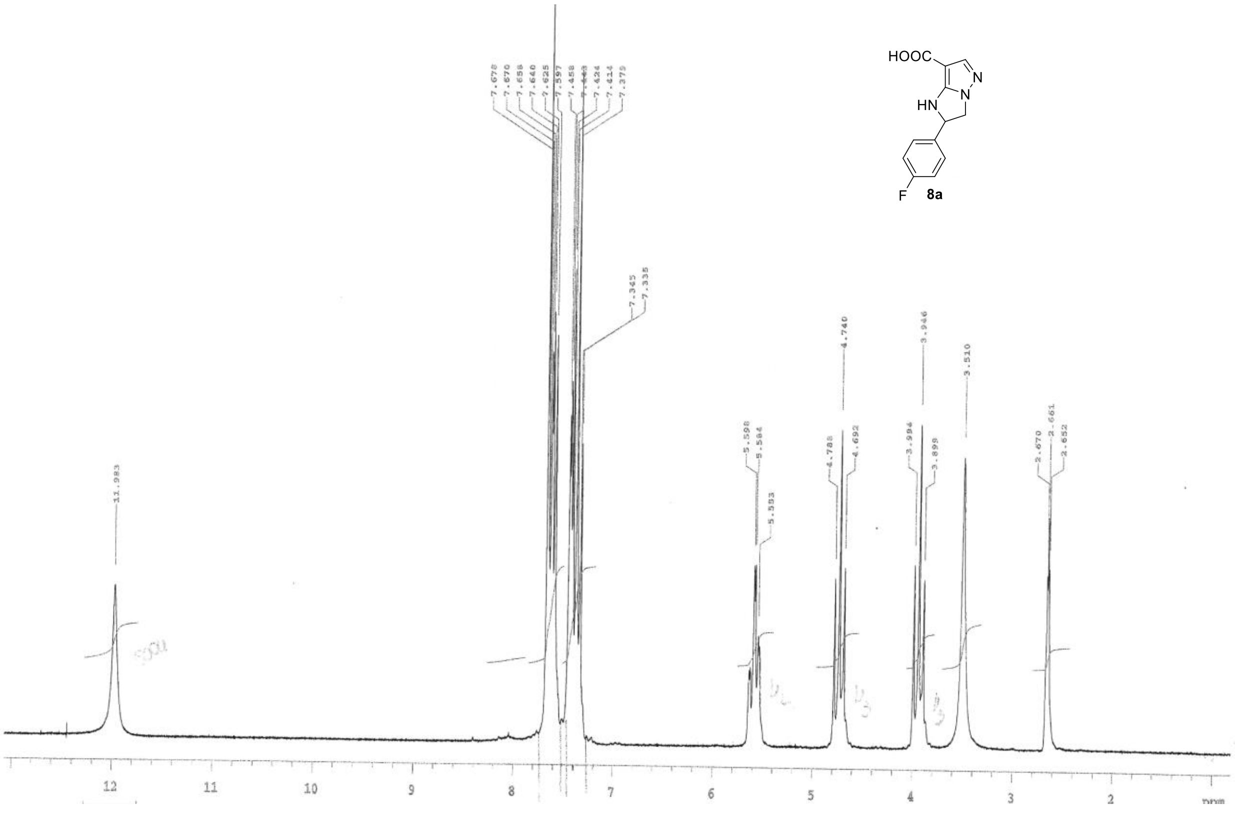
HOOC
N
N
HN
F
8a
11.993
7.678
7.670
7.658
7.640
7.625
7.597
7.458
7.443
7.434
7.414
7.379
7.345
7.335
5.598
5.584
5.553
4.788
4.740
4.692
3.994
3.946
3.899
3.510
2.670
2.661
2.652
12
11
10
9
8
7
6
5
4
3
2
ppm

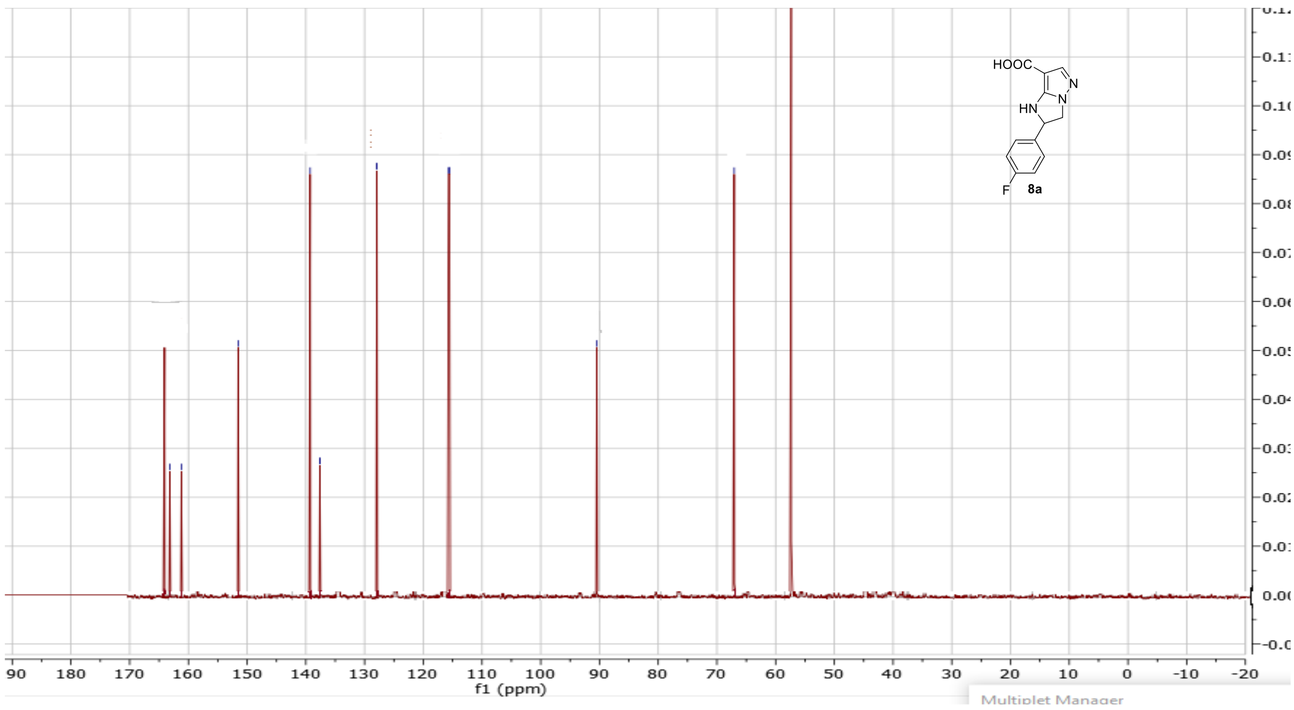
HOOC
N
N
HN
F
8a
90 180 170 160 150 140 130 120 110 100 90 80 70 60 50 40 30 20 10 0 -10 -20
f1 (ppm)
Multiplet Manager

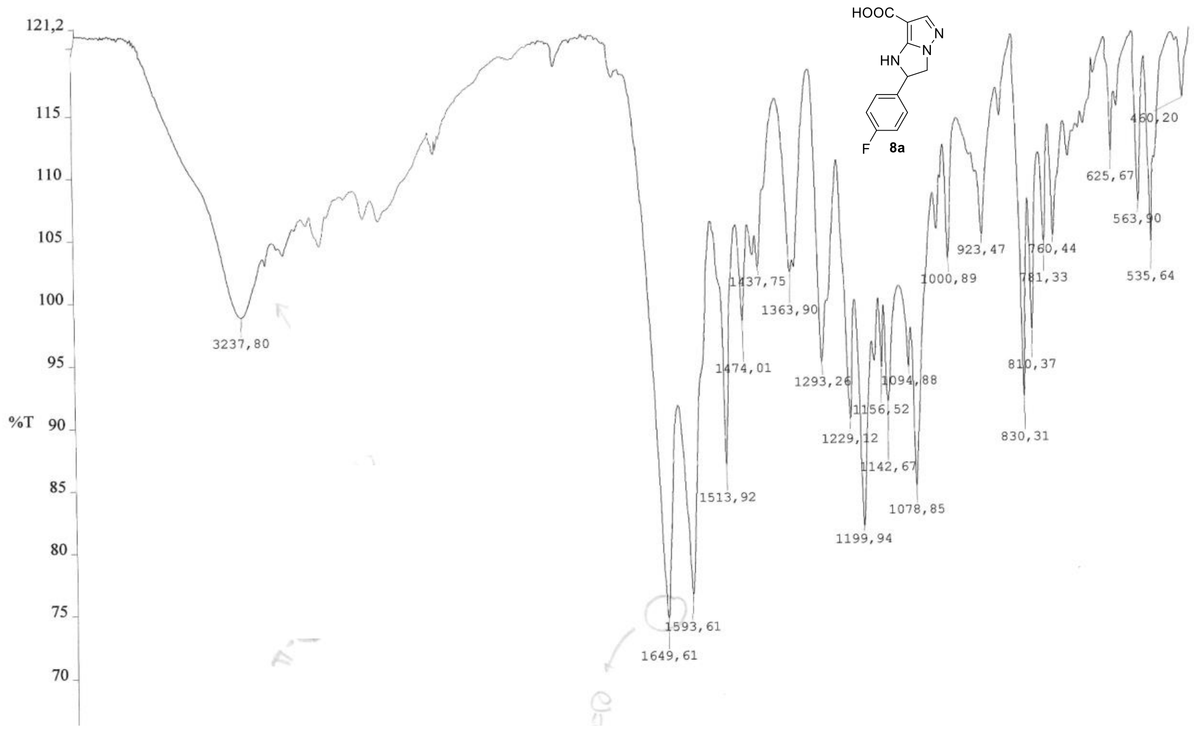
HOOC
N
N
HN
F
8a
%T
121,2
115
110
105
100
95
90
85
80
75
70
3237,80
1649,61
1593,61
1513,92
1474,01
1437,75
1363,90
1293,26
1229,12
1199,94
1156,52
1142,67
1094,88
1078,85
1000,89
923,47
830,31
810,37
781,33
760,44
625,67
563,90
535,64
460,20

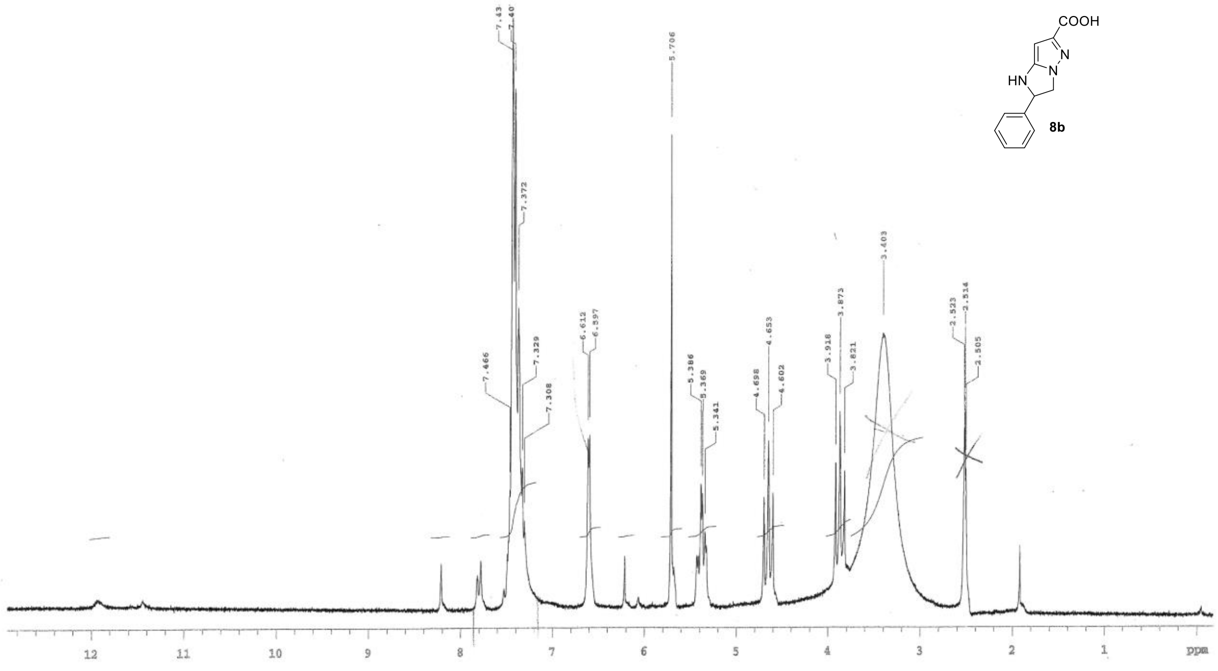
COOH
N
N
HN
8b
7.43
7.40
7.372
7.466
7.329
7.308
6.612
6.597
5.706
5.386
5.369
5.341
4.698
4.653
4.602
3.916
3.873
3.821
3.403
2.523
2.514
2.505
12
11
10
9
8
7
6
5
4
3
2
1
ppm

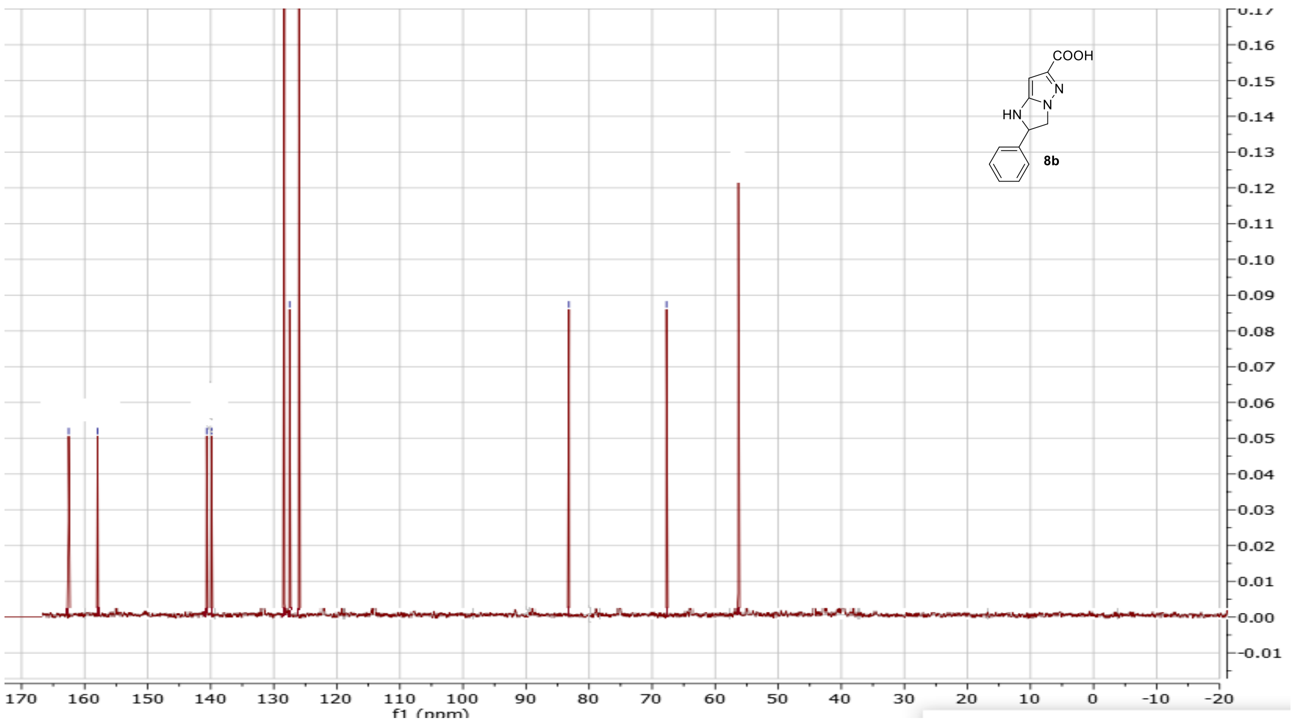
COOH
N
N
HN
8b
0.16
0.15
0.14
0.13
0.12
0.11
0.10
0.09
0.08
0.07
0.06
0.05
0.04
0.03
0.02
0.01
0.00
-0.01
170
160
150
140
130
120
110
100
90
80
70
60
50
40
30
20
10
0
-10
-20
f1 (ppm)

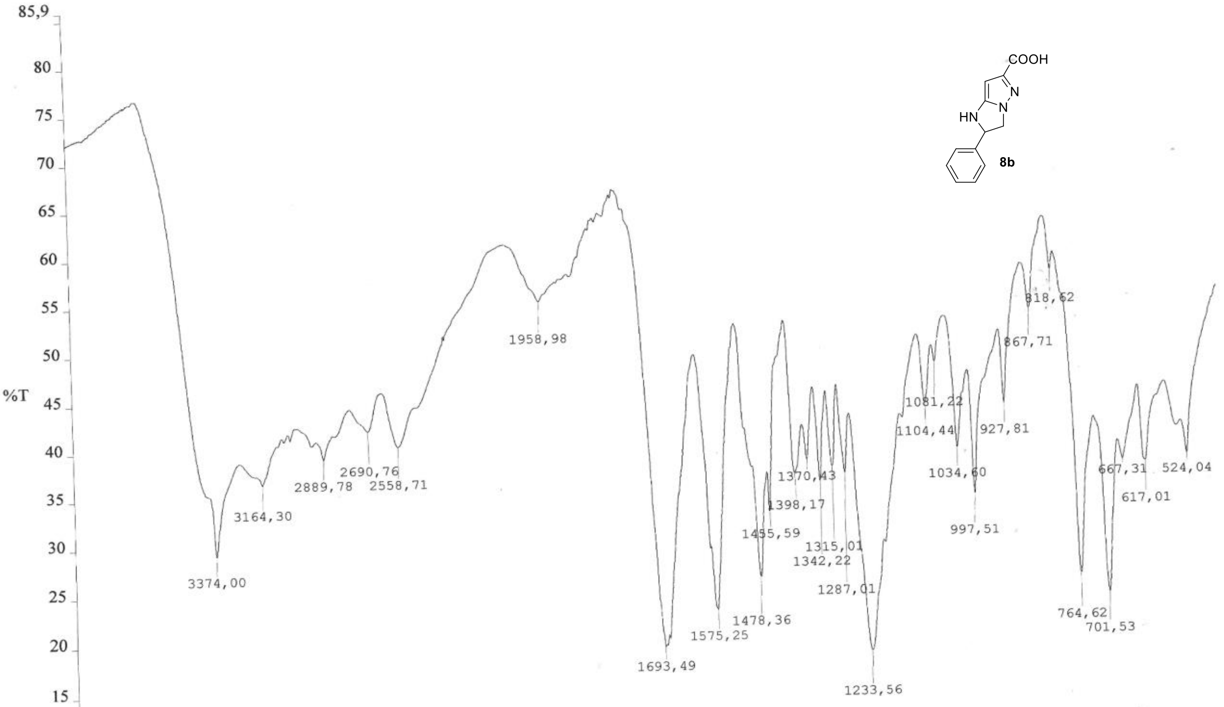
85,9
80
75
70
65
60
55
50
%T
45
40
35
30
25
20
15
COOH
HN
8b
3374,00
3164,30
2889,78
2690,76
2558,71
1958,98
1693,49
1575,25
1478,36
1455,59
1398,17
1370,43
1342,22
1315,01
1287,01
1233,56
1104,44
1081,22
1034,60
997,51
927,81
867,71
818,62
764,62
701,53
667,31
617,01
524,04

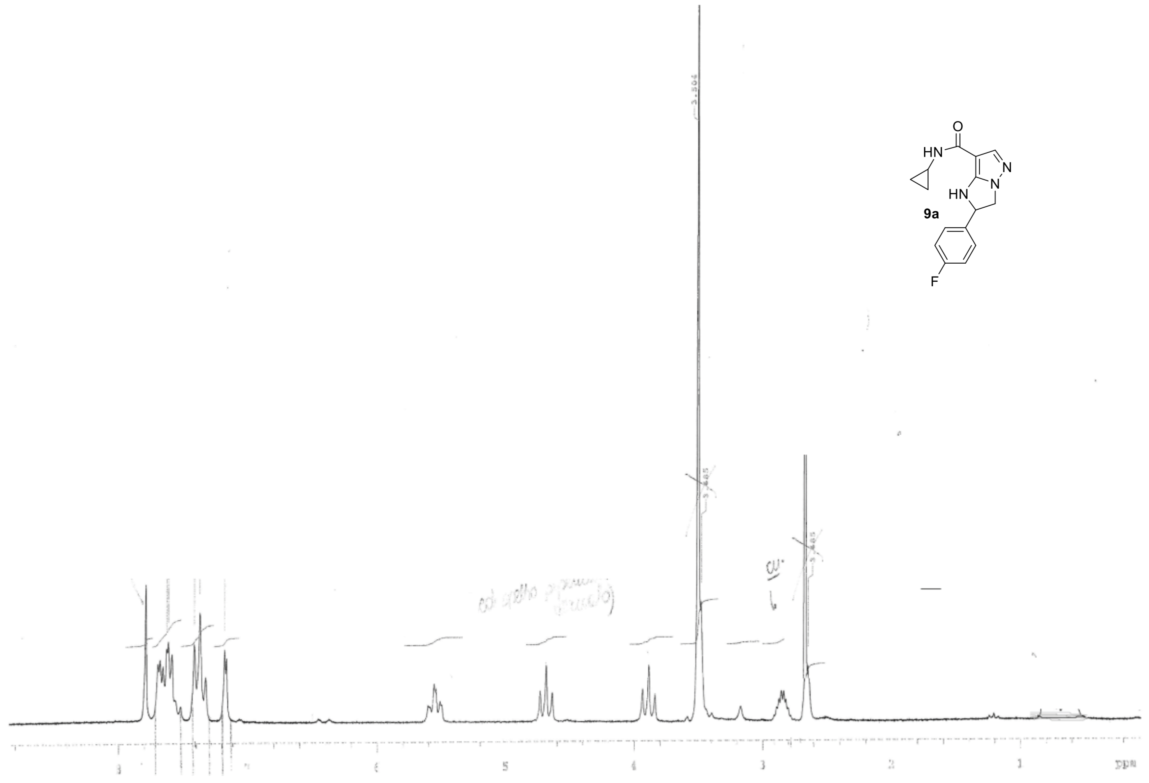
O
HN
HN
9a
F

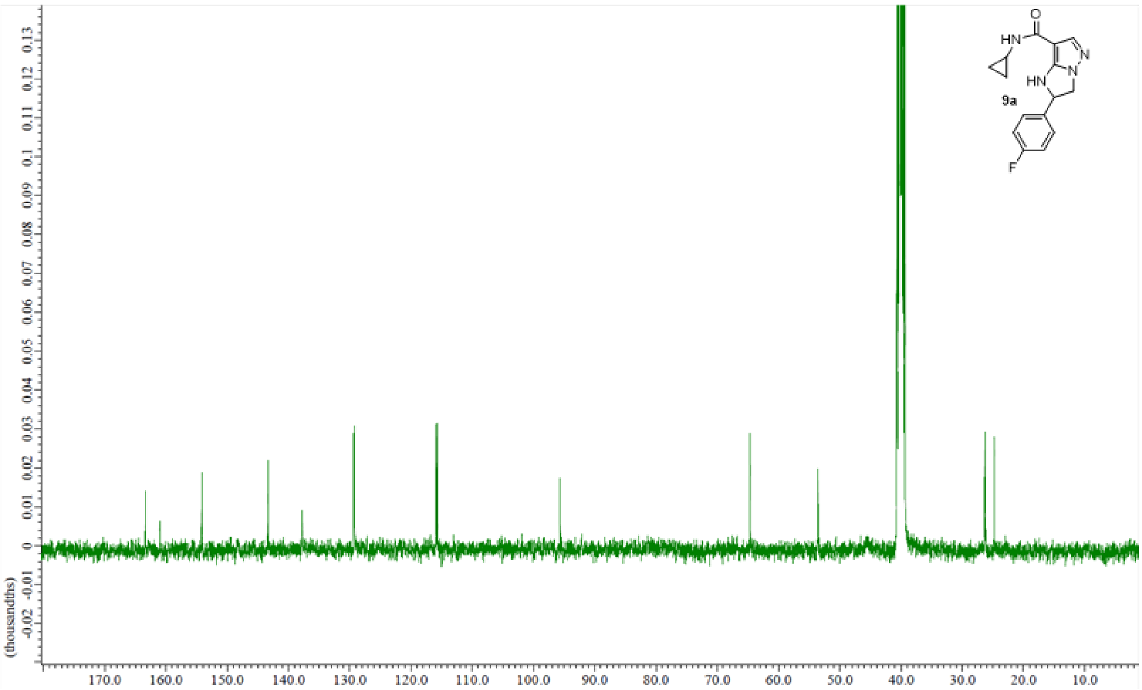
9a
(thousandths)
170.0 160.0 150.0 140.0 130.0 120.0 110.0 100.0 90.0 80.0 70.0 60.0 50.0 40.0 30.0 20.0 10.0

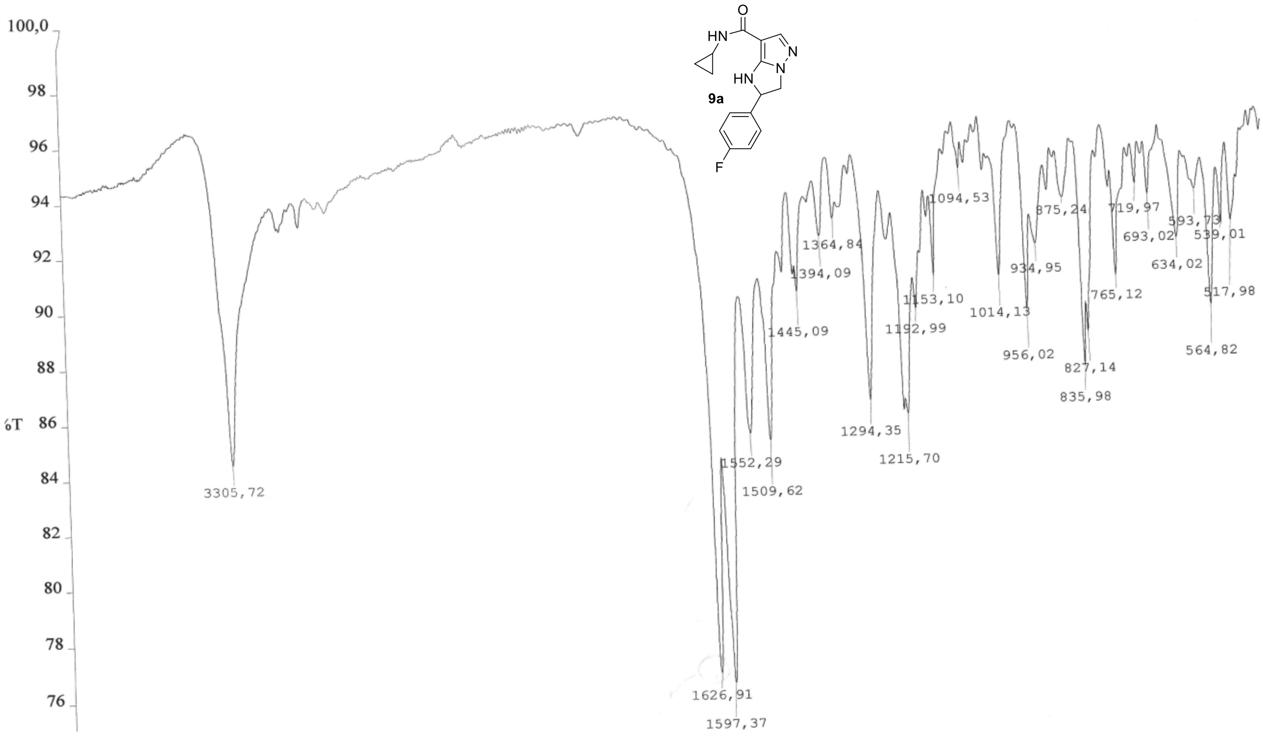
9a
%T
3305,72
1626,91
1597,37
1552,29
1509,62
1445,09
1394,09
1364,84
1294,35
1215,70
1192,99
1153,10
1094,53
1014,13
956,02
934,95
875,24
835,98
827,14
765,12
719,97
693,02
634,02
593,73
564,82
539,01
517,98

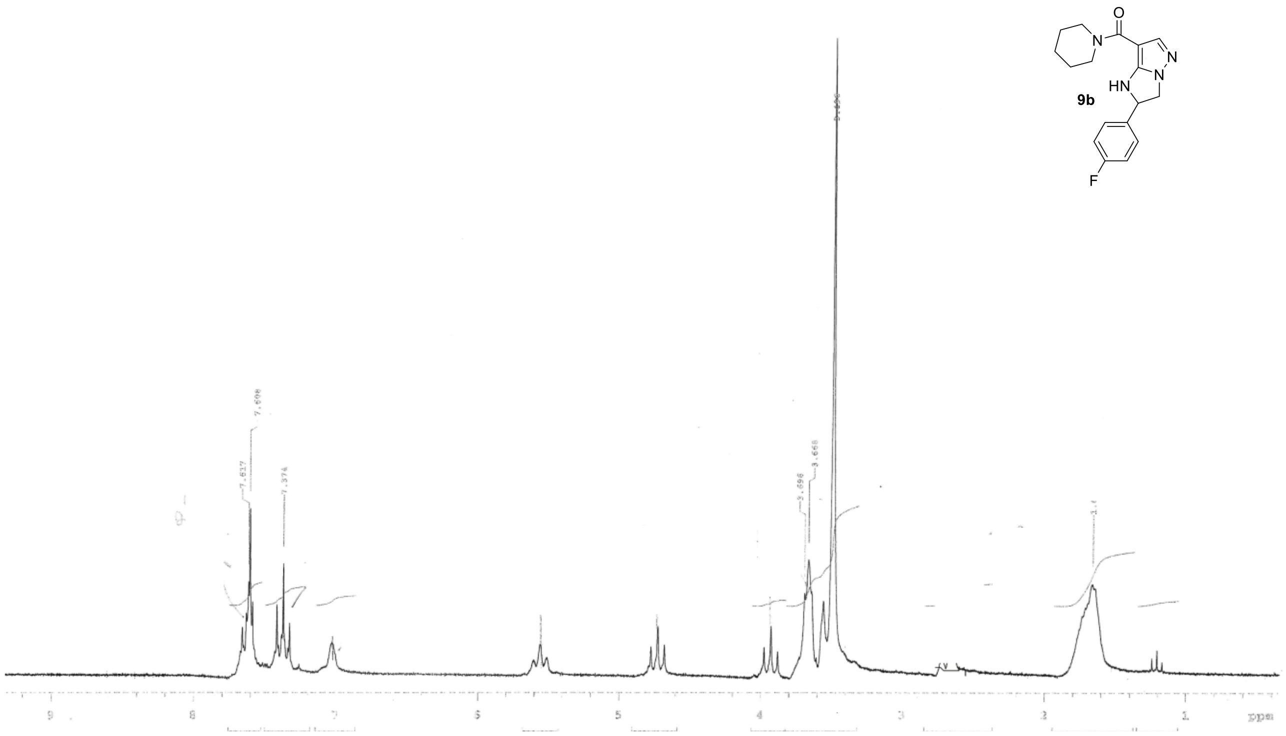
O
N
N
N
HN
9b
F
9
8
7
6
5
4
3
2
1
ppm

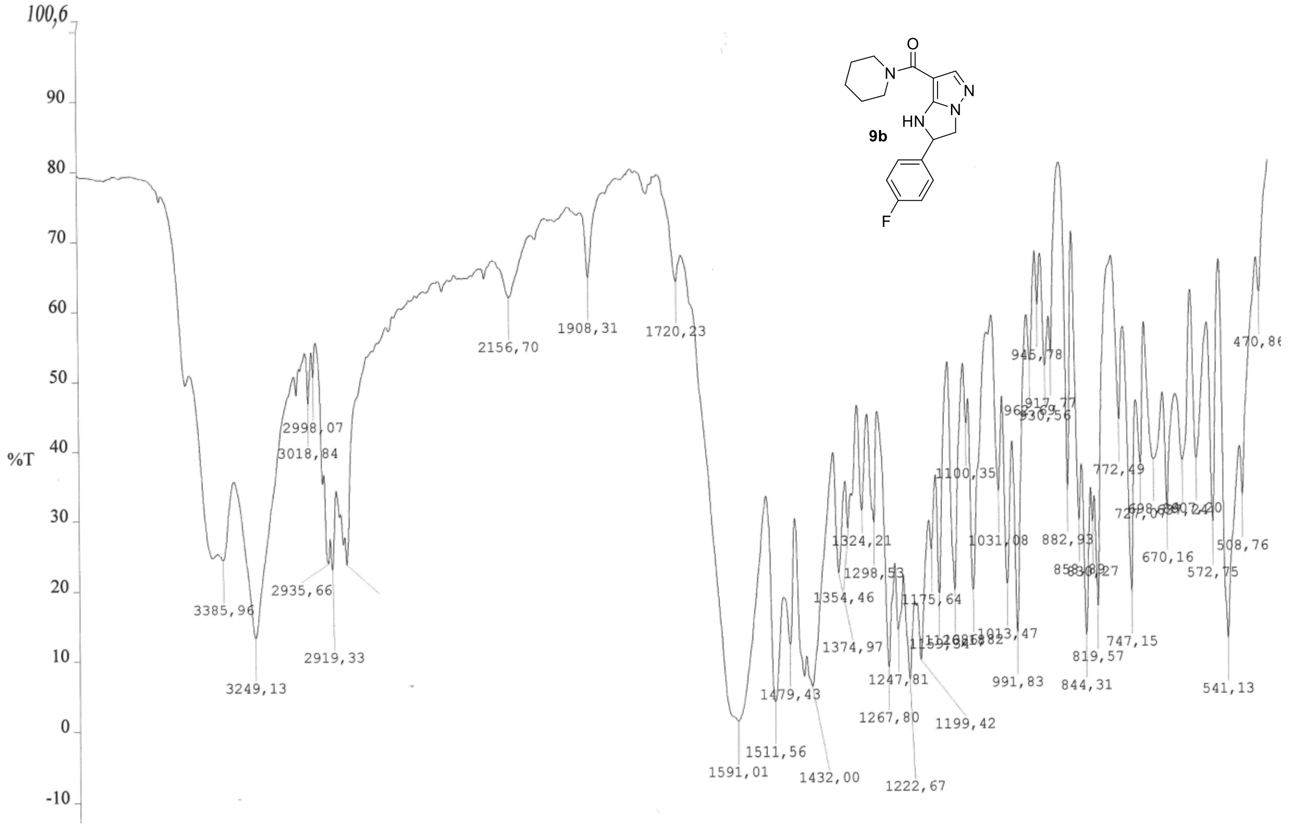
100,6
90
80
70
60
50
40
30
20
10
0
-10
%T
O
N
N
N
HN
9b
F
3385,96
3249,13
2998,07
3018,84
2935,66
2919,33
2156,70
1908,31
1720,23
1591,01
1511,56
1479,43
1432,00
1374,97
1354,46
1324,21
1298,53
1267,80
1247,81
1222,67
1199,42
1175,64
1100,35
1031,08
1013,47
991,83
962,69
945,78
930,56
917,77
882,93
844,31
819,57
772,49
747,15
670,16
572,75
541,13
508,76
470,86

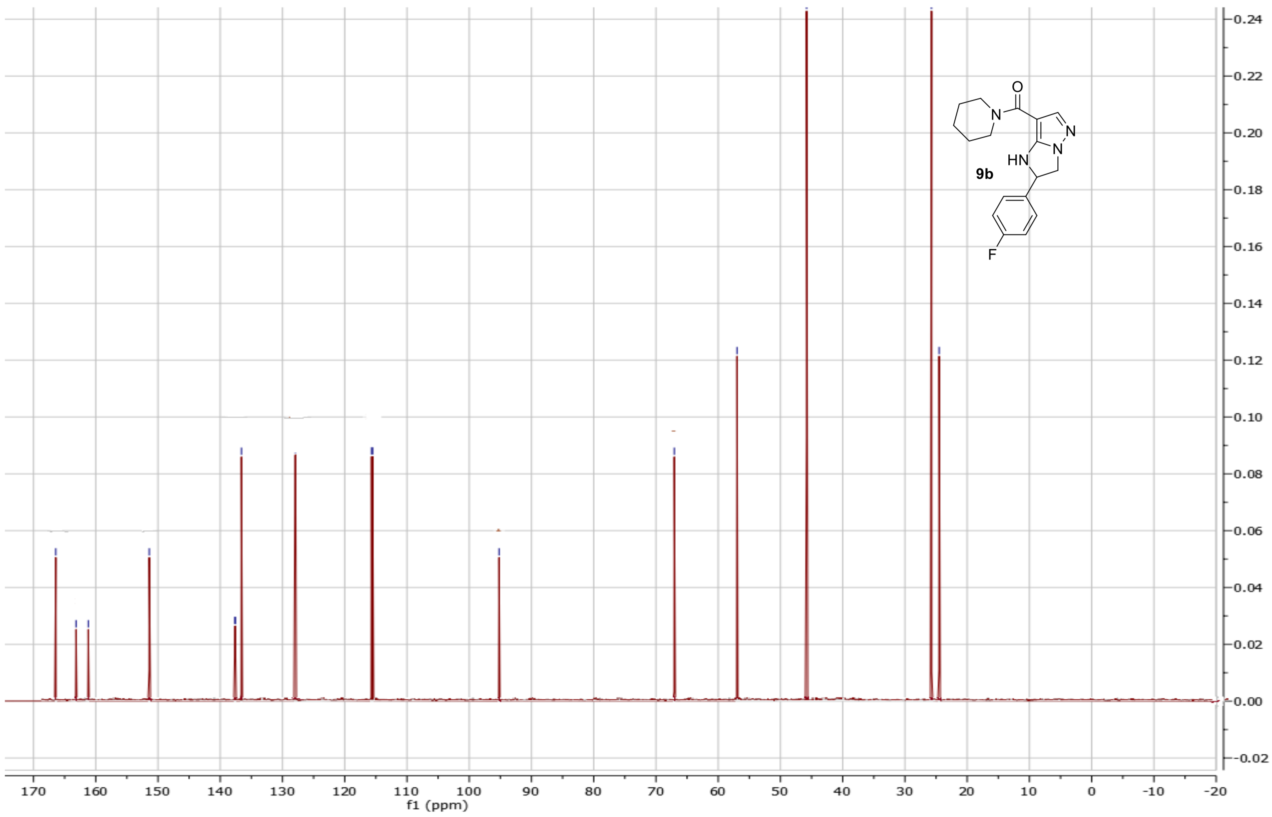

9b
f1 (ppm)

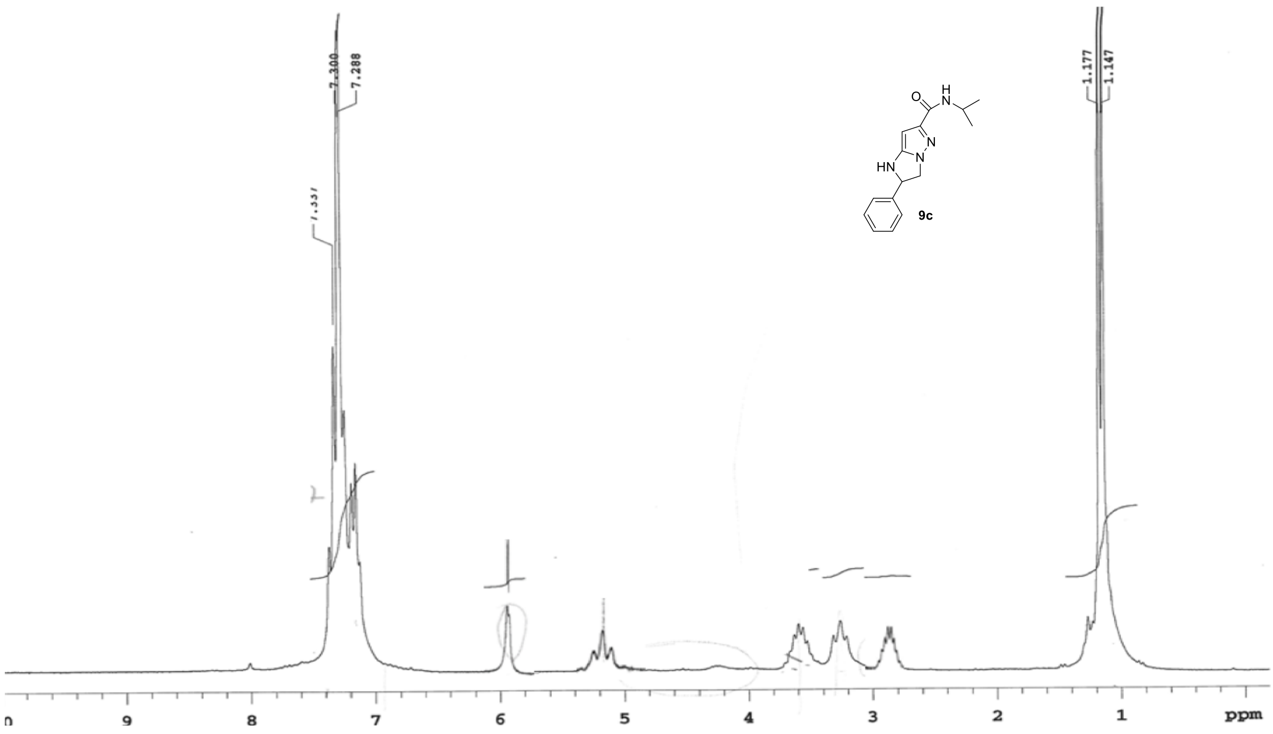

7.300
7.288
1.177
1.147
9c
ppm

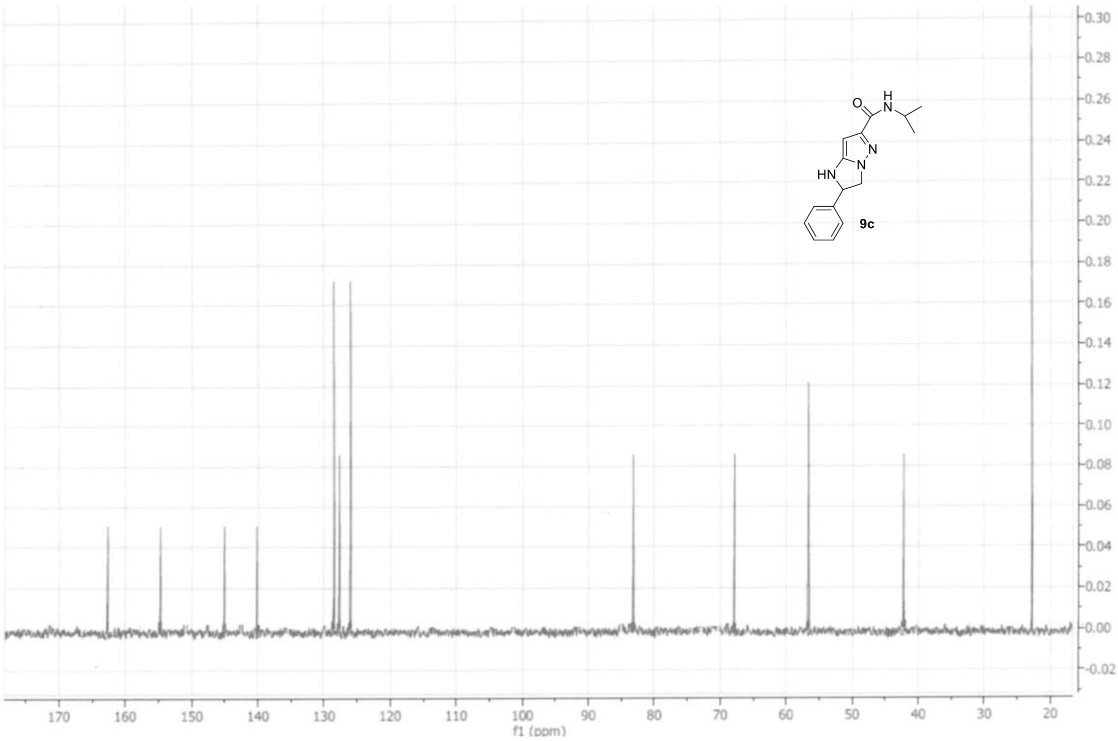
9c
f1 (ppm)
170
160
150
140
130
120
110
100
90
80
70
60
50
40
30
20
0.30
0.28
0.26
0.24
0.22
0.20
0.18
0.16
0.14
0.12
0.10
0.08
0.06
0.04
0.02
0.00
-0.02

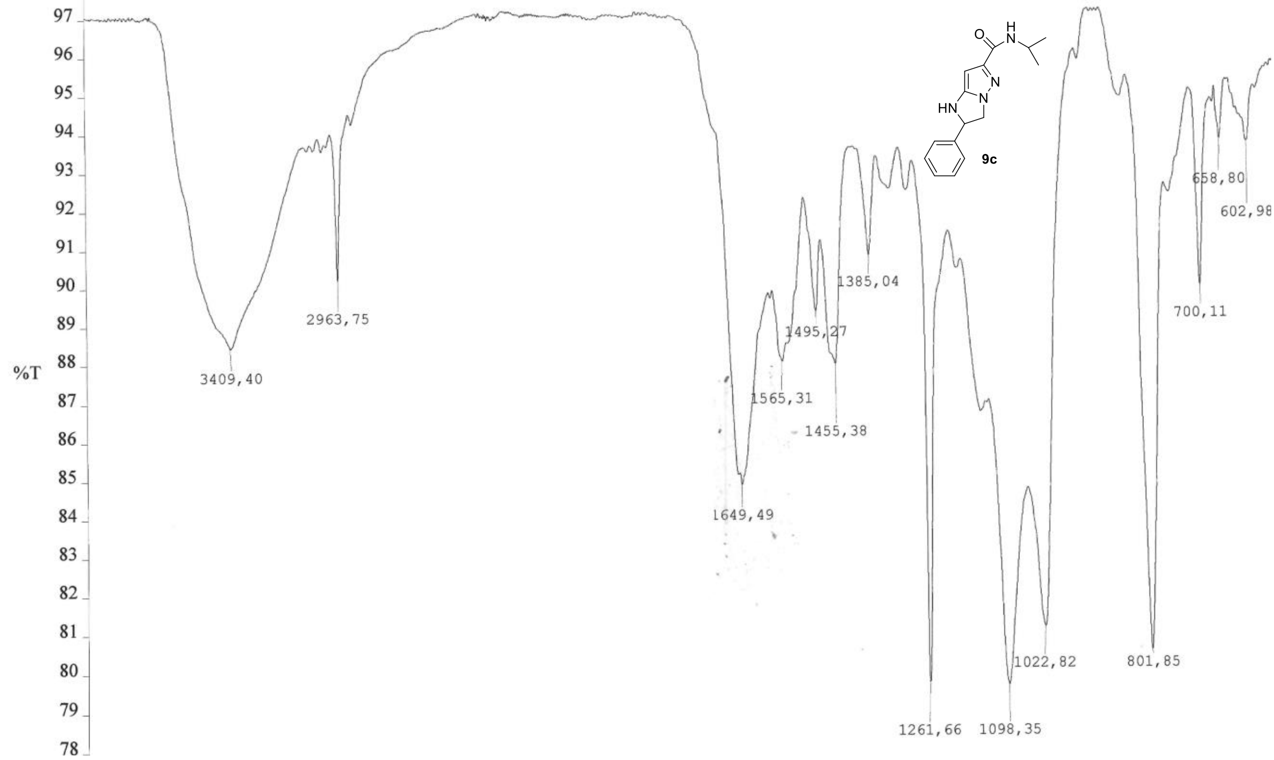
9c
%T
97
96
95
94
93
92
91
90
89
88
87
86
85
84
83
82
81
80
79
78
3409,40
2963,75
1649,49
1565,31
1495,27
1455,38
1385,04
1261,66
1098,35
1022,82
801,85
700,11
658,80
602,98

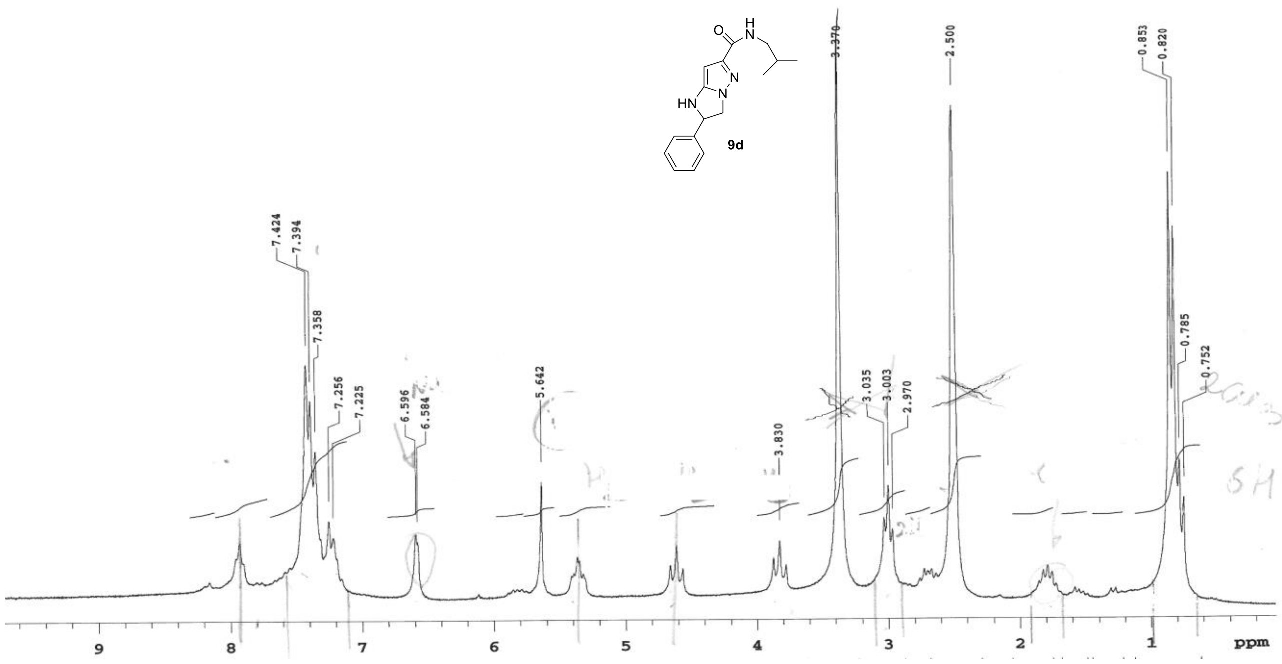

9d
7.424
7.394
7.358
7.256
7.225
6.596
6.584
5.642
3.830
3.370
3.035
3.003
2.970
2.500
0.853
0.820
0.785
0.752
9
8
7
6
5
4
3
2
1
ppm

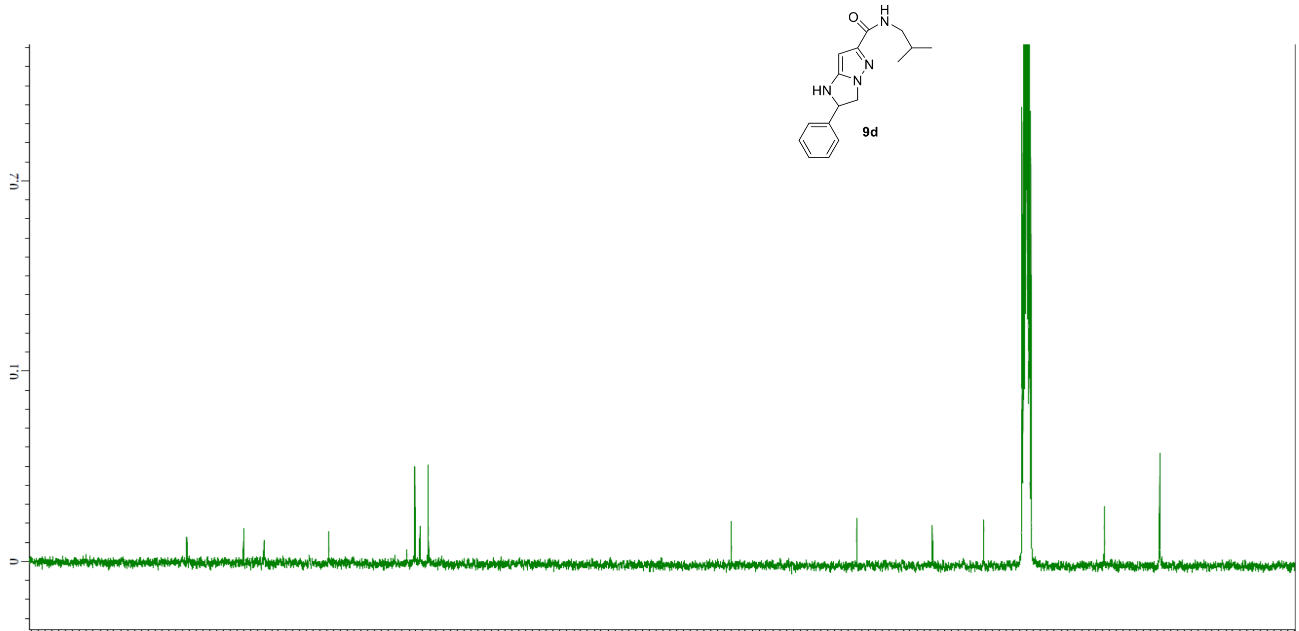

9d
180.0
170.0
160.0
150.0
140.0
130.0
120.0
110.0
100.0
90.0
80.0
70.0
60.0
50.0
40.0
30.0
20.0
10.0

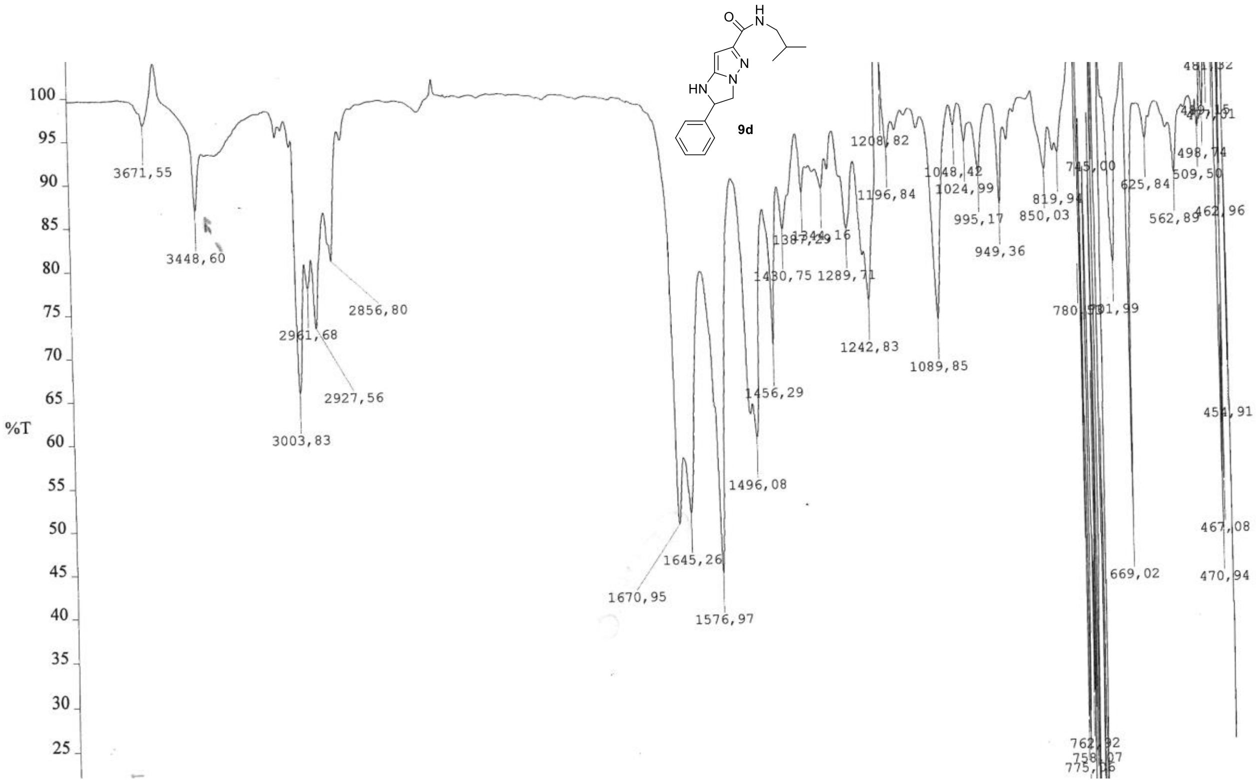
9d
%T
100
95
90
85
80
75
70
65
60
55
50
45
40
35
30
25
3671,55
3448,60
3003,83
2961,68
2927,56
2856,80
1670,95
1645,26
1576,97
1496,08
1456,29
1430,75
1289,71
1242,83
1208,82
1196,84
1089,85
1048,42
1024,99
995,17
949,36
850,03
819,94
669,02
625,84
562,89
509,50
498,74
462,96
467,08
470,94

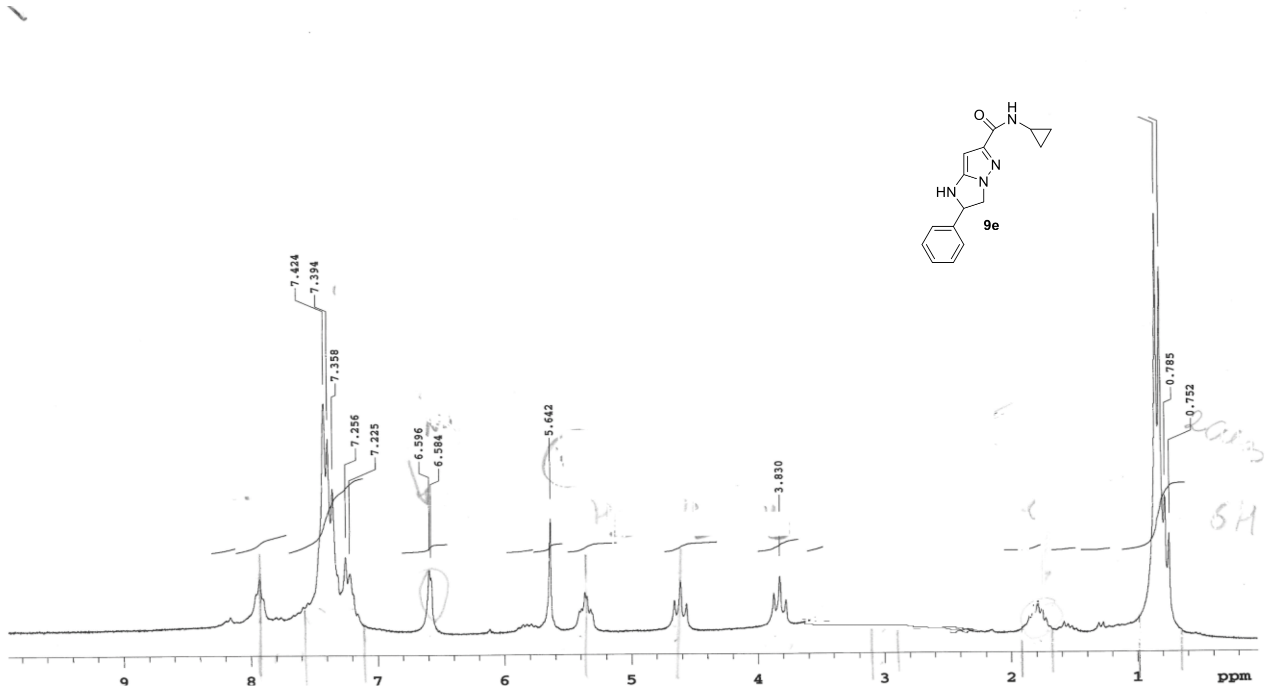
9e
7.424
7.394
7.358
7.256
7.225
6.596
6.584
5.642
3.830
0.785
0.752
6H
9
8
7
6
5
4
3
2
1
ppm

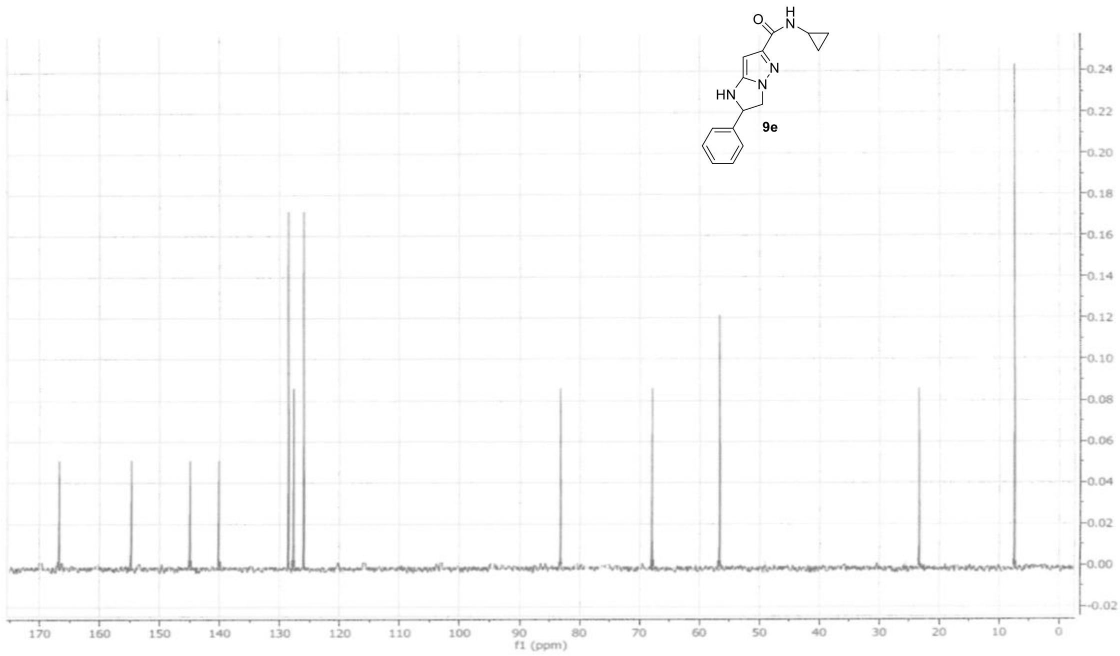

9e
f1 (ppm)
170 160 150 140 130 120 110 100 90 80 70 60 50 40 30 20 10 0
0.24 0.22 0.20 0.18 0.16 0.14 0.12 0.10 0.08 0.06 0.04 0.02 0.00 -0.02

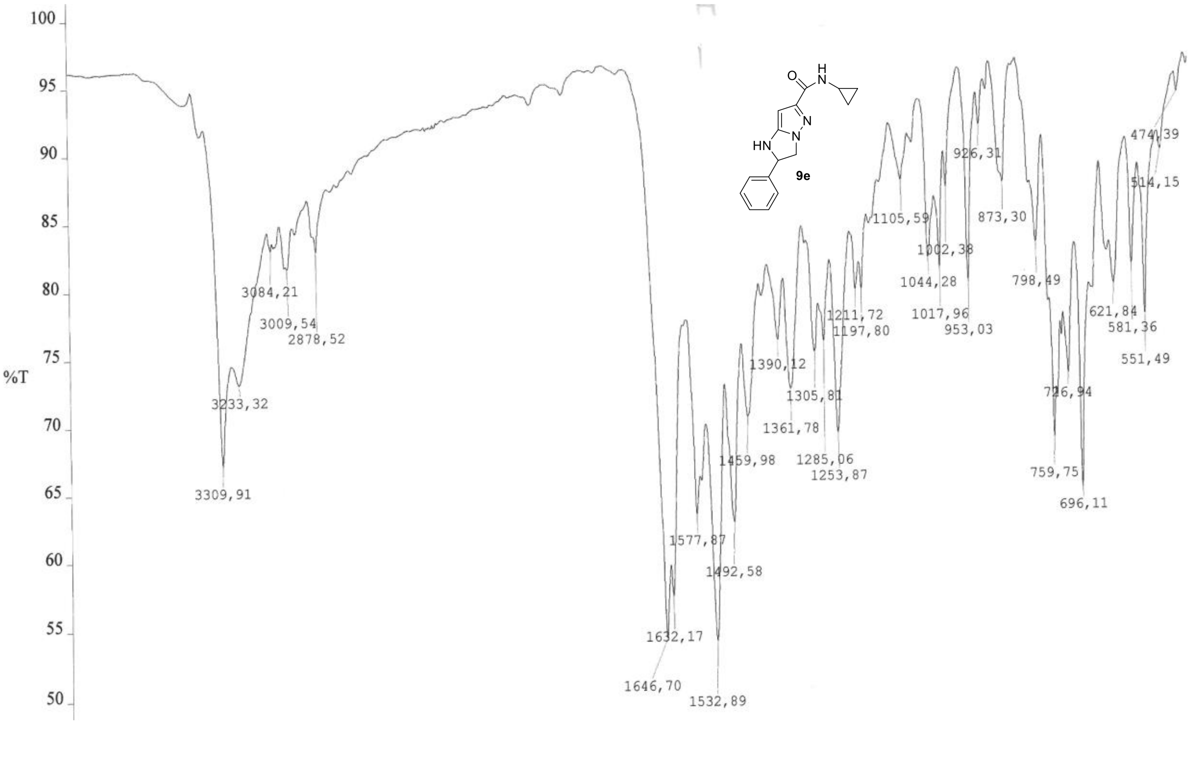

9e
%T
100 95 90 85 80 75 70 65 60 55 50
3309,91
3233,32
3084,21
3009,54
2878,52
1646,70
1632,17
1577,87
1532,89
1492,58
1459,98
1390,12
1361,78
1305,81
1285,06
1253,87
1211,72
1197,80
1105,59
1044,28
1002,38
1017,96
953,03
926,31
873,30
798,49
759,75
726,94
696,11
621,84
581,36
551,49
514,15
474,39

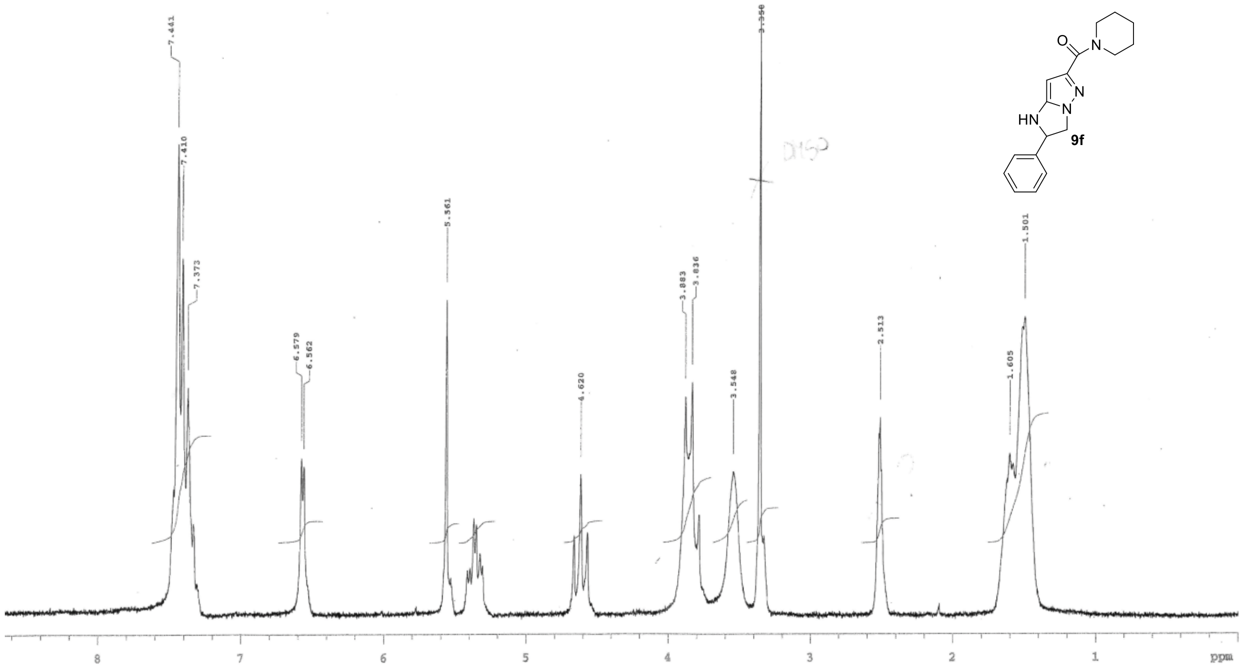
7.441
7.410
7.373
6.579
6.562
5.561
4.620
3.883
3.836
3.548
2.513
1.605
1.501
ppm
9f
HN
O
N

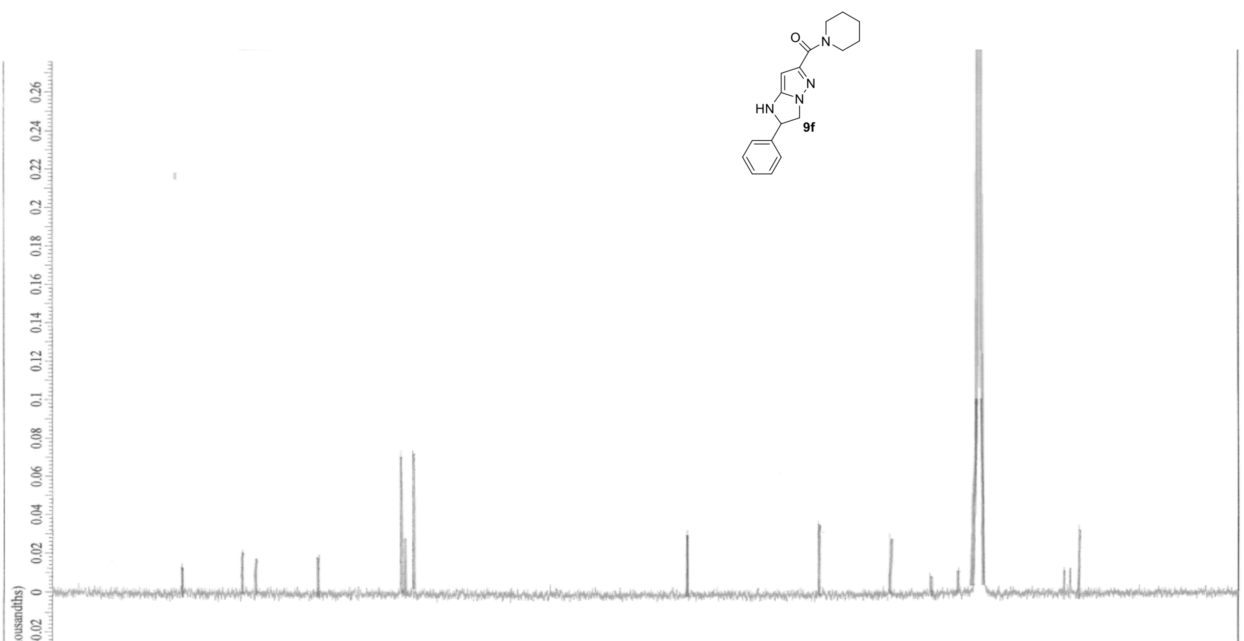
(thousandths)
170.0 160.0 150.0 140.0 130.0 120.0 110.0 100.0 90.0 80.0 70.0 60.0 50.0 40.0 30.0 20.0 10.0
9f
HN
O
N

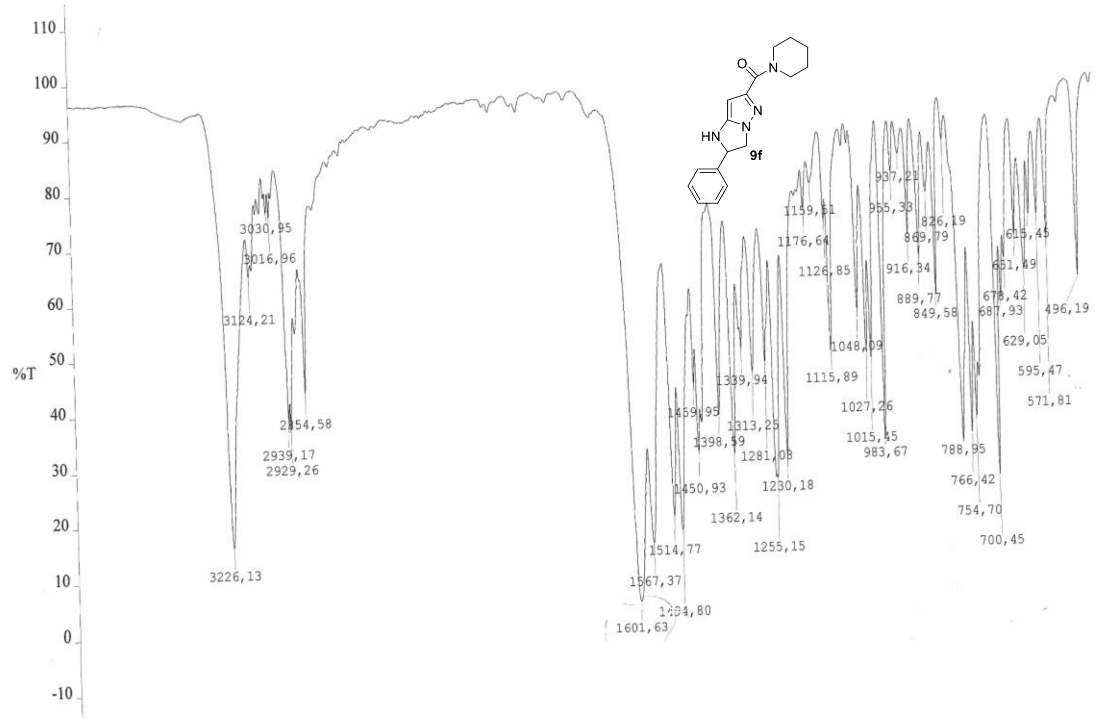
110
100
90
80
70
60
50
40
30
20
10
0
-10
%T
9f
HN
3226,13
3124,21
3030,95
3016,96
2939,17
2929,26
2854,58
1601,63
1567,37
1514,77
1494,80
1459,95
1450,93
1398,59
1362,14
1339,94
1313,25
1281,08
1255,15
1230,18
1176,64
1159,61
1126,85
1115,89
1048,09
1027,26
1015,45
983,67
955,33
937,21
916,34
889,77
869,79
849,58
826,19
788,95
766,42
754,70
700,45
687,93
678,42
651,49
629,05
615,45
595,47
571,81
496,19

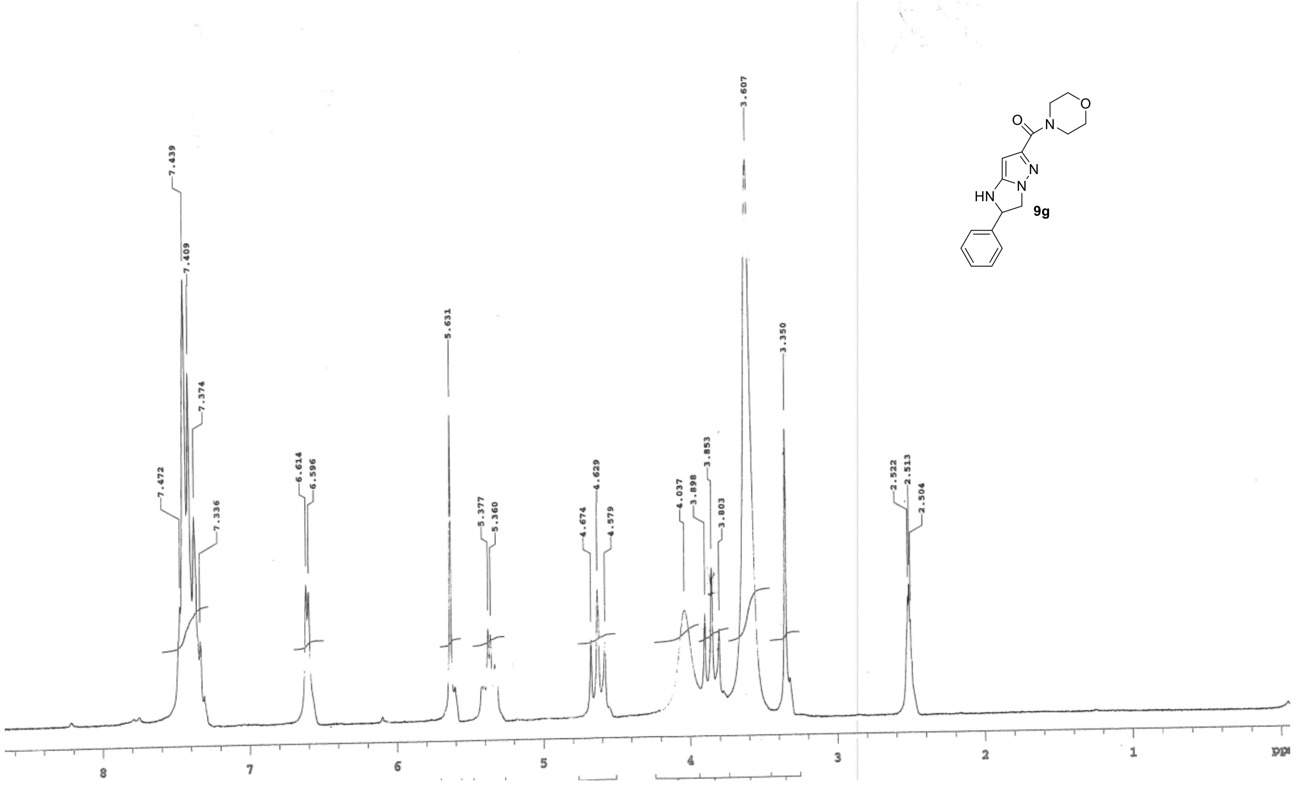
9g
HN
7.472
7.439
7.409
7.374
7.336
6.614
6.596
5.631
5.377
5.360
4.674
4.629
4.579
4.037
3.898
3.853
3.803
3.607
3.350
2.522
2.513
2.504
8
7
6
5
4
3
2
1
ppm

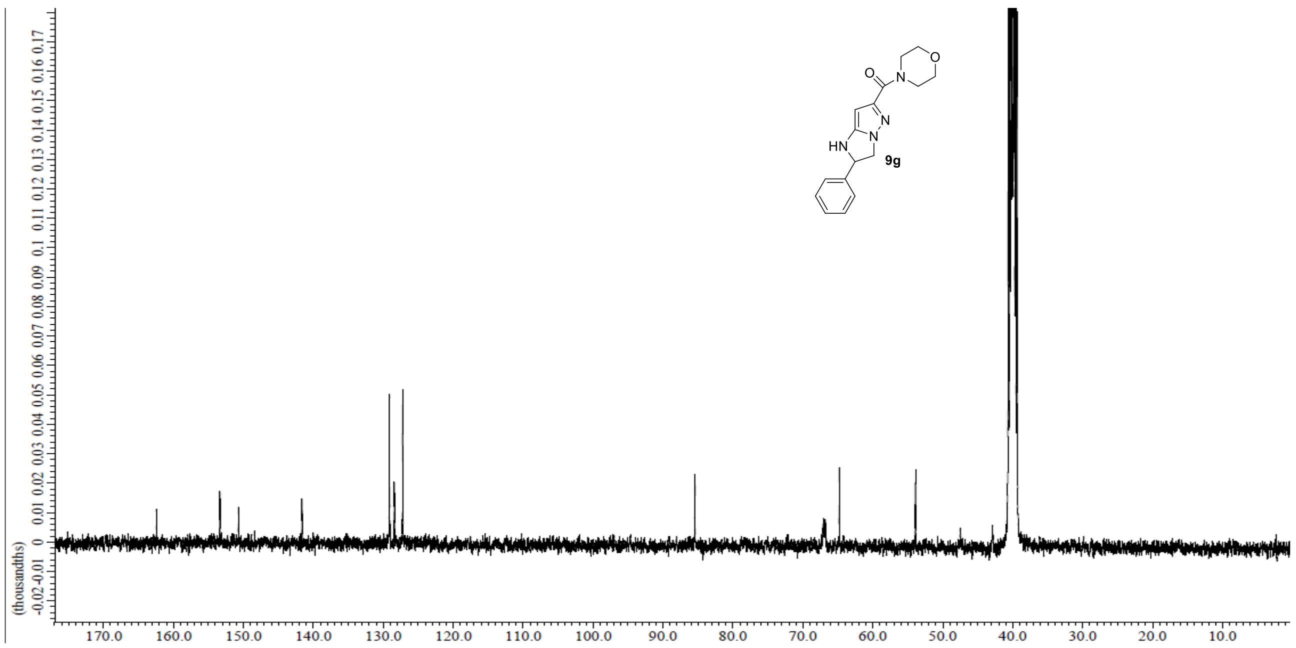
9g
HN
N
O
(thousandths)
170.0 160.0 150.0 140.0 130.0 120.0 110.0 100.0 90.0 80.0 70.0 60.0 50.0 40.0 30.0 20.0 10.0

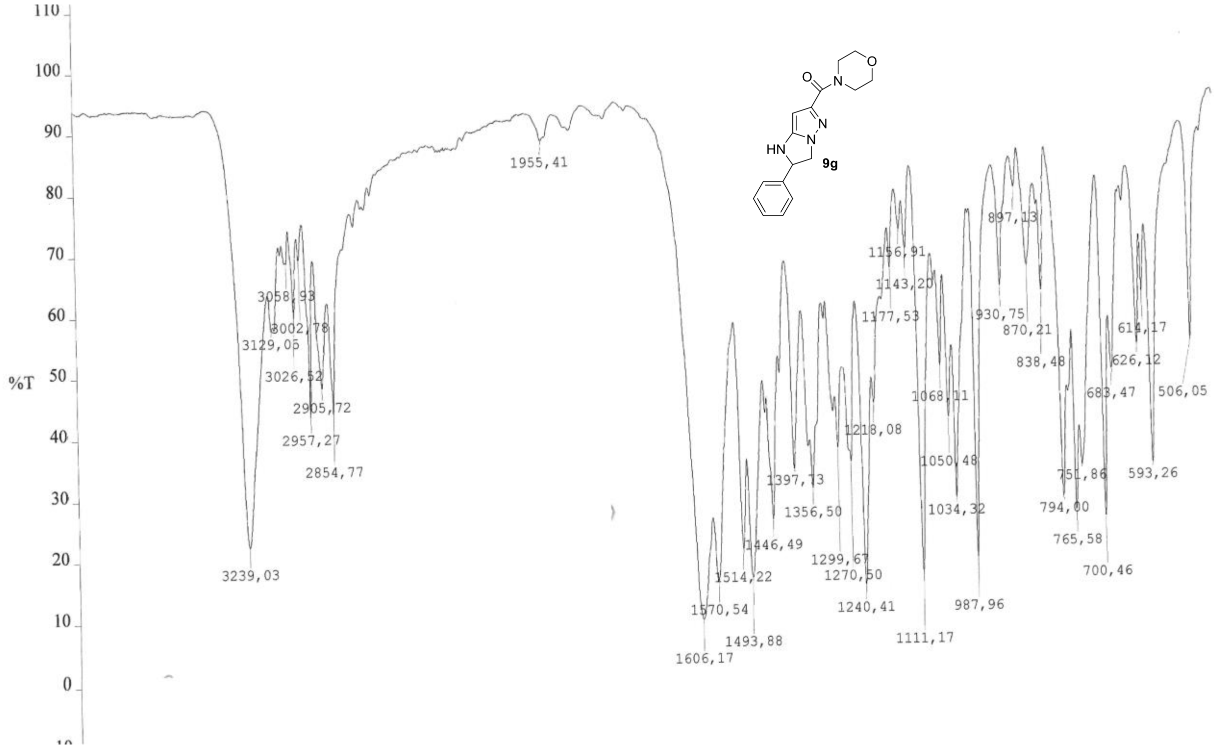
%T
9g
3239,03
3129,05
3058,93
3026,52
3002,78
2957,27
2905,72
2854,77
1955,41
1606,17
1570,54
1514,22
1493,88
1446,49
1397,73
1356,50
1299,67
1270,50
1240,41
1218,08
1177,53
1156,91
1143,20
1111,17
1068,11
1050,48
1034,32
987,96
930,75
897,13
870,21
838,48
794,80
765,58
751,86
700,46
683,47
626,12
614,17
593,26
506,05

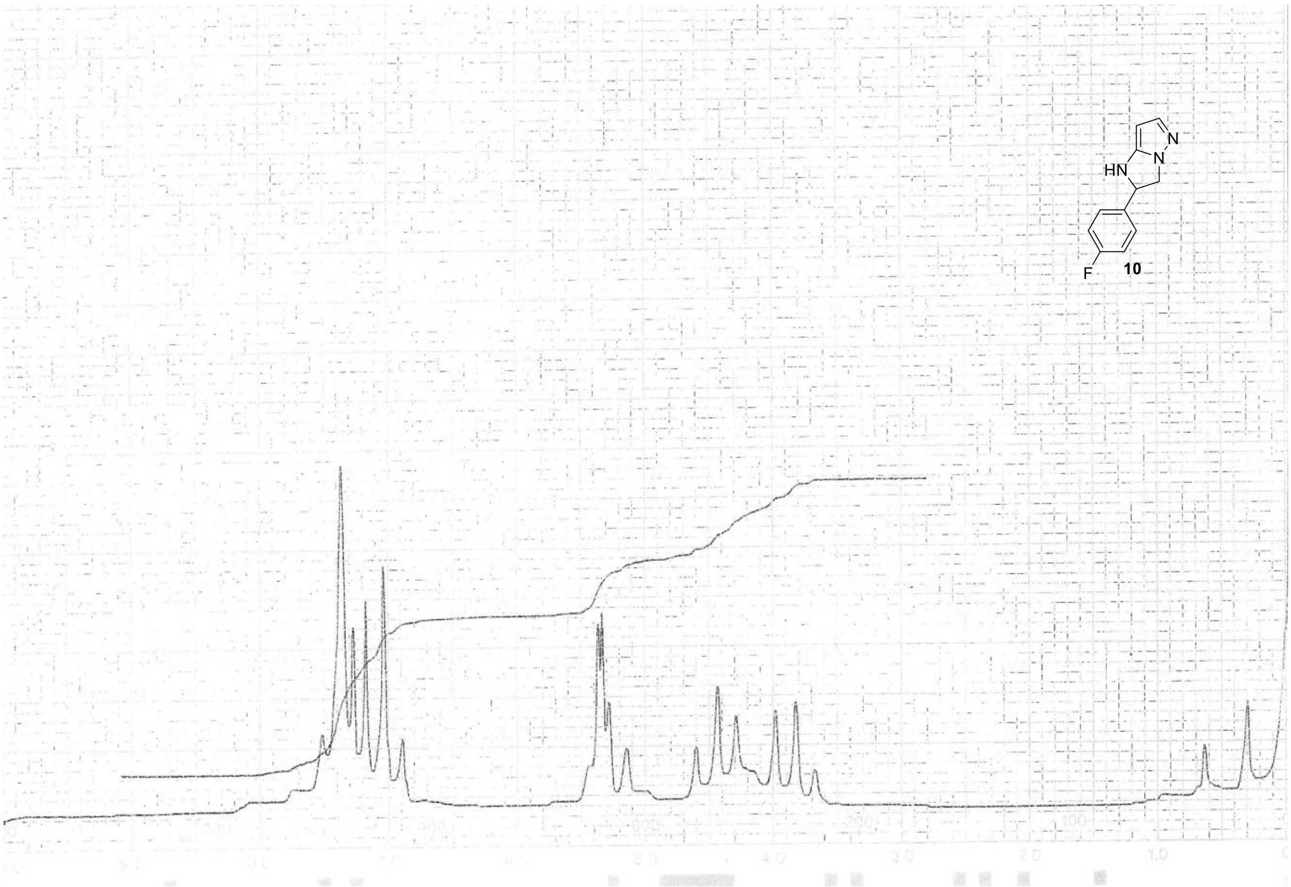
HN
N
N
F
10

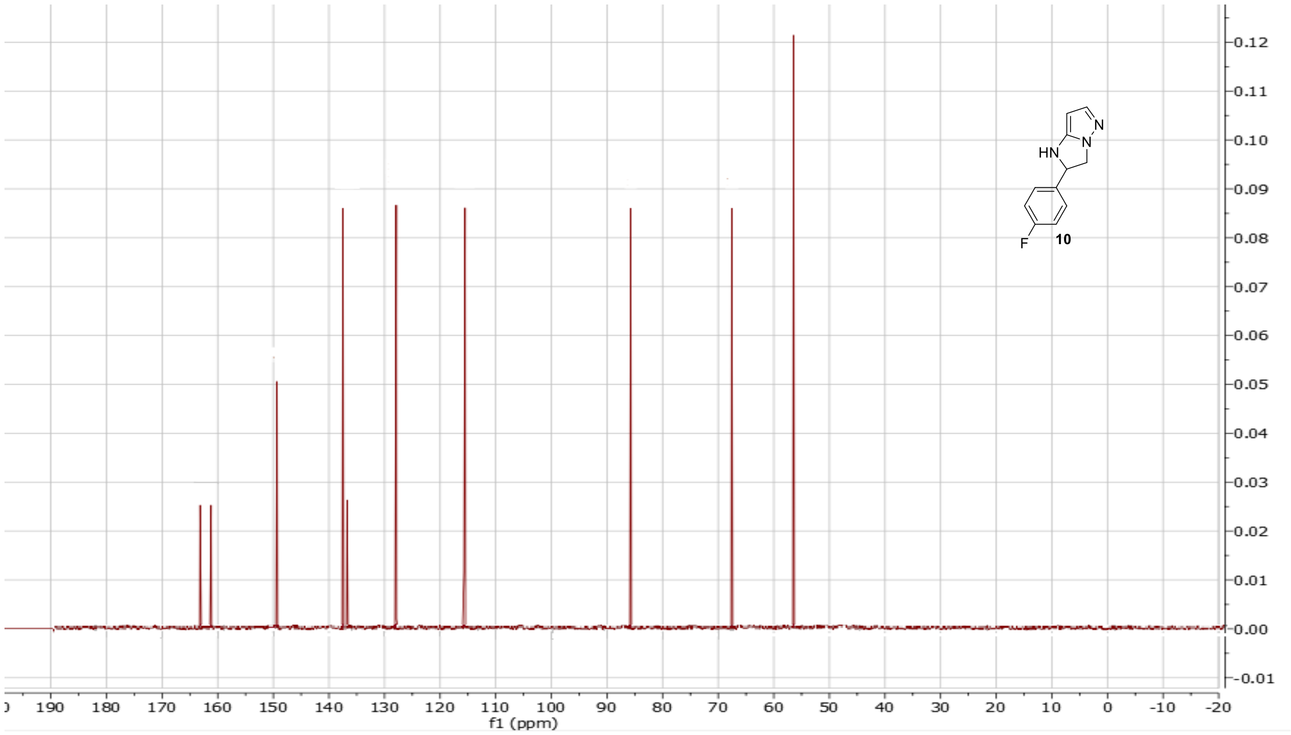
HN
N
N
F
10
f1 (ppm)

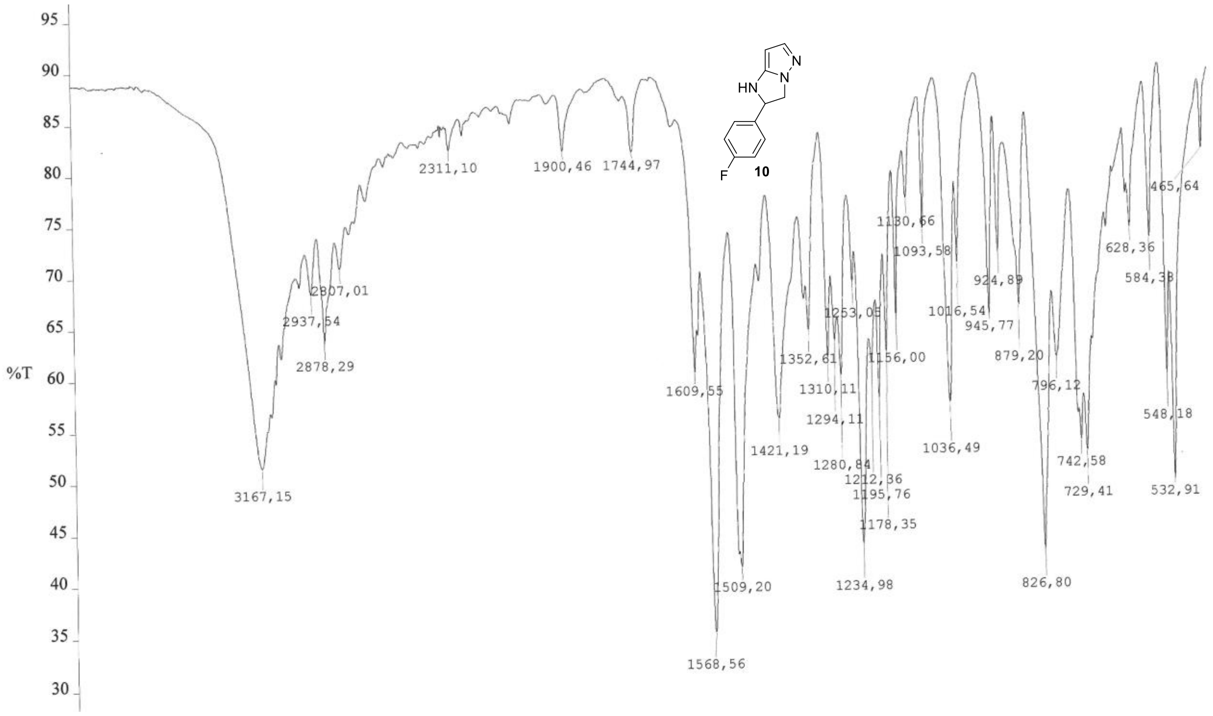
%T
95
90
85
80
75
70
65
60
55
50
45
40
35
30
HN
N
N
F
10
3167,15
2937,54
2878,29
2807,01
2311,10
1900,46
1744,97
1609,55
1568,56
1509,20
1421,19
1352,61
1310,11
1294,11
1280,84
1253,05
1234,98
1212,36
1195,76
1178,35
1156,00
1130,66
1093,58
1036,49
1016,54
945,77
924,89
879,20
826,80
796,12
742,58
729,41
628,36
584,38
548,18
532,91
465,64

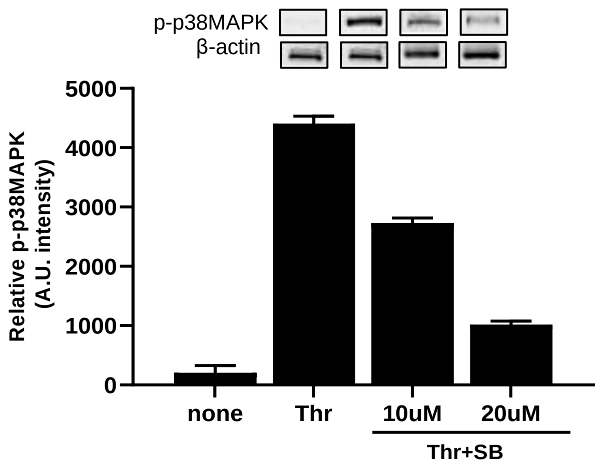

Figure S1. Immunoblotting densitometric image of p38MAPK phosphorylation in human platelets preincubated at 37°C with saline or SB203580 (SB) used as reference compound at two concentrations (10 and 20 μM).

Washed platelets (1.0×10^9 platelets/mL) were preincubated at 37°C with saline or SB203580 (SB) and then stimulated for 5 min with 0.1 U/mL thrombin (Thr). At the end of incubation suitable aliquots were immunoblotted with anti-p-p38MAPK as detailed in Methods. Blots are representative of four independent experiments. In the lower panels densitometric scanning ± SD of p38MAPK phosphorylation of four experiments is reported.
